# Trends in underweight, stunting, and wasting prevalence and inequality among children under three in Indian states, 1993–2016

**DOI:** 10.1038/s41598-021-93493-1

**Published:** 2021-07-08

**Authors:** Omar Karlsson, Rockli Kim, Rakesh Sarwal, K. S. James, S. V. Subramanian

**Affiliations:** 1 Takemi Program in International Health, Harvard T.H. Chan School of Public Health, Harvard University, 677 Huntington Avenue, Boston, MA 02115 USA; 2 Department of Economic History, School of Economics and Management, Lund University, P.O. Box 7083, 220 07 Lund, Sweden; 3 Division of Health Policy & Management, College of Health Science, Korea University, 145 Anam-ro, Seongbuk-gu, Seoul, 02841 Korea; 4 Harvard Center for Population and Development Studies, 9 Bow Street, Cambridge, MA 02138 USA; 5 National Institution for Transforming India (NITI) Aayog, Government of India, New Delhi, 110001 India; 6 International Institute for Population Sciences (IIPS), Deonar, Mumbai, 400088 India; 7 Department of Social and Behavioral Sciences, Harvard T.H. Chan School of Public Health, Harvard University, 677 Huntington Avenue, Boston, MA 02115 USA; 8 Interdisciplinary Program in Precision Public Health, Department of Public Health Sciences, Graduate School of Korea University, 145 Anam-ro, Seongbuk-gu, Seoul, 02841 Korea

**Keywords:** Infectious diseases, Nutrition disorders

## Abstract

Child undernutrition remains high in India with far-reaching consequences for child health and development. Anthropometry reflects undernutrition. We examined the state-level trends in underweight, stunting, and wasting prevalence and inequality by living standards using four rounds of the National Family Health Surveys in 26 states in India, conducted in 1992–1993, 1998–1999, 2005–2006, and 2015–2016. The average annual reduction (AAR) for underweight ranged from 0.04 percentage points (pp) (95% CI − 0.12, 0.20) in Haryana to 1.05 pp (95% CI 0.88, 1.22) in West Bengal for underweight; 0.35 pp (95% CI 0.11, 0.59) in Manipur to 1.47 (95% CI 1.19, 1.75) in Himachal Pradesh for stunting; and − 0.65 pp (95% CI − 0.77, − 0.52) in Haryana to 0.36 pp (95% CI 0.22, 0.51) in Bihar & Jharkhand for wasting. We find that change in the pp difference between children with the poorest and richest household living standards varied by states: statistically significant decline (increase) was observed in 5 (3) states for underweight, 5 (4) states for stunting, and 2 (1) states for wasting. Prevalence of poor anthropometric outcomes as well as disparities by states and living standards remain a problem in India.

## Introduction

Despite improvements, India’s children continue to face undernutrition and infections, which have permanent negative consequences for human development in terms of health and socioeconomic status^1,2^. Reduced weight gain and physical growth, and thinness—termed underweight, stunting, and wasting, respectively—reflect this adversity and are therefore used as proxy measures for undernutrition and exposure to infections at the population-level^3^. In India in 2016, 36% of children under five years old were underweight, 38% were stunted, and 21% suffered from wasting^4^. This makes India home to almost a third of the world’s stunted children^5^. Disparities by state^6^ and economic status^7^ further indicate that focusing on the most disadvantaged can significantly improve these outcomes.

Therefore, several initiatives aim to reduce the prevalence of underweight, stunting, and wasting in India: the Integrated Child Development Services Scheme (ICDS) provides education, food, and healthcare for mothers and children; The Targeted Public Distribution System (1997) subsidizes the price of food grains for the poor; The National Food Security Act (2013) enabled a shift from welfare to a rights-based approach to food security; and, more recently, the POSHAN Abhiyaan (2017) aims to improve nutrition among children and mothers^8^.

Most studies have focused on prevalence and inequality in underweight, stunting, and wasting for India overall. At the same time, states are an important independent unit in the Indian context, particularly since state governments are responsible for healthcare. Further, states vary enormously in terms of human development, with states such as Kerala, Chandigarh, and Goa having a high human development index, comparable to upper-middle-income countries such as Cuba and Mexico, while Jharkhand, Uttar Pradesh, and Bihar have a human development index comparable to lower-middle-income countries such as Pakistan and Angola^9,10^.

This paper studied trends in levels and disparities by household living standards in the prevalence of underweight, stunting, and wasting in Indian states between 1993 and 2016.

## Data and methods

### Data source

We used secondary data from four rounds of the National Family Health Surveys (NFHS), implemented by the International Institute for Population Sciences (IIPS) conducted 1992–1993, 1998–1999, 2005–2006, and 2015–2016 (referred to by the latter survey years in this paper). The NFHS are publicly accessible from the website of the Demographic and Health Surveys^11^.

### Sample population

The NFHS provides nationally representative household survey data using a multi-stage stratified sampling design. Females aged 15–49 were interviewed (13–49 in 1993), the heights of their children under five (or in some cases three) years old were measured, and information on their households was collected. The surveys had a response rate for households and females 15–49 (or 13–49), respectively, of 97.6% and 96.7% in 2016; 97.7% and 94.5% in 2006; 97.5% and 95.5% in 1999; and 95.6% and 96.1% in 1993.

Supplementary Table S1 shows sample sizes and missing values for each outcome in each state and survey year. Supplementary Table S2 compares age and the household wealth index (survey-year specific z-scores) of children with missing data and included children. Overall, there were small but statistically significant (i.e., 95% CI do not contain zero difference) wealth differences between included and excluded children, although the difference was larger in the older surveys. Children with missing information were around a year younger than children with no missing data in all but the earliest survey, where they were about 200 days younger.

### Outcomes

Children were defined as being underweight, stunted, and suffering from wasting if they had weight-for-age, height-for-age, and weight-for-height, respectively, below − 2 -scores (standard deviations) from the median of the WHO 2006 growth standard^3,12^. Children below − 2 z-scores would be among the 2.3% smallest children in the sample from which the WHO 2006 growth standard was created, representing healthy and well-off children from diverse settings across the globe. A stunting prevalence of 38% among children under 5 years old in India in 2016^4^, therefore, indicates widespread undernutrition and infections. Chronic undernutrition is considered more important for stunting and acute undernutrition for wasting. Underweight is regarded as a composite measure for both acute and chronic undernutrition. Infections play a particularly important role^13^. However, these indicators, particularly stunting and underweight, also indicate other environmental stressors, as well as being impacted by intergenerational mechanisms^14,15^.

Height was measured in millimeters. For children under 24 months old, the recumbent length was measured using an infantometer, while standing height was measured for children 24 months old and older using a stadiometer. Weight was measured in grams using a digital scale. Age was recorded in days. Children with unknown day of birth were assigned 15. Missing information on month and year of birth were randomly imputed after imposing logical ranges (e.g., based on other dates, birth intervals, and maternal age at birth, duration of amenorrhea, and abstinence) and constraints (e.g., age) by the DHS^16^.

Not all surveys measured the height of children over 36 months old, and our analysis was therefore restricted to children 0–36 months old in all surveys for comparability. Children with implausible values were excluded (weight-for-age z-score below − 6 or above 5, height-for-age z-scores below − 6 or above 6, and weight-for-height z-scores below − 5 or above 5)^17^.

### Household living standards

Living standards were measured using a survey-specific household wealth index provided in the NFHS—constructed from a principal component analysis, which combines the ownership of various assets (e.g., car, bicycle, refrigerator) and access to amenities (e.g., electricity, toilet facilities, water source) in the household in which the child resides, into a single continuous measure (i.e., component score)^18^.

The full sample of children (born 0–36 months before the survey) was divided into five equally sized groups (i.e., quintiles) according to the first component score (assumed to reflect household living standards), each group containing 20% of children within each state and survey (i.e., the living standards groups were state- and survey-specific). For pooled estimates, the wealth index quantiles were only survey-specific. We then compared the percentage point difference in the prevalence of underweight, stunting, and wasting between the 20% of children with the worst living standards (the poorest children) to the 20% of children with the best living standards (the richest children). We refer to the difference between the poorest and richest children as the poor–rich gap.

### State boundaries and inclusions

The geography of Indian states has changed between the 1993 and 2016 surveys. Chhattisgarh was created from Madhya Pradesh, Jharkhand was created from Bihar, Uttaranchal was created from Uttar Pradesh, and Telangana was created from Andhra Pradesh. We used states harmonized across surveys by IPUMS^19^.

We excluded union territories since data was only collected in the 2016 survey. Sikkim and Kashmir were not surveyed in 1993, and child height was not recorded for Tamil Nadu, West Bengal, Madhya Pradesh & Chhattisgarh, Andhra Pradesh & Telangana, and Himachal Pradesh in 1993.

### Analysis

To estimate the prevalence and average annual reduction (AAR), we first averaged our measures for each survey year and then used post estimation to obtain the AAR. The AAR shows how much, in percentage points (pp), the prevalence of underweight, stunting, and wasting declined per year on average in each state. The AAR was calculated by dividing the pp difference in prevalence between 2016 and 1993 by the average number of years (including decimals for months) between surveys for each state. The AAR was calculated using the 1999 survey in states without data in 1993.

For estimating the poor–rich gap for each state, we regressed each binary outcome variable (underweight, stunting, and wasting) on a constant, a dummy coded variable for wealth group, a dummy coded variable for survey year, and interactions terms for wealth group and survey year—using the richest children and the earliest survey year as omitted reference categories. The coefficient for the poorest children indicates the poor–rich gap in 1993 and the coefficients for the interaction term for the poorest children and the 2016 survey year shows by how much the poor–rich gap had changed in 2016, compared to the poor–rich gap in 1993.

All estimates were weighted using sampling weights. For all estimates, 95% confidence intervals (CIs) were obtained using robust standard errors adjusted for clustering at the primary sampling unit (PSU) level.

### Supplementary analyses

We did eight supplementary analyses. (1) We show Pearson's correlation coefficients for the relationship between prevalence in 1993 and the AAR (Supplementary Figs. S1–S3) and the relationship between the poor–rich gap in 1993 and change in the poor–rich gap between 1993 and 2016 (Supplementary Figs. S4–S6). (2) We estimated the average annual rate of reduction (AARR) in percentage terms (rather than percentage points) using instructions from UNICEF^20^ (Supplementary Figs. S7–S12). (3) We show the difference in our prevalence measures between children in the richest and poorest children as prevalence ratios (Supplementary Figs. S13–S18). (4) We show results using Erreygers concentration index (Supplementary Figs. S19–S21) and the modified concentration index (Supplementary Figs. S22–S24) to measure inequality in the prevalence of stunting across the entire distribution of household living standards, instead of a simple comparison between the richest and poorest 20% of children. (5) Since the season when the child was measured can have implications for undernutrition, particularly acute undernutrition^21^, we show our results after adjusting for the season when the interview took place, with the summer or pre-monsoon season (March–May) as a reference category (Supplementary Figs. S25–S27). (6) We show our results for males and females separately (Supplementary Figs. S28−S39). (7) We show results for severe underweight, severe stunting, and severe wasting defined as weight-for-age, height-for-age, and weight-for-height, respectively, below − 3 z-score (as opposed to − 2 z-score) according to the WHO 2006 growth standards (Supplementary Figs. S40–S45). (8) We show results for weight-for-age (instead of underweight), height-for-age (instead of stunting), and weight-for-height (instead of wasting) measured as z-score deviations from the WHO 2006 growth standard (Supplementary Figs. S46–S51).

### Ethical standards

This project used publicly accessible secondary data obtained from the DHS *website*. The DHS data are not collected specifically for this study and no one on the study team has access to identifiers linked to the data. These activities do not meet the regulatory definition of human subject research. As such, an Institutional Review Board (IRB) review is not required. The Harvard Longwood Campus IRB allows researchers to self-determine when their research does not meet the requirements for IRB oversight via guidance *online* regarding when an IRB application is required using an *IRB Decision Tool*. The ICF IRB and local IRBs approved data collection procedures and questionnaires and the U.S. Center for Disease Control and Prevention (CDC) reviewed protocols.

## Results

### Trends in anthropometric outcomes: a national view

**Figure 1:**
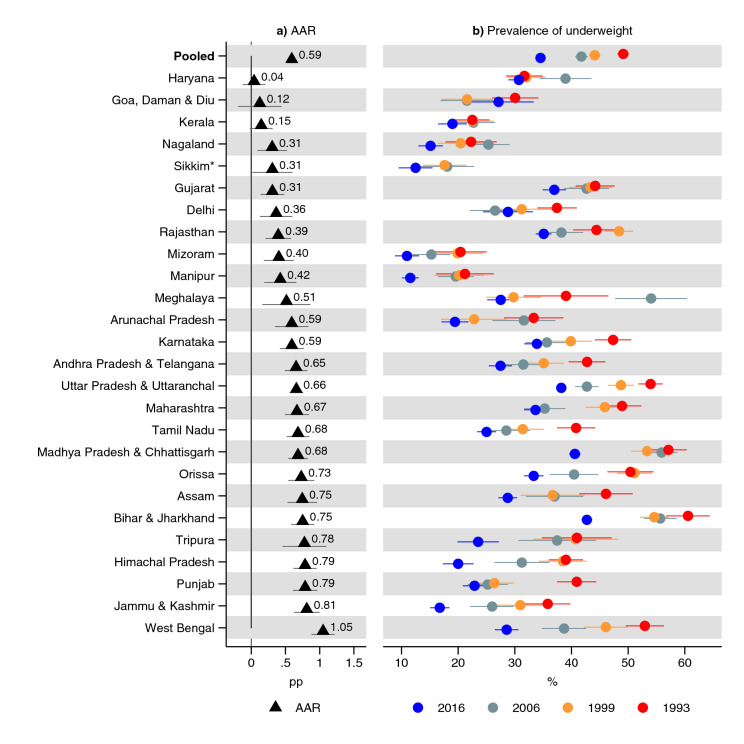
Prevalence of underweight and average annual reduction (AAR) in underweight between 1993 and 2016. *Indicates states with no data for 1993: 1999 was used instead. Average annual reduction (AAR) shows average annual percentage point (pp) reduction in prevalence of underweight in each state. See Table S3 for tabulated estimates. 95% confidence intervals are shown. Estimates are weighted using sampling weights and confidence intervals were adjusted for clustering at the PSU-level.

**Figure 2:**
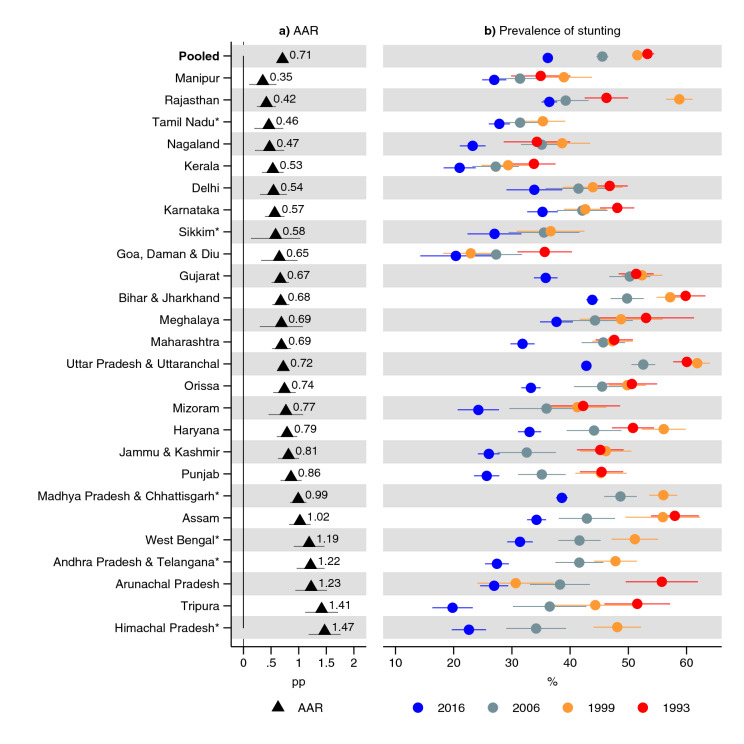
Prevalence of stunting and average annual reduction (AAR) in stunting between 1993 and 2016. *Indicates states with no data for 1993: 1999 was used instead. Average annual reduction (AAR) shows average annual percentage point (pp) reduction in prevalence of stunting in each state. See Table S4 for tabulated estimates. 95% confidence intervals are shown. Estimates are weighted using sampling weights and confidence intervals were adjusted for clustering at the PSU-level.

**Figure 3:**
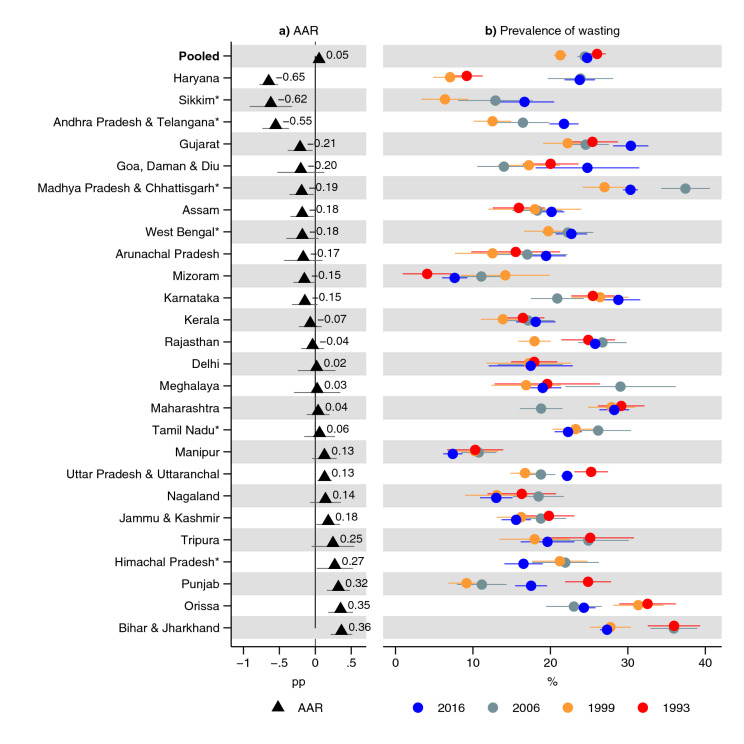
Prevalence of wasting and average annual reduction (AAR) in wasting between 1993 and 2016. *Indicates states with no data for 1993: 1999 was used instead. Average annual reduction (AAR) shows average annual percentage point (pp) reduction in prevalence of wasting in each state. See Table S5 for tabulated estimates. 95% confidence intervals are shown. Estimates are weighted using sampling weights and confidence intervals were adjusted for clustering at the PSU-level.

In India, overall, in 1993, 49% (95% CI 48, 50) of children 0–36 months old were underweight while 35% (95% CI 34, 35) were underweight in 2016 (Fig. 1 and Supplementary Table S3). The AAR in the prevalence of underweight was 0.6 (95% CI 0.6, 0.6) pp per year. In 1993, 53% (95% CI 52, 54) were stunted in India overall (Fig. 2 and Supplementary Table S4). With an AAR of 0.7 pp (95% CI 0.7, 0.8), the prevalence had declined to 36% (95% CI 36, 37) in 2016. The prevalence of wasting was 26% (95% CI 25, 27) in 1993, and, with an AAR of 0.05 (95% CI 0, 0.1) pp, declined to 25% (95% CI 24, 25) in 2016 (Fig. 3 and Supplementary Table S5).

**Figure 4:**
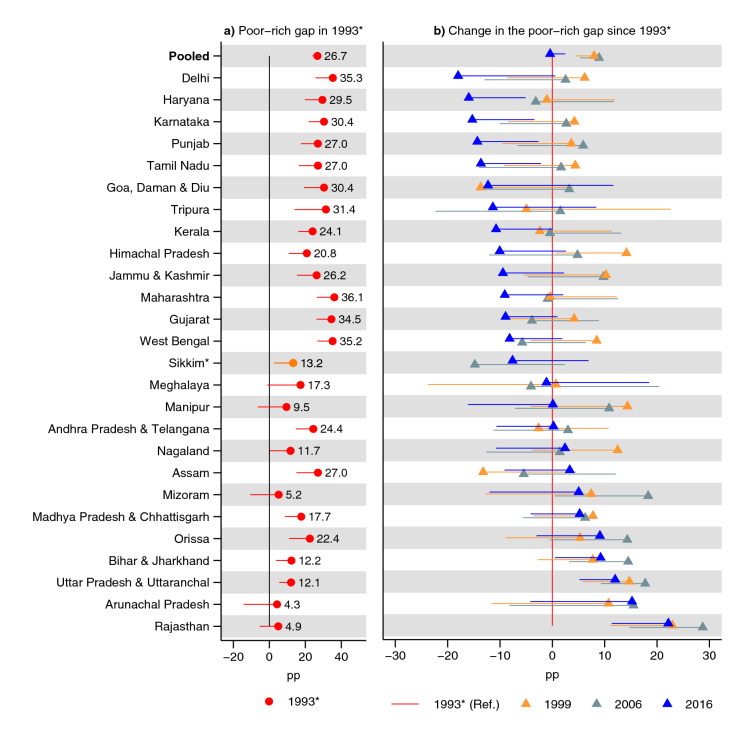
Changes in the poor–rich gap in prevalence of underweight. Percentage point (pp) differences are shown. In (**a**) a positive estimate indicates disadvantage for the poorest children, compared to the richest children, in 1993. A negative estimate in (**b**) indicates that the poor–rich gap (which usually shows a poor disadvantage in (**a**) has shrunk since 1993. The estimates were obtained from an interaction model (OLS) for each state: (**a**) shows the terms for the poorest wealth quintile and (**b**) shows the interaction terms (i.e., between poorest quintile and year). The terms for year as well as all terms involving quintiles other than the poorest are excluded from the figure. See Table S6 for tabulated estimates. Vertical lines (at 0) indicate no poor–rich difference in (**a**) and no change in rich–poor gap in (**b**). *Indicates states with no data for 1993: 1999 was used instead. 95% confidence intervals are shown. Only one confidence bound is shown to improve readability: an upper bound for estimates lower than 0 and a lower bound for estimates greater than zero. Estimates are weighted using sampling weights and confidence intervals were adjusted for clustering at the PSU-level.

**Figure 5:**
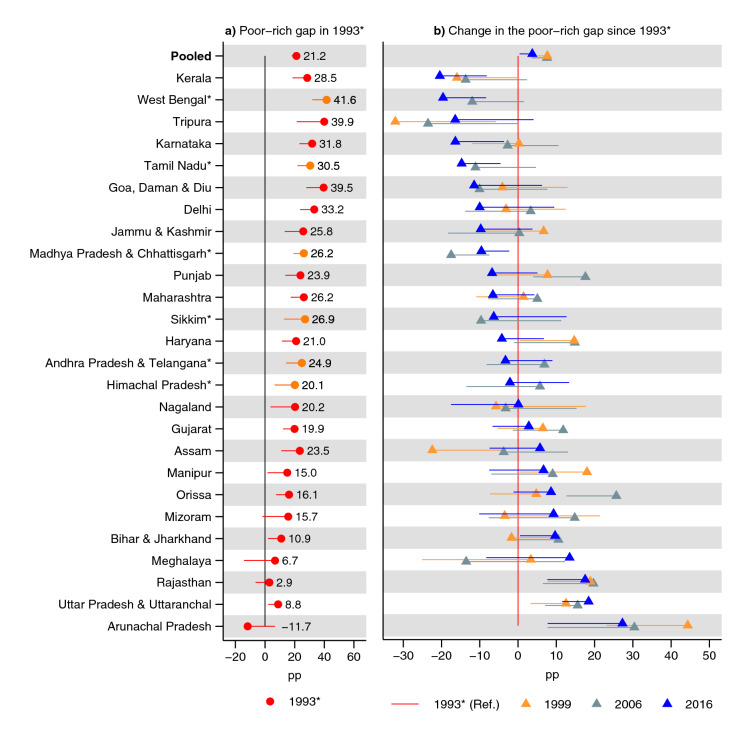
Changes in the poor–rich gap in prevalence of stunting. Percentage point (pp) differences are shown. In (**a**) a positive estimate indicates disadvantage for the poorest children, compared to the richest children, in 1993. A negative estimate in (**b**) indicates that the poor–rich gap (which usually shows a poor disadvantage in (**a**) has shrunk since 1993. The estimates were obtained from an interaction model (OLS) for each state: (**a**) shows the terms for the poorest wealth quintile and (**b**) shows the interaction terms (i.e., between poorest quintile and year). The terms for year as well as all terms involving quintiles other than the poorest are excluded from the figure. See Table S6 for tabulated estimates. Vertical lines (at 0) indicate no poor–rich difference in (**a**) and no change in rich–poor gap in (**b**). *Indicates states with no data for 1993: 1999 was used instead. 95% confidence intervals are shown. Only one confidence bound is shown to improve readability: an upper bound for estimates lower than 0 and a lower bound for estimates greater than zero. Estimates are weighted using sampling weights and confidence intervals were adjusted for clustering at the PSU-level.

**Figure 6:**
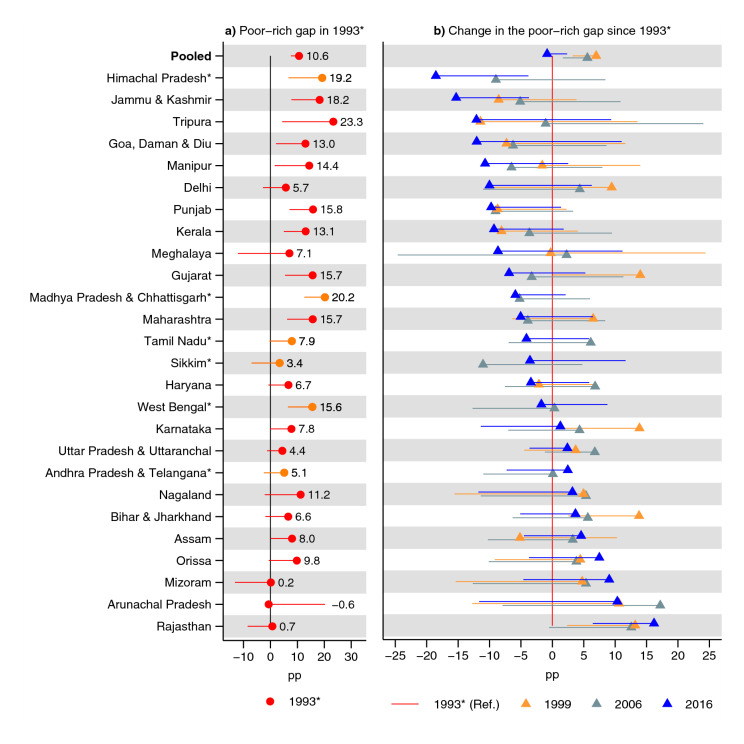
Changes in the poor–rich gap in prevalence of wasting. Percentage point (pp) differences are shown. In (**a**) a positive estimate indicates disadvantage for the poorest children, compared to the richest children, in 1993. A negative estimate in (**b**) indicates that the poor–rich gap (which usually shows a poor disadvantage in Panel a) has shrunk since 1993. The estimates were obtained from an interaction model (OLS) for each state: (**a**) shows the terms for the poorest wealth quintile and (**b**) shows the interaction terms (i.e, between poorest quintile and year). The terms for year as well as all terms involving quintiles other than the poorest are excluded from the figure. See Table S6 for tabulated estimates. Vertical lines (at 0) indicate no poor–rich difference in (**a**) and no change in rich–poor gap in (**b**). *Indicates states with no data for 1993: 1999 was used instead. 95% confidence intervals are shown. Only one confidence bound is shown to improve readability: an upper bound for estimates lower than 0 and a lower bound for estimates greater than zero. Estimates are weighted using sampling weights and confidence intervals were adjusted for clustering at the PSU-level.

In India overall in 1993, the poorest children had a 27 pp (95% CI 24, 29) higher prevalence of underweight (Fig. 4 and Supplementary Table S6); 21 (95% CI 18, 24) pp higher prevalence of stunting (Fig. 5 and Supplementary Table S6); and 11 (95% CI 8, 14) pp higher prevalence of wasting than the richest children (Fig. 6 and Supplementary Table S6). In India overall, there was no substantial change in the poor–rich gap for underweight (0 pp, 95% CI − 3, 2) and wasting (− 1 pp, 95% CI − 4, 2) while there was an increase in the advantage of the richest children for the prevalence of stunting (4 pp, 95% CI 1, 7).

### State-specific trends in anthropometric outcomes

The underweight prevalence declined in all states between 1993 and 2016, although the decline was not uniform over time in every state (Fig. 1 and Supplementary Table S3). The 95% CI for AAR also contained 0 in three states. The AAR for prevalence of underweight was the greatest in West Bengal (1.1 pp; 95% CI 0.9, 1.2) and Jammu & Kashmir (0.8 pp; 95% CI 0.6, 1.0), and the lowest in Haryana (0.04 pp, 95% CI − 0.1, 0.2), Goa, Daman & Diu (0.1 pp; 95% CI − 0.2, 0.4), and Kerala (0.2 pp; 95% CI 0.0, 0.3).

The prevalence of stunting declined in all states, although not uniformly everywhere (Fig. 2 and Supplementary Table S4). The AAR for stunting was the greatest in Himachal Pradesh (1.5 pp; 95% CI 1.2, 1.8) and Tripura (1.4 pp; 95% CI 1.1, 1.7), and the lowest in Manipur (0.4 pp; 95% CI 0.1, 0.6), and Rajasthan (0.4 pp; 95% CI 0.3, 0.6).

Wasting declined in half of the states, although the 95% CI contained zero AAR in 13 states (Fig. 3 and Supplementary Table S5). For wasting, the AAR was the greatest in Bihar & Jharkhand (0.4 pp; 95% CI 0.2, 0.5) and Orissa (0.4 pp; 95% CI 0.2, 0.5). Thirteen states had an increase in wasting (statistically significant in five); with the greatest increase observed in Haryana (− 0.7 pp; 95% − 0.8, − 0.5) and Sikkim (− 0.6 pp; 95% CI − 0.9, − 0.3).

### State-specific trends in inequality in anthropometric outcomes by living standards

Delhi had the greatest decline in the poor–rich gap in the prevalence of underweight between 1993 and 2016 (− 18 pp, 95% CI − 37, 1), although the 95% CI contained zero change (Fig. 4 and Supplementary Table S6). The states with the second and third greatest declines were Haryana (− 16 pp, 95% CI − 27, − 5) and Karnataka (− 15 pp, − 27, − 3). Rajasthan had the largest increase in the poor–rich gap in 2016 (22 pp, 95% CI 12, 33) followed by Arunachal Pradesh—although the 95% CI contains zero (15 pp, 95% CI − 4, 34)—Uttar Pradesh & Uttaranchal (12 pp, 95% CI 5, 19), and Bihar & Jharkhand (9 pp, 95% CI 1, 18). A clear pattern can be observed where states with the lowest poor–rich gap in 1993 had a lower decline (and a greater increase in some cases) in the poor–rich gap in 2016.

The biggest decline in the disadvantage of the poorest children in terms of stunting was observed in Kerala (− 20 pp, 95% CI − 33, 8) and West Bengal (− 20 pp, 95% CI − 31, 8). The biggest increase was in Arunachal Pradesh (28 pp, 95% CI 9, 47) and Uttar Pradesh & Uttaranchal (18 pp, 95% CI 12, 25). Note, however, that in Arunachal Pradesh, the poor had an advantage in stunting, although the 95% CI contained zero poor–rich gap. A pattern can be observed where states with the lowest poor–rich gap in 1993 had a lower decline (and in some cases greater increase) in the poor–rich gap in 2016.

A total of 13 of 26 states had a 95% CI containing zero difference in wasting between richest and poorest in 1993. Himachal Pradesh, Jammu & Kashmir, and Tripura had the biggest decline in the poor–rich gap while Rajasthan had the biggest increase.

### Results from the supplementary analyses

There was a moderate positive correlation between the prevalence of underweight (r = 0.64; Supplementary Fig. S1), stunting (r = 0.48; Supplementary Fig. S2), and wasting (r = 0.62; Supplementary Fig. S3) in the oldest survey, and the AAR in the respective prevalence measures between the oldest and newest survey. There was a strong negative correlation between the change in the poor–rich gap between 1993 and 2016 and the poor–rich gap in 1993 for all measures, particularly stunting (r = − 0.9) (Supplementary Figs. S4–S6).

The poor–rich prevalence ratio for underweight showed an increased poor-disadvantage between 1993 and 2006, which decreased again in 2016, although it was greater than in 1993 (Supplementary Fig. S13). There was a uniform increase in the poor–rich prevalence ratio for stunting between the richest and the poorest children in each survey in India overall, primarily occurring between 1993 and 1999 (Supplementary Fig. S14). The poor–rich prevalence ratio for wasting increased between 1993 and 1999 and decreased between 1999, 2006, and 2016 (Supplementary Fig. S15).

The Erreygers concentration index (Supplementary Figs. S19–S21) shows similar patterns as the poor–rich prevalence gap for India overall (Figs. 4, 5 and 6). The modified concentration index (Supplementary Figs. S22–S24) shows similar patterns as the poor–rich prevalence ratios (Supplementary Figs. S13–S15).

## Discussion

This study had two salient findings. First, underweight and stunting prevalence decreased in all states between 1993 and 2016, although the decrease was not statistically significant in three states for underweight. Second, the poor–rich prevalence gap for underweight and stunting decreased in 15 out of 26 states for both outcomes: however, the decline was only statistically significant in five states for both outcomes. In three and four states did the poor–rich prevalence gap in underweight and stunting, respectively, increase statistically significantly. Prevalence and inequality for wasting remained similar in India overall. The change in the poor–rich gap in the prevalence of wasting statistically significantly decreased in two states and increased in one state. Concentration indices measuring disparities across the whole distribution of living standards support the overall pattern.

This study has limitations. First, several states did not record child height or weight in 1993, so the composition of our pooled estimates varies between 1993 and the other survey years. This study was, however, focused on state-level analysis. Second, data quality issues in the NFHS have been highlighted, particularly regarding incorrect age in older surveys^22^. Further, small sample sizes lead to imprecise estimates in the older surveys. Small sample sizes and data quality issues in older surveys should be kept in mind when interpreting this paper's findings. The NFHS is, however, the most reliable data collected on child anthropometry spanning over two decades^17^.

West Bengal had the fastest decline in underweight, 1.1 pp decline per year on average, and Himachal Pradesh had the fastest decline for stunting or 1.5 pp decline per year on average. Haryana, Goa, Daman & Diu, and Kerala had the slowest decline for underweight, which were also not statistically significant. Manipur had the slowest decline for stunting, 0.35 pp decline per year on average. Bihar & Jharkhand had the fastest decline in the prevalence of wasting, 0.36 pp per year on average, while Haryana had the biggest increase. Our supplementary analysis showed that, in general, states with a higher prevalence in 1993 had moderately faster declines for both underweight and stunting, leading to some convergence between states in the prevalence between 1993 and 2016.

The largest decrease in the poor–rich gap for underweight was observed in Delhi, Haryana, Karnataka, Punjab, and Tamil Nadu (although the 95% CI for Delhi contained zero change). Rajasthan, Arunachal Pradesh, Uttar Pradesh & Uttaranchal, and Bihar & Jharkhand had the greatest increase in the poor–rich gap (although the 95% CI for Arunachal Pradesh contained zero change). Kerala, West Bengal, Tripura, Karnataka, Tamil Nadu, Goa, Daman & Diu, Delhi, Jammu & Kashmir, and Madhya Pradesh & Chhattisgarh had the greatest decline in the poor–rich prevalence gap for stunting (although the increase was not statistically significant for Tripura, Goa, Daman & Diu, Delhi, and Jammu & Kashmir). Arunachal Pradesh, Uttar Pradesh & Uttaranchal, Rajasthan, Meghalaya, and Bihar & Jharkhand had the biggest increase in the poor–rich gap for stunting (although the increase was not statistically significant for Meghalaya). Arunachal Pradesh did, however, show a non-statistically significant poor-advantage in stunting in 1993. The change in the poor–rich gap in wasting showed a statistically significant decrease in two states, Himachal Pradesh and Jammu & Kashmir, and an increase in one, Rajasthan. A clear pattern emerged, particularly for underweight and stunting, where the poor–rich gap in the prevalence declined more in states where the poor–rich gap was greater in 1993.

The prevalence and AAR patterns of the three measures indicate that chronic nutrition has declined more substantially. However, there may also be some differences in how well these measures reflect undernutrition faced by children: when acute undernutrition is measured using mid-upper arm circumference, less than half as many children were found to be acutely undernourished in India as when wasting was used, which suggests the prevalence of wasting may overestimate acute undernutrition in India^21^. We are unaware of any estimates for trends in mid-upper arm circumference since the 1990s in India, so it is unclear how trends in acute undernutrition would differ using mid-upper arm circumference. Further, this paper did not study overweight and obesity in childhood, which other studies have found to be increasing in India^23^.

Our supplementary analysis shows that the relative burden of stunting and underweight increased for the poorest children, compared to the richest, between 1993 and 2016 in India overall. This increase is in accordance with a previous study that found an increase in the relative burden of stunting among the poor, measured as a prevalence ratio, between 1993 and 2006 NFHS, in India overall^7^. This trend continued until 2016, according to our study. The same study also found an increase in the percentage point difference between the poorest and the richest until 2006. We do, however, find that the percentage point difference then declined slightly between 2006 and 2016. Although we find evidence of moderate convergence between states over the whole period, our results are similar to that of another paper, which found little evidence of convergence between 2006 and 2016^24^. The decline in poor anthropometric outcomes is in accordance with improvements in other measures of living standards such as increasing GDP and declining neonatal and infant mortality rate^25^. The increased inequality in stunting by living standard (especially when considering the relative burden of stunting) may reflect increased economic inequality in India as measured by the GINI coefficient^25^.

We further find that the poor–rich prevalence ratio had increased in 14 states for underweight and 20 states for stunting (and the increase was statistically significant in 8 states for both), while there was only a statistically significant decline in the prevalence ratio in one state for underweight and two states for stunting. The observations that the poor–rich prevalence gap remained similar while the poor–rich prevalence ratio showed a considerable increase in disparities for India overall indicates that the decline in prevalence has been somewhat parallel across wealth groups, rather than being pro-poor.

Compared to sub-Saharan Africa, India had a much faster reduction in the prevalence of underweight, although India started from a much higher level. In sub-Saharan Africa in 1990, 29% of children were underweight, and with an AAR of 0.36 pp, that share decreased to 20%. South-Eastern Asia started at a lower level in 1990, with 31% of children being underweight, and with an AAR of 0.6 pp, that share decreased to 16%, which is the same AAR as for India overall, in our study^26^.

Pan-India declines in underweight and stunting levels since 1993 point towards better access to food and nutrition, medical care, water, and sanitation, expectedly due to government programs and rising family incomes. However, increasing inequality among large states such as Uttar Pradesh & Uttaranchal, particularly for stunting, calls for a more focused approach to developing public health and nutritional interventions. These initiatives appear to be insufficient as child undernutrition remains high in most states. Further, these initiatives have not successfully brought down the level of inequality in anthropometric measures by household living standards, which has remained similar in India overall. This paper helps identify states where levels and inequality in anthropometric outcomes remain particularly high, and progress has been slow, so policymakers can target states for poverty reduction and improve nutrition overall. A more detailed analysis of the influence of ICDS benefits and other programs on anthropometric outcomes and reducing inequities is required.

## Supplementary Information

Supplementary Information.

## Data Availability

DHS data are available at https://dhsprogram.com (requiring a simple application).
