# Supplementary material for: Trends in underweight, stunting, and wasting prevalence and inequality among children under three in Indian states, 1993–2016
Source: Sci Rep. 2021 Jul 8;11:14137. doi: 10.1038/s41598-021-93493-1 (PMC8266817; doi:10.1038/s41598-021-93493-1)
Supplement: Supplementary file 1 — Supplementary Information. [file 41598_2021_93493_MOESM1_ESM.pdf]

Supplement

Trends in underweight, stunting, and wasting prevalence and inequality among children under three in Indian states, 1993–2016

Authors

Omar Karlsson, Ph.D.[1,2,*] (okarlsson@hsph.harvard.edu)
Rockli Kim, Sc.D.[3,4] (rocklikim@korea.ac.kr)
Rakesh Sarwal, DrPH[5] (sarwalr@gmail.com)
KS James, Ph.D.[6] (ksjames@gmail.com)
S V Subramanian, Ph.D.[4,7,8] (svsubram@hsph.harvard.edu)

Affiliations

[1] Takemi Program in International Health, Harvard T.H. Chan School of Public Health, Harvard University, 677 Huntington Avenue, Boston MA 02115, United States
[2] Department of Economic History, School of Economics and Management, Lund University, P.O. Box 7083, 220 07 Lund, Sweden
[3] Division of Health Policy & Management, College of Health Science, Korea University, 145 Anam-ro, Seongbuk-gu, Seoul 02841, Korea
[4] Harvard Center for Population and Development Studies, 9 Bow Street, Cambridge MA 02138, United States
[5] National Institution for Transforming India (NITI) Aayog, Government of India, New Delhi 110001, India
[6] International Institute for Population Sciences (IIPS), Deonar, Mumbai -400088 India
[7] Department of Social and Behavioral Sciences, Harvard T.H. Chan School of Public Health, Harvard University, 677 Huntington Avenue, Boston MA 02115, United States
[8] Honorary Senior Fellow (Non-Resident), National Institution for Transforming India (NITI) Aayog, Government of India

Figure S1. State-level average annual reduction (AAR) in the prevalence of underweight between 1993* and 2016 plotted against prevalence of underweight in 1993* 4

Figure S2. State-level average annual reduction (AAR) in the prevalence of stunting between 1993* and 2016 plotted against prevalence of stunting in 1993* 5

Figure S3. State-level average annual reduction (AAR) in the prevalence of wasting between 1993* and 2016 plotted against prevalence of wasting in 1993* 6

Figure S4. State-level poor-rich prevalence gap in underweight in 1993* plotted against change in the poor-rich gap in underweight between 1993* and 2016 7

Figure S5. State-level poor-rich prevalence gap in stunting in 1993* plotted against change in the poor-rich gap in stunting between 1993* and 2016 8

Figure S6. State-level poor-rich prevalence gap in wasting in 1993* plotted against change in the poor-rich gap in wasting between 1993* and 2016 9

Figure S7. Prevalence of underweight and average annual rate of reduction (AARR) in underweight between 1993 and 2016 10

Figure S8. Prevalence of stunting and average annual rate of reduction (AARR) in stunting between 1993 and 2016 11

Figure S9. Prevalence of wasting and average annual rate of reduction (AARR) in wasting between 1993 and 2016 12

Figure S10. State-level average annual rate of reduction (AARR) in the prevalence of underweight between 1993* and 2016 plotted against prevalence of underweight in 1993* 13

Figure S11. State-level average annual rate of reduction (AARR) in the prevalence of stunting between 1993* and 2016 plotted against prevalence of stunting in 1993* 14

Figure S12. State-level average annual rate of reduction (AARR) in the prevalence of wasting between 1993* and 2016 plotted against prevalence of wasting in 1993* 15

Figure S13. Changes in the poor-rich ratio in prevalence of underweight 16

Figure S14. Changes in the poor-rich ratio in prevalence of stunting 17

Figure S15. Changes in the poor-rich ratio in prevalence of wasting 18

Figure S16. State-level poor-rich prevalence ratio in underweight in 1993* plotted against 19

Figure S17. State-level poor-rich prevalence ratio in stunting in 1993* plotted against relative change in the poor-rich ratio in stunting between 1993* and 2016 20

Figure S18. State-level poor-rich prevalence ratio in wasting in 1993* plotted against relative change in the poor-rich ratio in wasting between 1993* and 2016 21

Figure S19. Erreygers concentration index for underweight by household wealth 22

Figure S20. Erreygers concentration index for stunting by household wealth 23

Figure S21. Erreygers concentration index for wasting by household wealth 24

Figure S22. Modified concentration index for underweight by household wealth 25

Figure S23. Modified concentration index for stunting by household wealth 26

Figure S24. Modified concentration index for wasting by household wealth 27

Figure S25. Prevalence of underweight and average annual reduction (AAR) in underweight between 1993 and 2016, adjusted for month of interview 28

Figure S26. Prevalence of stunting and average annual reduction (AAR) in stunting between 1993 and 2016, adjusted for month of interview 29

Figure S27. Prevalence of wasting and average annual reduction (AAR) in wasting between 1993 and 2016, adjusted for month of interview 30

Figure S28. Prevalence of underweight and average annual reduction (AAR) in underweight between 1993 and 2016, males 31

Figure S29. Prevalence of stunting and average annual reduction (AAR) in stunting between 1993 and 2016, males 32

Figure S30. Prevalence of wasting and average annual reduction (AAR) in wasting between 1993 and 2016, males 33

Figure S31. Prevalence of underweight and average annual reduction (AAR) in underweight between 1993 and 2016, females 34

Figure S32. Prevalence of stunting and average annual reduction (AAR) in stunting between 1993 and 2016, females 35

Figure S33. Prevalence of wasting and average annual reduction (AAR) in wasting between 1993 and 2016, females.. 36
Figure S34. Changes in the poor-rich gap in prevalence of underweight, males.. 37
Figure S35. Changes in the poor-rich gap in prevalence of stunting, males 38
Figure S36. Changes in the poor-rich gap in prevalence of wasting, males.. 39
Figure S37. Changes in the poor-rich gap in prevalence of underweight, females 40
Figure S38. Changes in the poor-rich gap in prevalence of stunting, females 41
Figure S39. Changes in the poor-rich gap in prevalence of wasting, females... 42
Figure S40. Prevalence of severe underweight and average annual reduction (AAR) in severe underweight between 1993 and 2016 43
Figure S41. Prevalence of severe stunting and average annual reduction (AAR) in severe stunting between 1993 and 2016 44
Figure S42. Prevalence of severe wasting and average annual reduction (AAR) in severe wasting between 1993 and 2016 45
Figure S43. Changes in the poor-rich gap in prevalence of severe underweight.. 46
Figure S44. Changes in the poor-rich gap in prevalence of severe stunting... 47
Figure S45. Changes in the poor-rich gap in prevalence of severe wasting 48
Figure S46. Mean weight-for-age and average annual reduction (AAR) in weight-for-age between 1993 and 2016 49
Figure S47. Mean height-for-age and average annual reduction (AAR) in height-for-age between 1993 and 2016... 50
Figure S48. Mean weight-for-height and average annual reduction (AAR) in weight-for-height between 1993 and 2016 51
Figure S49. Changes in the poor-rich gap in mean weight-for-age.. 52
Figure S50. Changes in the poor-rich gap in mean height-for-age... 53
Figure S51. Changes in the poor-rich gap in mean weight-for-height 54
Table S1. Full sample (of children 0–36 months) and analytical samples for stunting, wasting and underweight, by state and year 55
Table S2. Comparing household wealth between included children and children with missing underweight, stunting, or wasting, by state and year 57
Table S3. Prevalence of underweight and average annual reduction (AAR) 60
Table S4. Prevalence of stunting and average annual reduction (AAR) 61
Table S5. Prevalence of wasting and average annual reduction (AAR).. 62
Table S6. Poor-rich gap in 1993 and change in poor-rich gap between 1993 and 2016.. 63

Figure S1. State-level average annual reduction (AAR) in the prevalence of underweight between 1993* and 2016 plotted against prevalence of underweight in 1993*

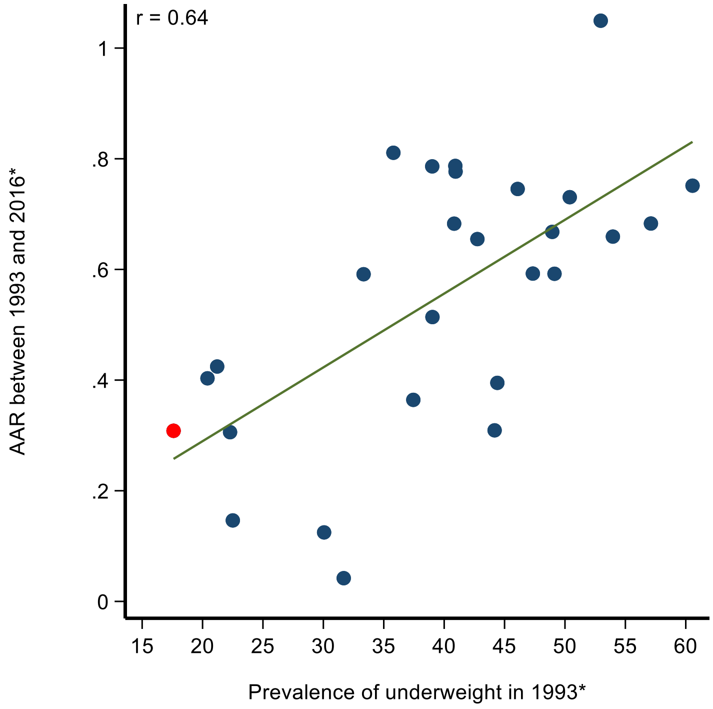

Notes: *red dots indicate states with no data for 1993, using 1999 instead. Pearson's correlation coefficient is shown.

Figure S2. State-level average annual reduction (AAR) in the prevalence of stunting between 1993* and 2016 plotted against prevalence of stunting in 1993*

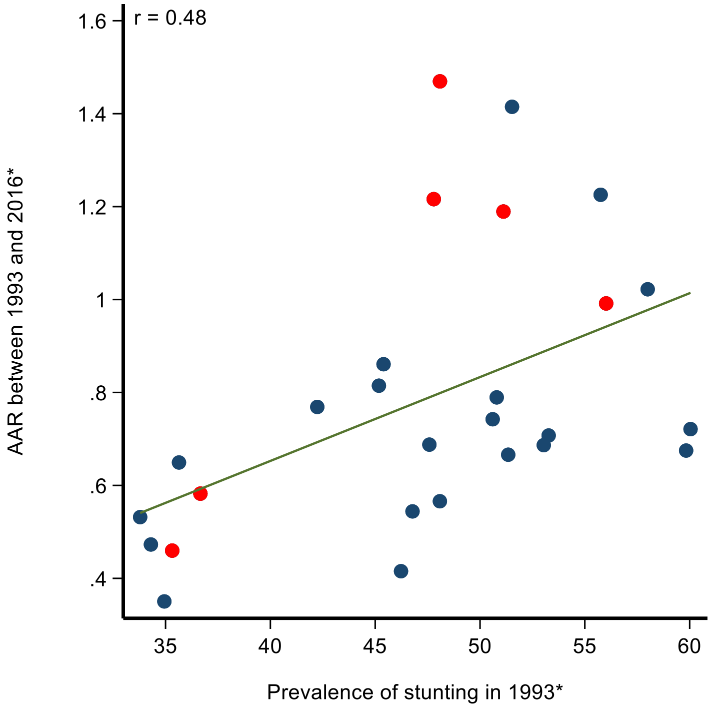

Notes: *red dots indicate states with no data for 1993, using 1999 instead. Pearson's correlation coefficient is shown.

Figure S3. State-level average annual reduction (AAR) in the prevalence of wasting between 1993* and 2016 plotted against prevalence of wasting in 1993*

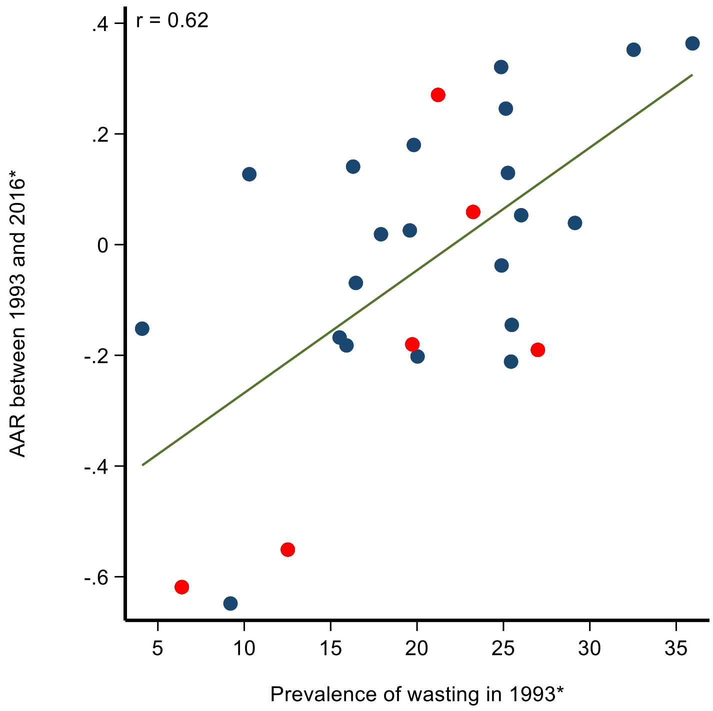

Notes: *red dots indicate states with no data for 1993, using 1999 instead. Pearson's correlation coefficient is shown.

Figure S4. State-level poor-rich prevalence gap in underweight in 1993* plotted against change in the poor-rich gap in underweight between 1993* and 2016

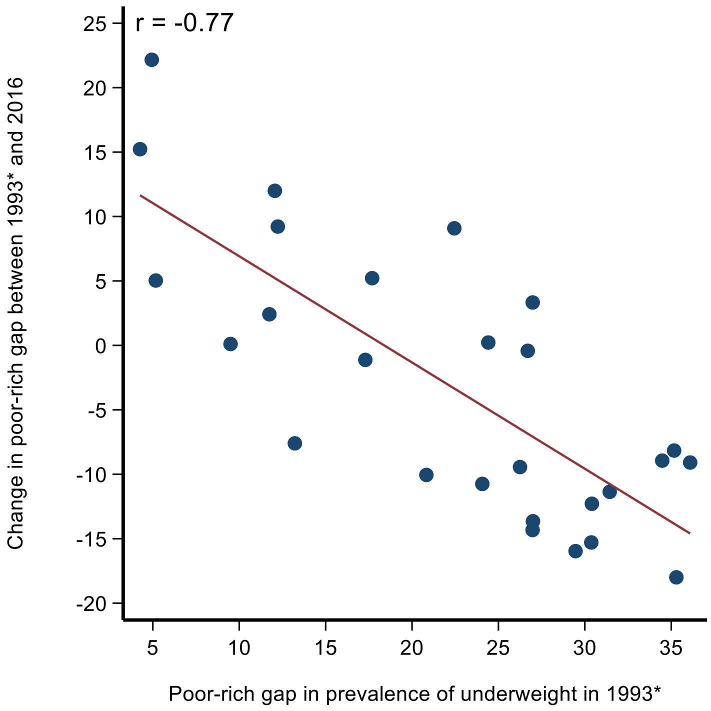

Notes: *A few states had no data in 1993 and 1999 was used. Pearson's correlation coefficient is shown.

Figure S5. State-level poor-rich prevalence gap in stunting in 1993* plotted against change in the poor-rich gap in stunting between 1993* and 2016

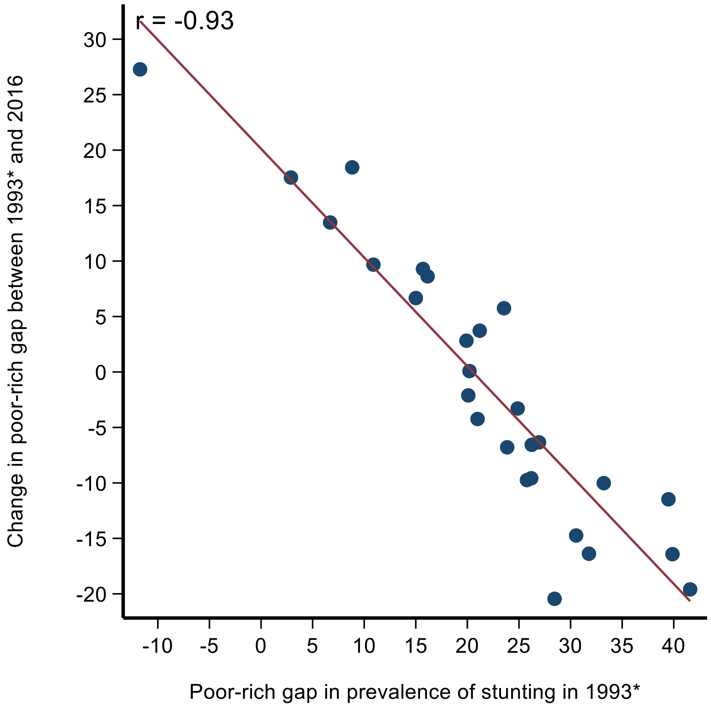

Notes: *A few states had no data in 1993 and 1999 was used. Pearson's correlation coefficient is shown.

Figure S6. State-level poor-rich prevalence gap in wasting in 1993* plotted against change in the poor-rich gap in wasting between 1993* and 2016

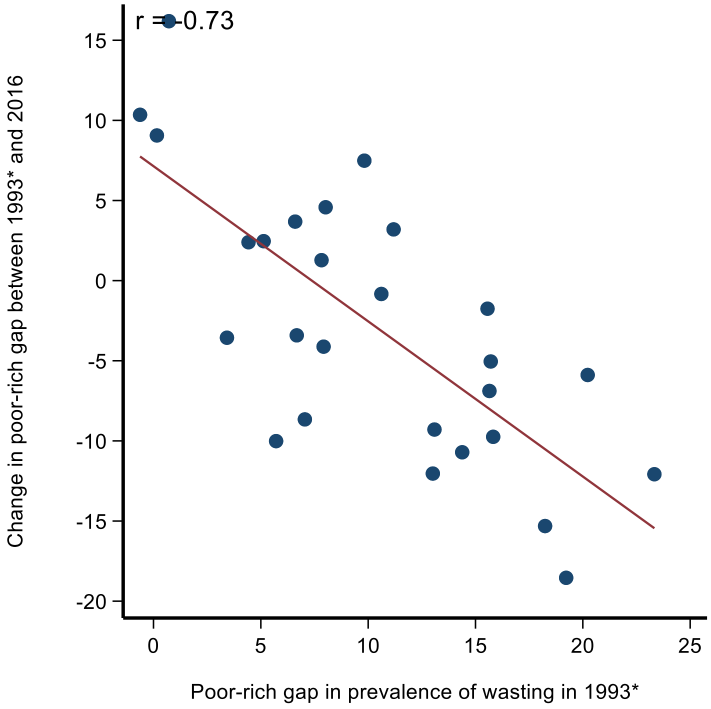

Notes: *A few states had no data in 1993 and 1999 was used. Pearson's correlation coefficient is shown.

Figure S7. Prevalence of underweight and average annual rate of reduction (AARR) in underweight between 1993 and 2016

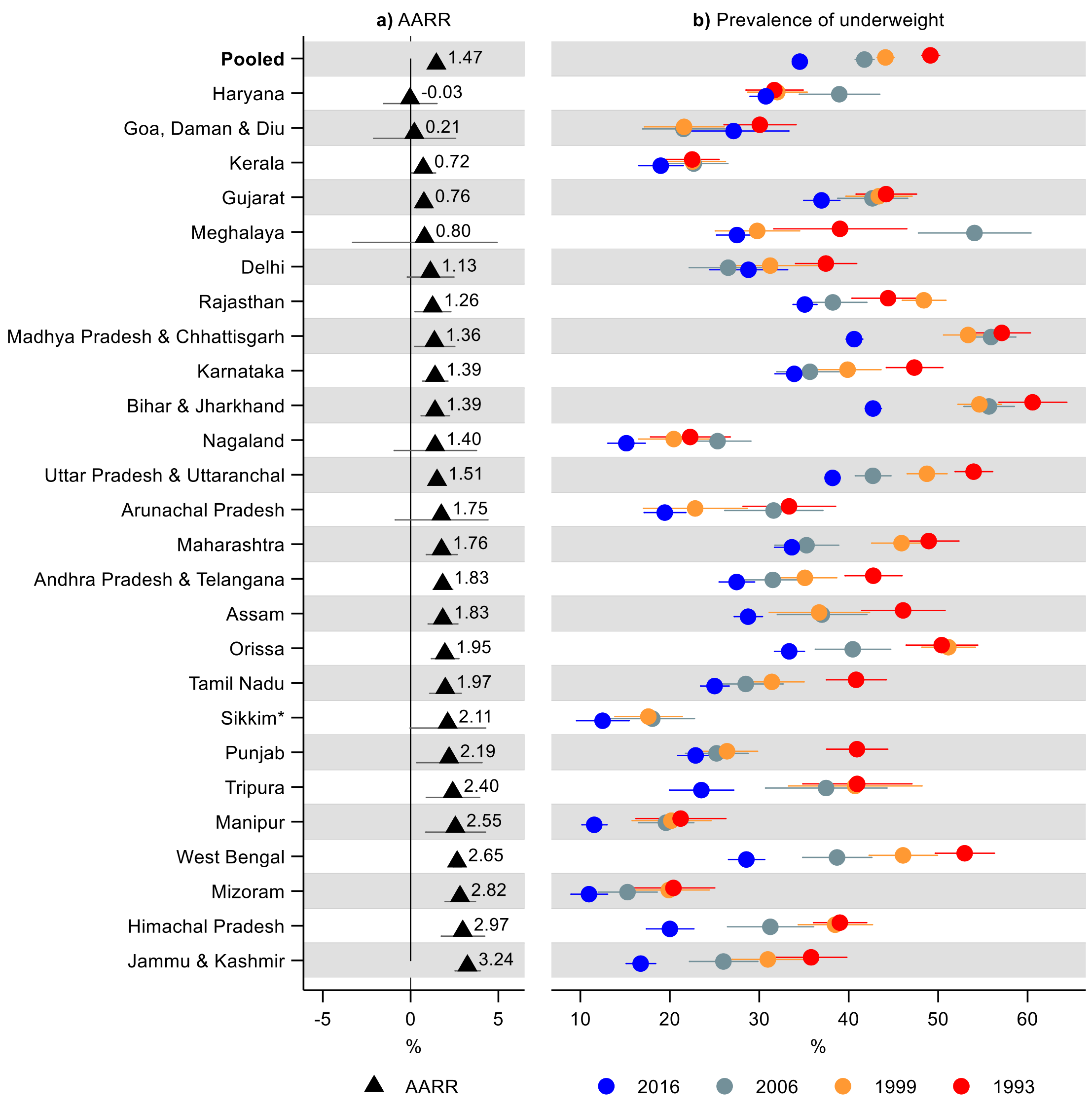

Notes: *Indicates states with no data for 1993: 1999 was used instead. Average annual rate of reduction (AARR) shows average annual percentage reduction in prevalence of underweight in each state. 95% confidence intervals are shown. Estimates are weighted using sampling weights and confidence intervals were adjusted for clustering at the PSU-level.

Figure S8. Prevalence of stunting and average annual rate of reduction (AARR) in stunting between 1993 and 2016

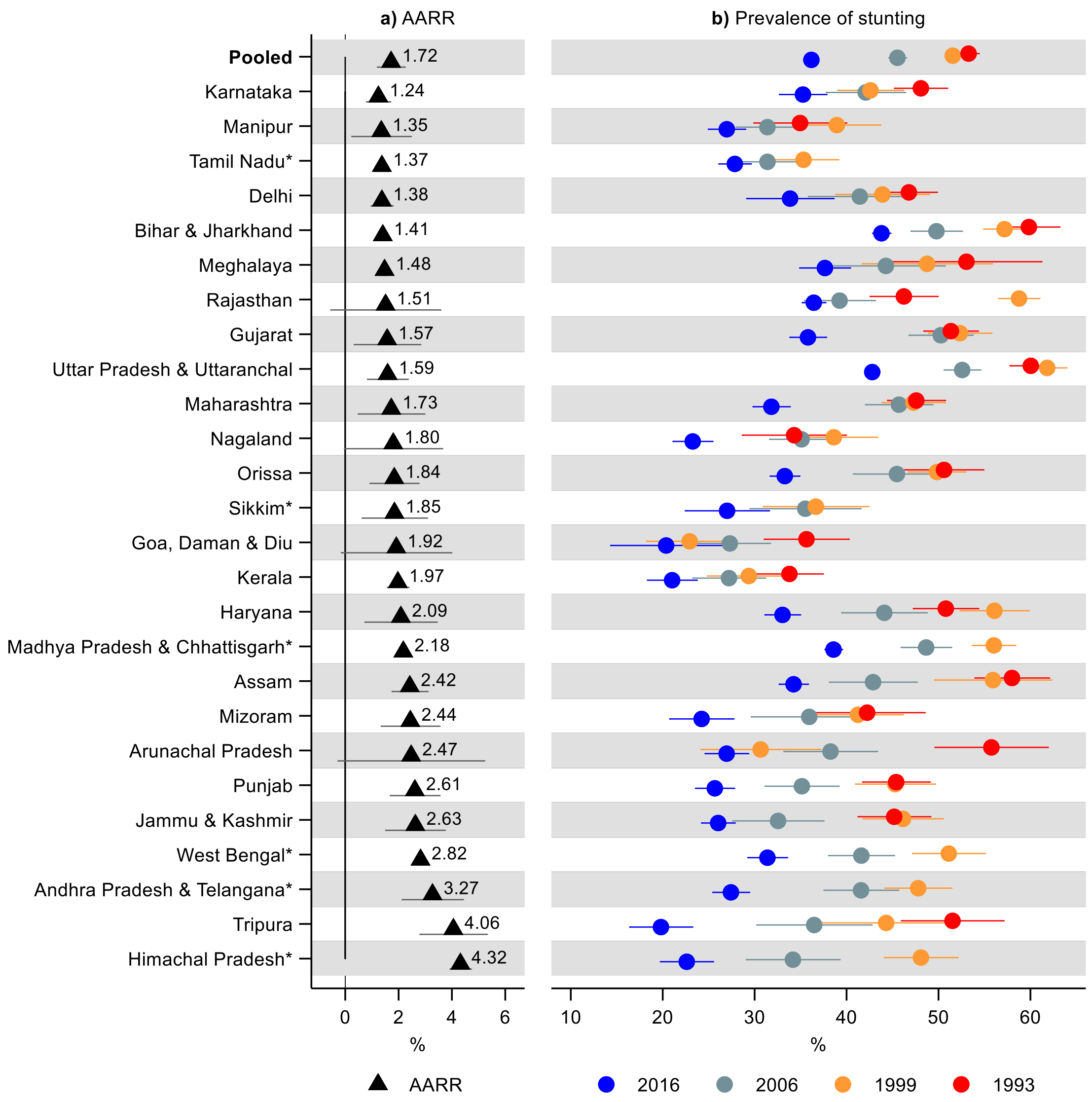

Notes: *Indicates states with no data for 1993: 1999 was used instead. Average annual rate of reduction (AARR) shows average annual percentage reduction in prevalence of stunting in each state. 95% confidence intervals are shown. Estimates are weighted using sampling weights and confidence intervals were adjusted for clustering at the PSU-level.

Figure S9. Prevalence of wasting and average annual rate of reduction (AARR) in wasting between 1993 and 2016

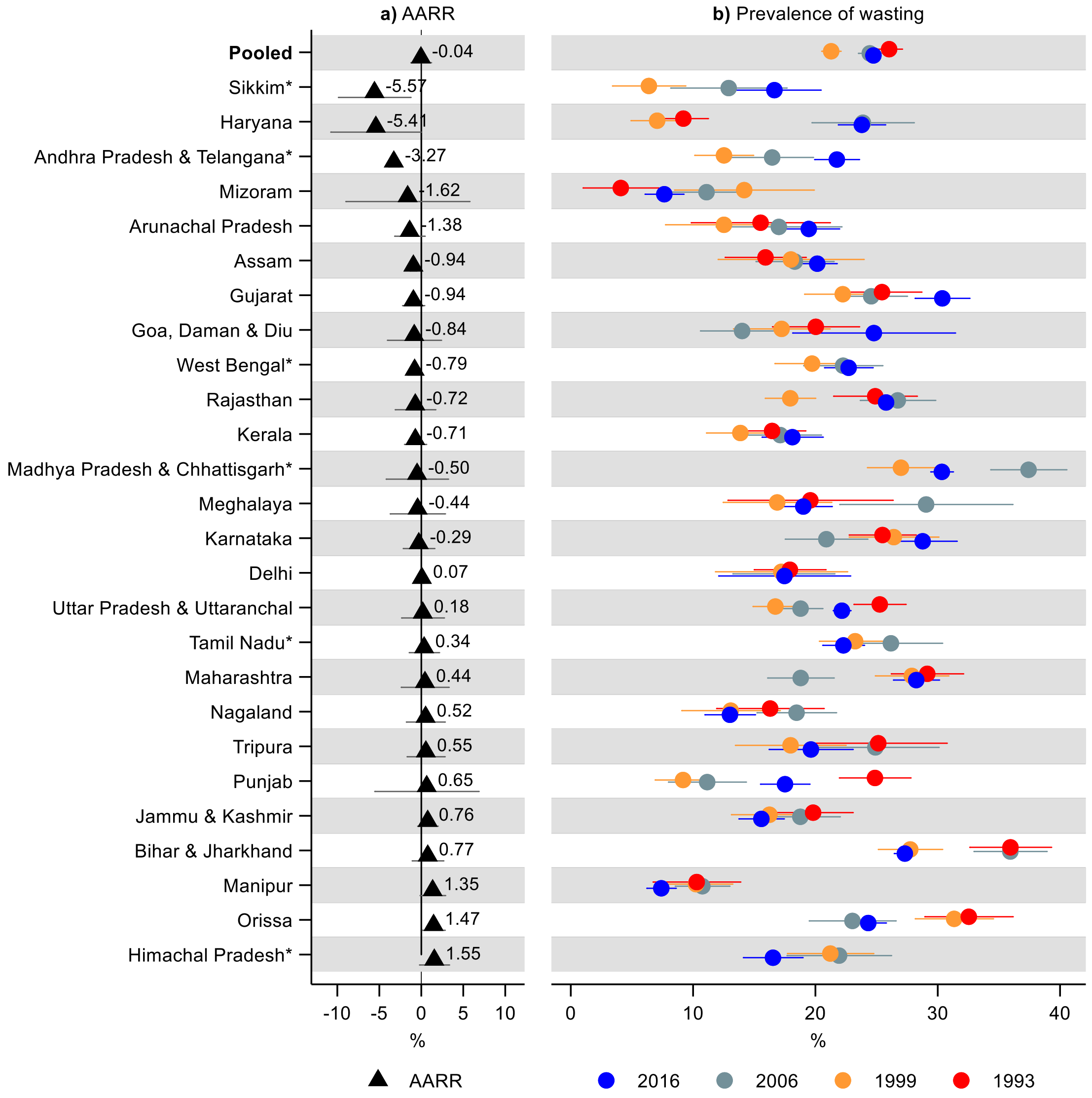

Notes: *Indicates states with no data for 1993: 1999 was used instead. Average annual rate of reduction (AARR) shows average annual percentage reduction in prevalence of wasting in each state. 95% confidence intervals are shown. Estimates are weighted using sampling weights and confidence intervals were adjusted for clustering at the PSU-level.

Figure S10. State-level average annual rate of reduction (AARR) in the prevalence of underweight between 1993* and 2016 plotted against prevalence of underweight in 1993*

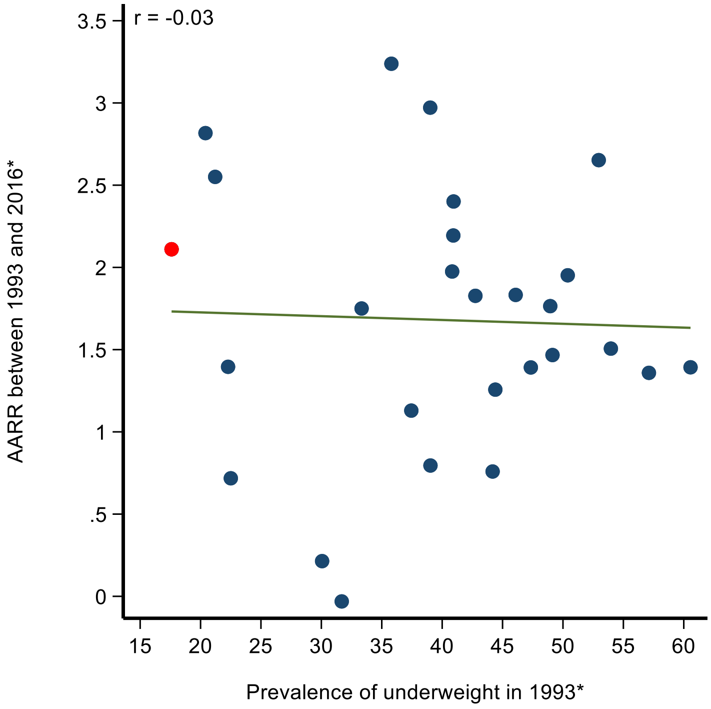

Notes: *red dots indicate states with no data for 1993, using 1999 instead. Pearson's correlation coefficient is shown.

Figure S11. State-level average annual rate of reduction (AARR) in the prevalence of stunting between 1993* and 2016 plotted against prevalence of stunting in 1993*

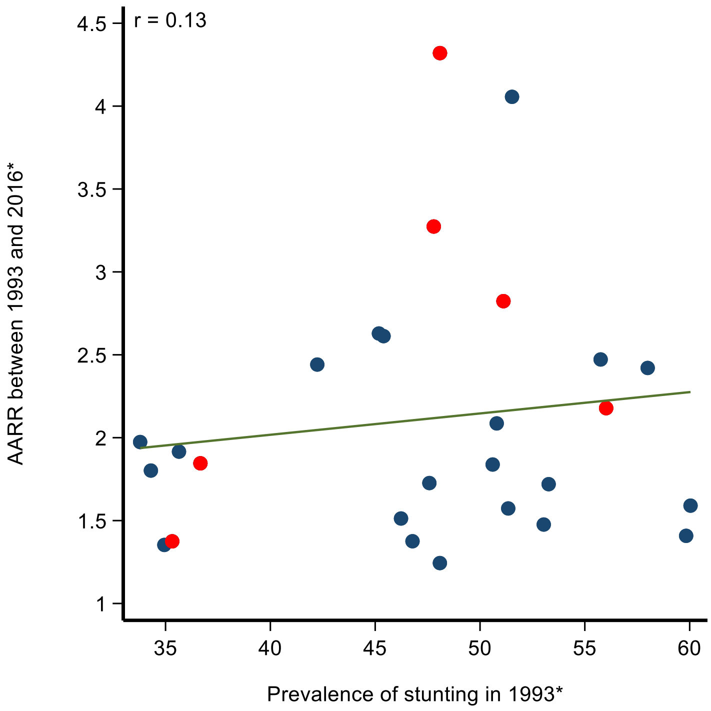

Notes: *red dots indicate states with no data for 1993, using 1999 instead. Pearson's correlation coefficient is shown.

Figure S12. State-level average annual rate of reduction (AARR) in the prevalence of wasting between 1993* and 2016 plotted against prevalence of wasting in 1993*

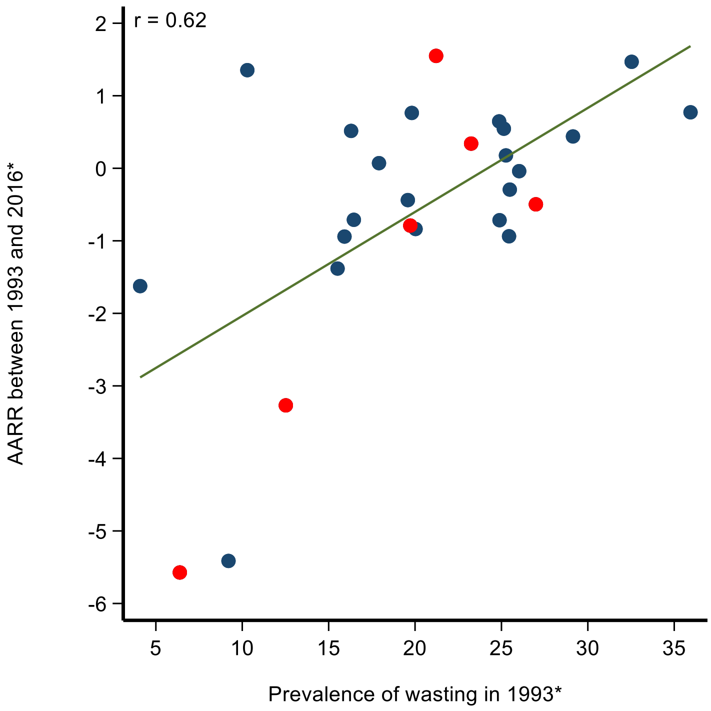

Notes: *red dots indicate states with no data for 1993, using 1999 instead. Pearson's correlation coefficient is shown.

Figure S13. Changes in the poor-rich ratio in prevalence of underweight

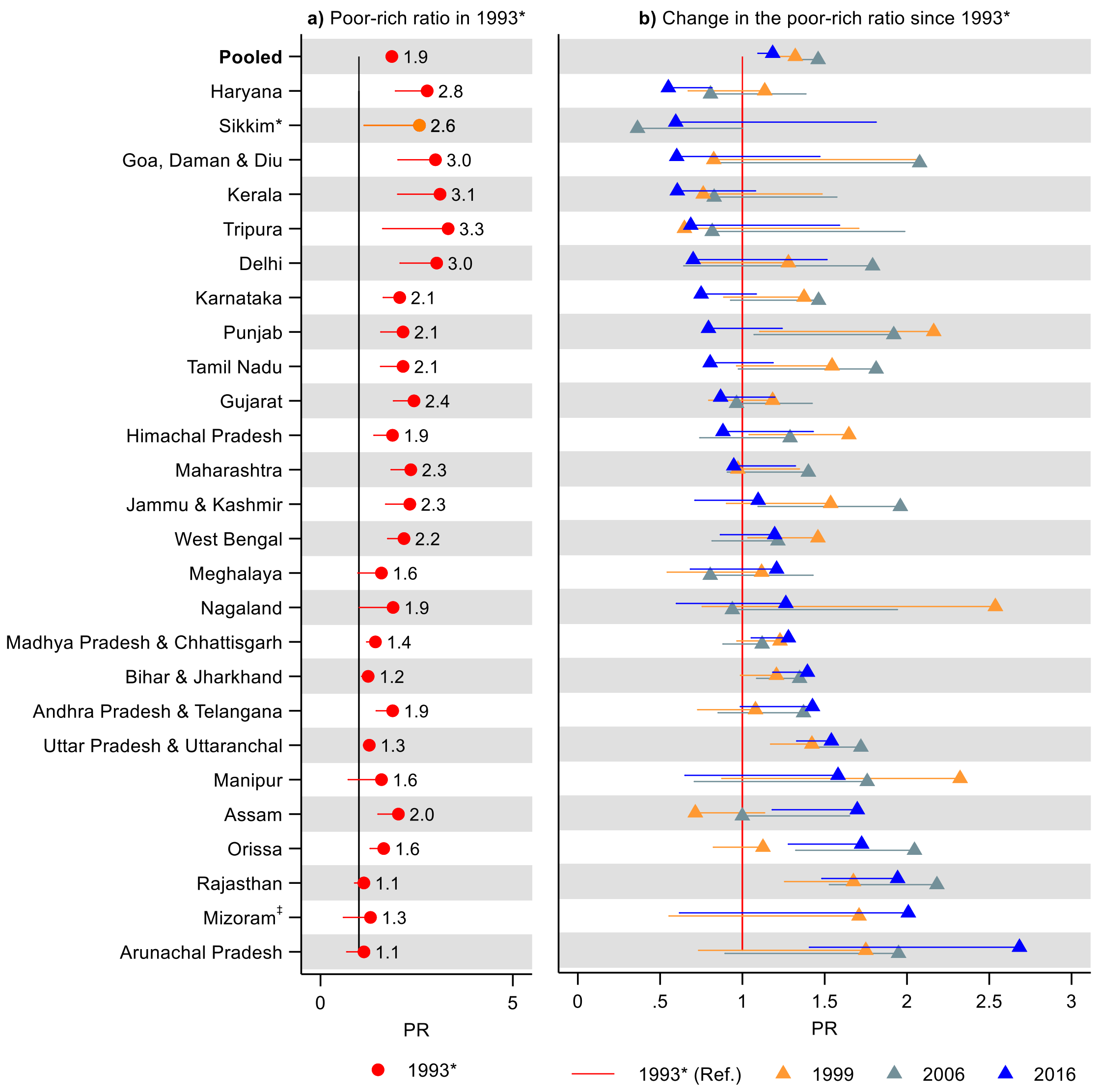

Notes: Prevalence ratios (PR) are shown. In Panel a) an estimate greater than one indicates a disadvantage for the poorest children, compared to the richest children, in 1993. An estimate below one in Panel b) indicates that the poor-rich gap (which usually shows a poor disadvantage in Panel a) has shrunk since 1993. The estimates were obtained from an interaction model (Poisson) for each state: Panel a) shows the terms for the poorest wealth quintile and Panel b) shows the interaction terms (ie, between poorest quintile and year). The terms for year as well as all terms involving quintiles other than the poorest are excluded from the figure. Vertical lines (at 1) indicate no poor-rich difference in Panel a) and no change in rich-poor gap in Panel b). *Indicates states with no data for 1993: 1999 was used instead. ‡Extreme point estimates (more than 3 times the interquartile range from the median) were omitted to improve readability in Panel b). 95% confidence bounds are shown. Only one confidence bound is shown to improve readability: an upper bound where the estimated difference was lower than 1 and a lower bound where the estimated difference was greater than zero. Estimates are weighted using sampling weights and confidence intervals were adjusted for clustering at the PSU-level.

Figure S14. Changes in the poor-rich ratio in prevalence of stunting

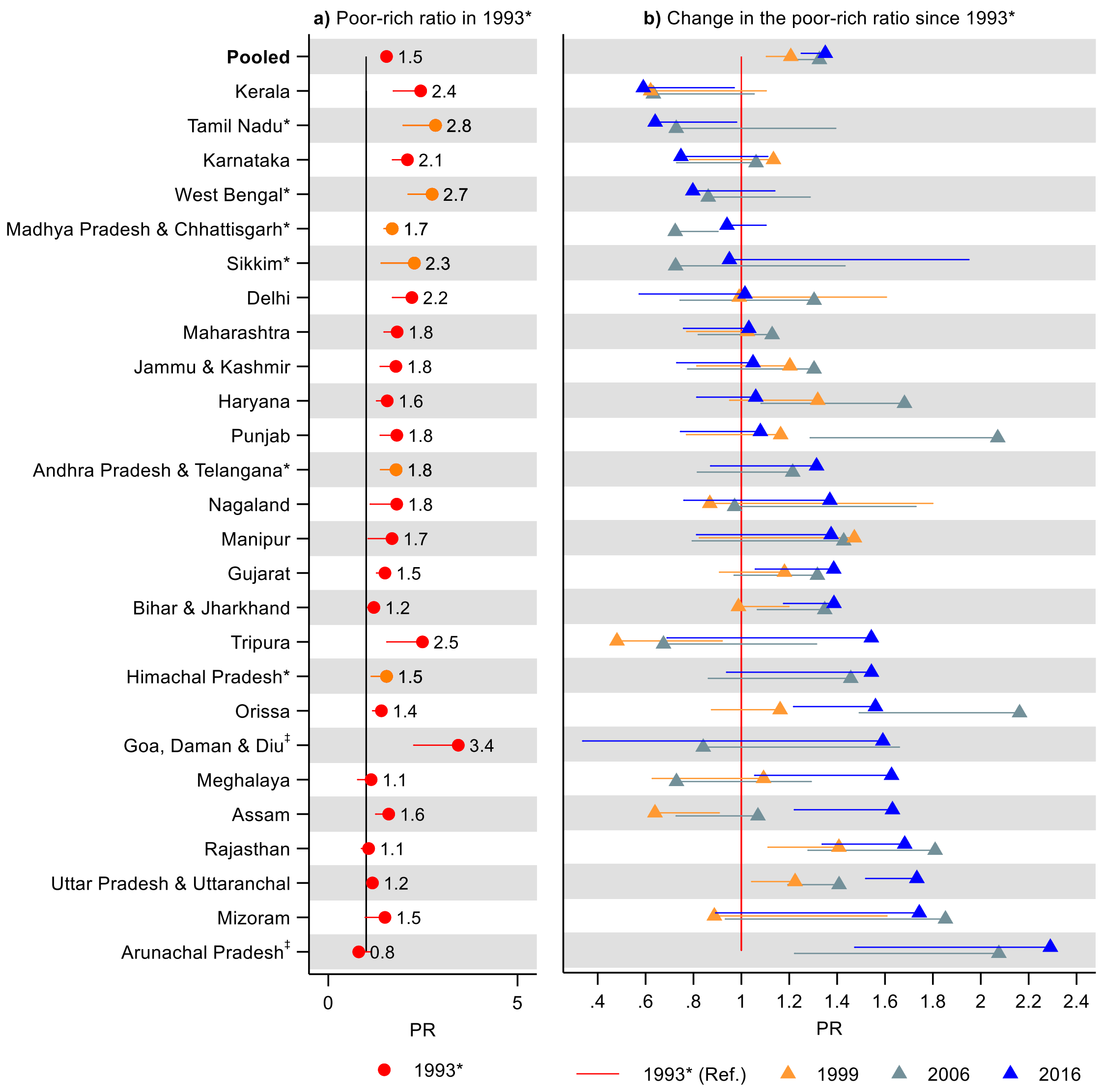

Notes: Prevalence ratios (PR) are shown. In Panel a) an estimate greater than one indicates a disadvantage for the poorest children, compared to the richest children, in 1993. An estimate below one in Panel b) indicates that the poor-rich gap (which usually shows a poor disadvantage in Panel a) has shrunk since 1993. The estimates were obtained from an interaction model (Poisson) for each state: Panel a) shows the terms for the poorest wealth quintile and Panel b) shows the interaction terms (ie, between poorest quintile and year). The terms for year as well as all terms involving quintiles other than the poorest are excluded from the figure. Vertical lines (at 1) indicate no poor-rich difference in Panel a) and no change in rich-poor gap in Panel b). *Indicates states with no data for 1993: 1999 was used instead. ‡Extreme point estimates (more than 3 times the interquartile range from the median) were omitted to improve readability in Panel b). 95% confidence bounds are shown. Only one confidence bound is shown to improve readability: an upper bound where the estimated difference was lower than 1 and a lower bound where the estimated difference was greater than zero. Estimates are weighted using sampling weights and confidence intervals were adjusted for clustering at the PSU-level.

Figure S15. Changes in the poor-rich ratio in prevalence of wasting

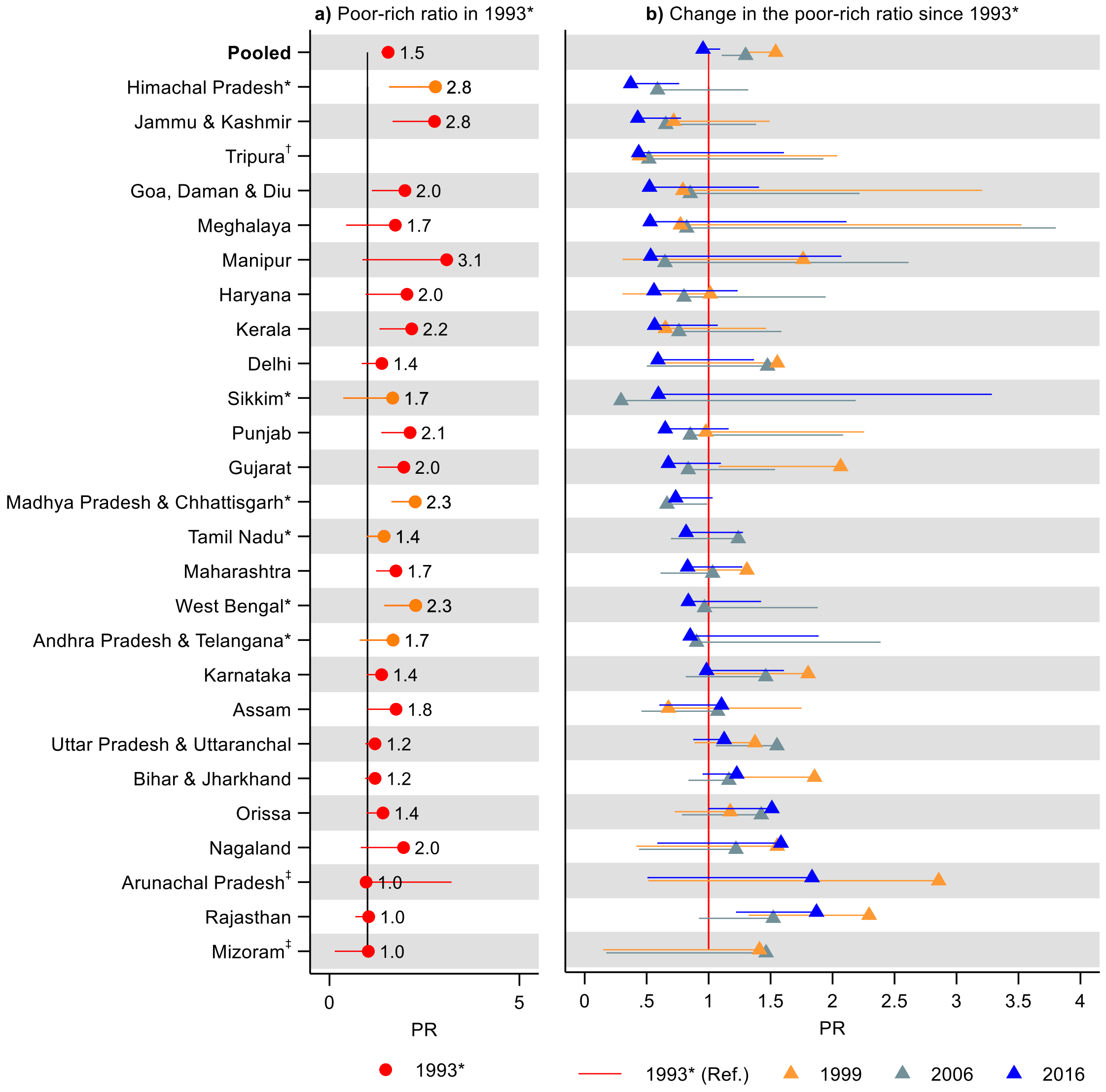

Notes: Prevalence ratios (PR) are shown. In Panel a) an estimate greater than one indicates a disadvantage for the poorest children, compared to the richest children, in 1993. An estimate below one in Panel b) indicates that the poor-rich gap (which usually shows a poor disadvantage in Panel a) has shrunk since 1993. The estimates were obtained from an interaction model (Poisson) for each state: Panel a) shows the terms for the poorest wealth quintile and Panel b) shows the interaction terms (ie, between poorest quintile and year). The terms for year as well as all terms involving quintiles other than the poorest are excluded from the figure. Vertical lines (at 1) indicate no poor-rich difference in Panel a) and no change in rich-poor gap in Panel b). *Indicates states with no data for 1993: 1999 was used instead. †‡Extreme point estimates (more than 3 times the interquartile range from the median) were omitted to improve readability in †Panel a) and/or ‡Panel b). 95% confidence bounds are shown. Only one confidence bound is shown to improve readability: an upper bound where the estimated difference was lower than 1 and a lower bound where the estimated difference was greater than zero. Estimates are weighted using sampling weights and confidence intervals were adjusted for clustering at the PSU-level.

Figure S16. State-level poor-rich prevalence ratio in underweight in 1993* plotted against relative change in the poor-rich ratio in underweight between 1993* and 2016

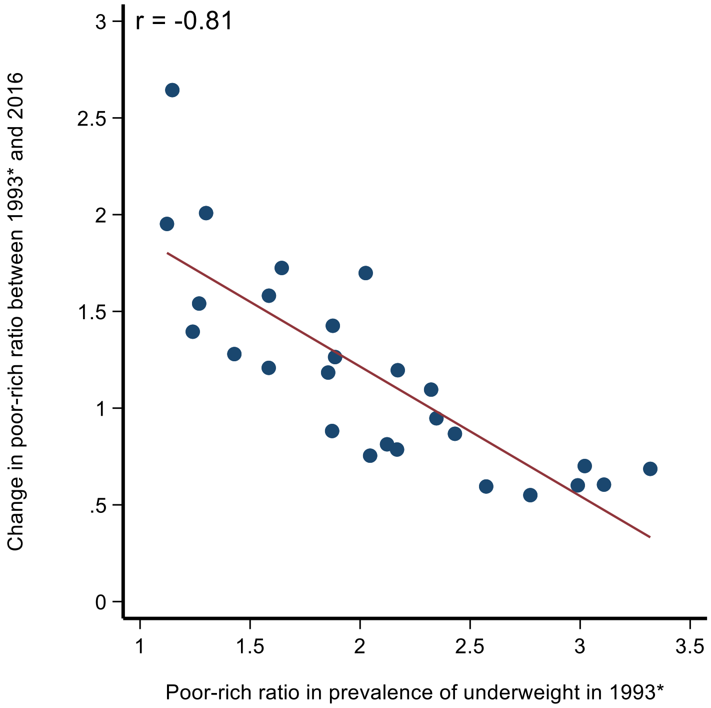

Notes: *A few states had no data in 1993 and 1999 was used. Pearson's correlation coefficient is shown.

Figure S17. State-level poor-rich prevalence ratio in stunting in 1993* plotted against relative change in the poor-rich ratio in stunting between 1993* and 2016

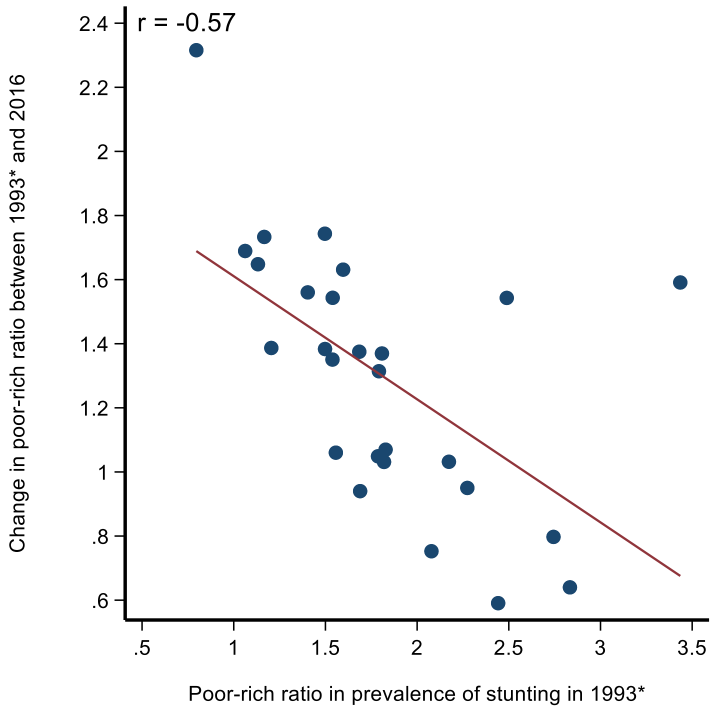

Notes: *A few states had no data in 1993 and 1999 was used. Pearson's correlation coefficient is shown.

Figure S18. State-level poor-rich prevalence ratio in wasting in 1993* plotted against relative change in the poor-rich ratio in wasting between 1993* and 2016

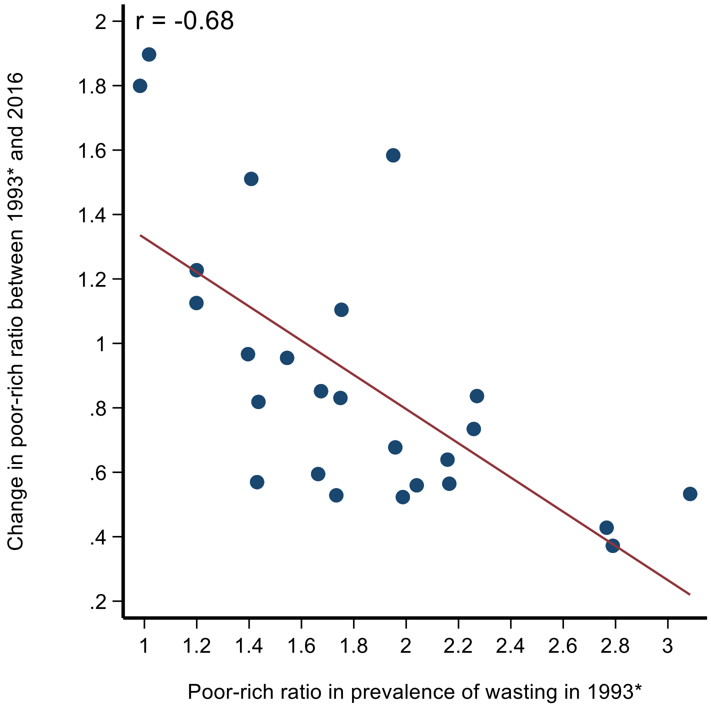

Notes: *A few states had no data in 1993 and 1999 was used. Pearson's correlation coefficient is shown.

Figure S19. Erreygers concentration index for underweight by household wealth

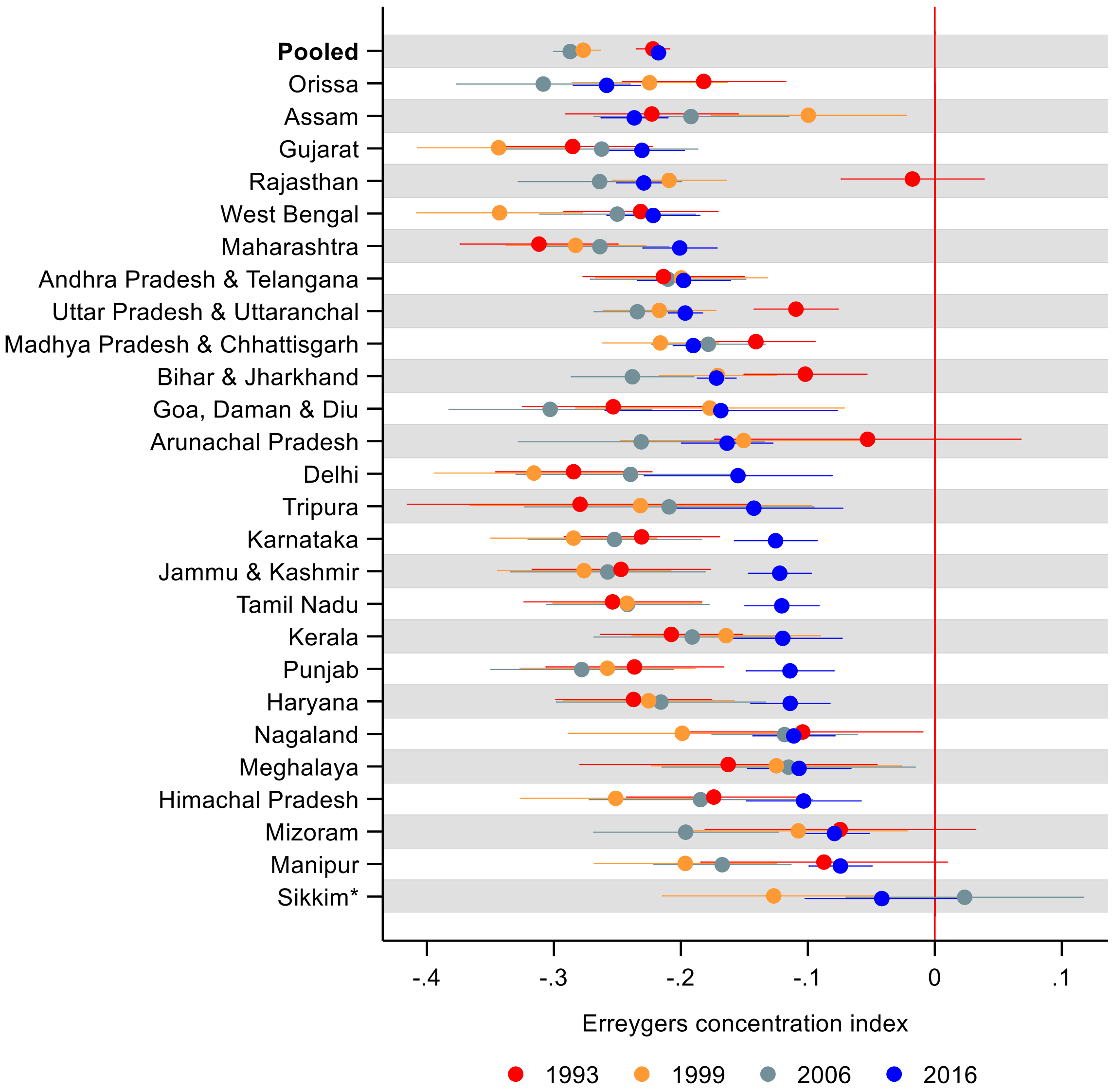

Note: The Erreygers concentration index is bounded with 0–1 limits using a ranking according to state and survey-year specific household wealth index factor score. State-level estimates are ordered from greatest to lowest inequality in 2016. 95% confidence intervals are shown. Estimates are weighted using sampling weights and confidence intervals were adjusted for clustering at the PSU-level.

Figure S20. Erreygers concentration index for stunting by household wealth

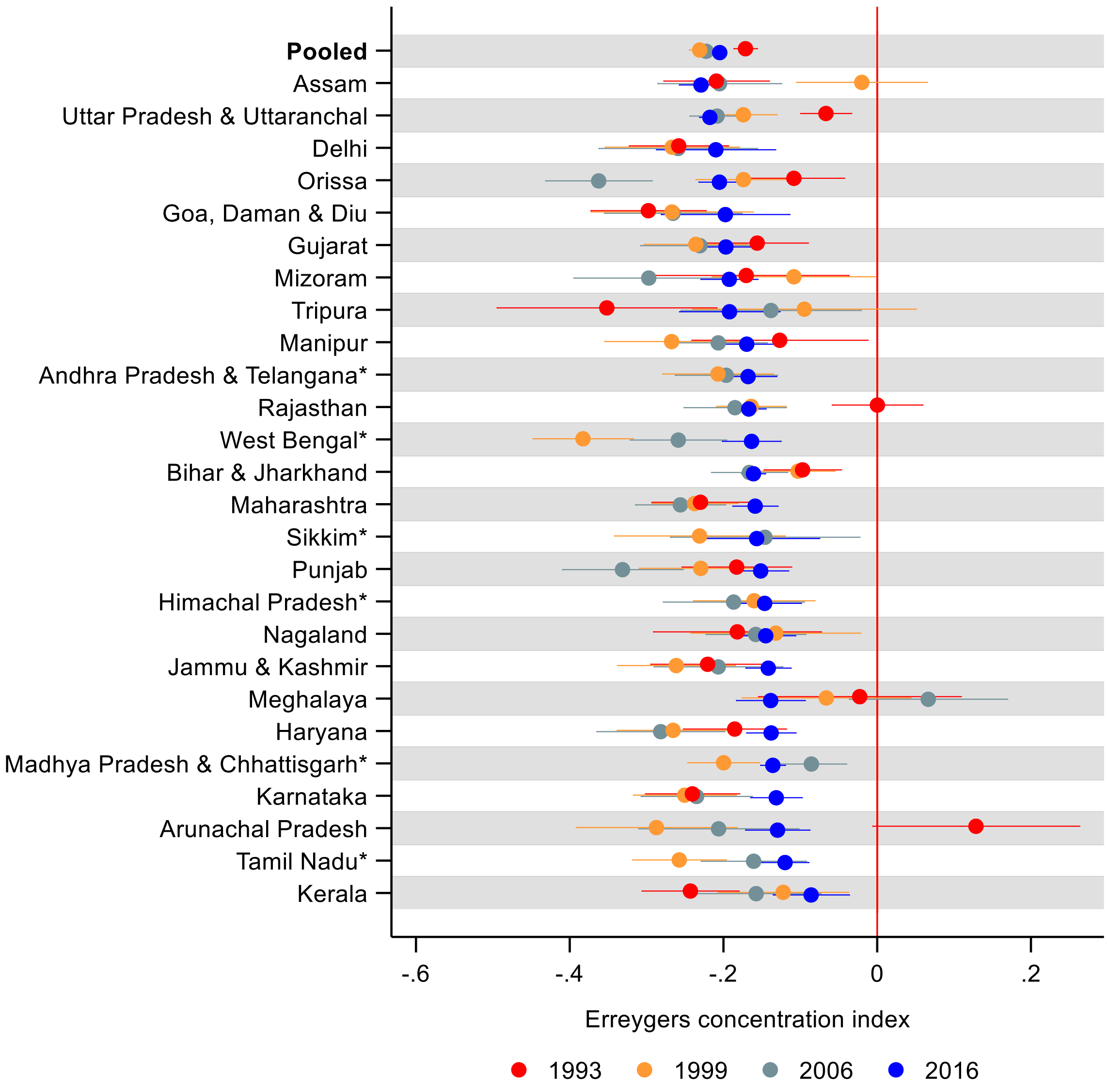

Note: The Erreygers concentration index is bounded with 0–1 limits using a ranking according to state and survey-year specific household wealth index factor score. State-level estimates are ordered from greatest to lowest inequality in 2016. 95% confidence intervals are shown. Estimates are weighted using sampling weights and confidence intervals were adjusted for clustering at the PSU-level.

Figure S21. Erreygers concentration index for wasting by household wealth

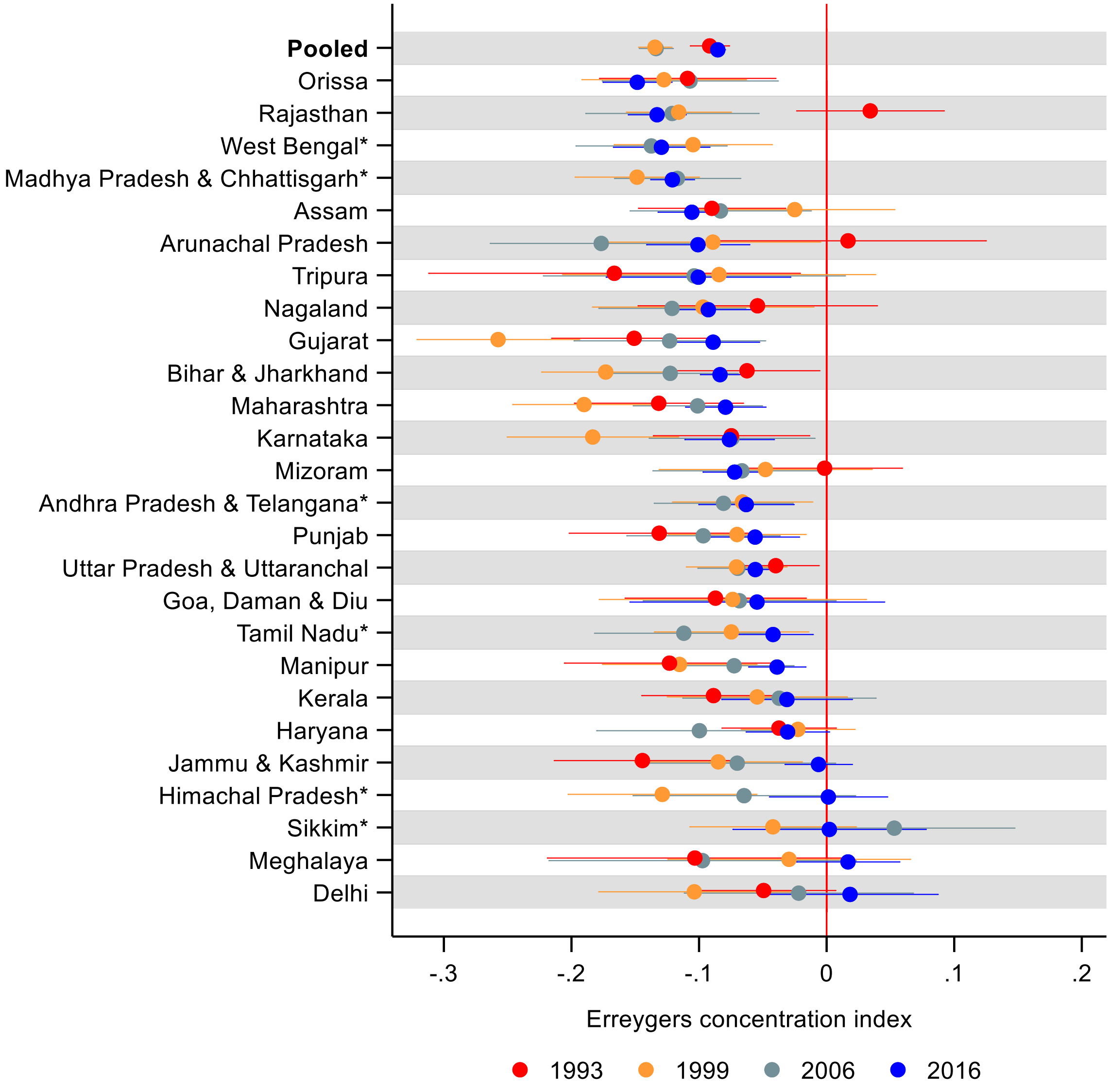

Note: The Erreygers concentration index is bounded with 0–1 limits using a ranking according to state and survey-year specific household wealth index factor score. State-level estimates are ordered from greatest to lowest inequality in 2016. 95% confidence intervals are shown. Estimates are weighted using sampling weights and confidence intervals were adjusted for clustering at the PSU-level.

Figure S22. Modified concentration index for underweight by household wealth

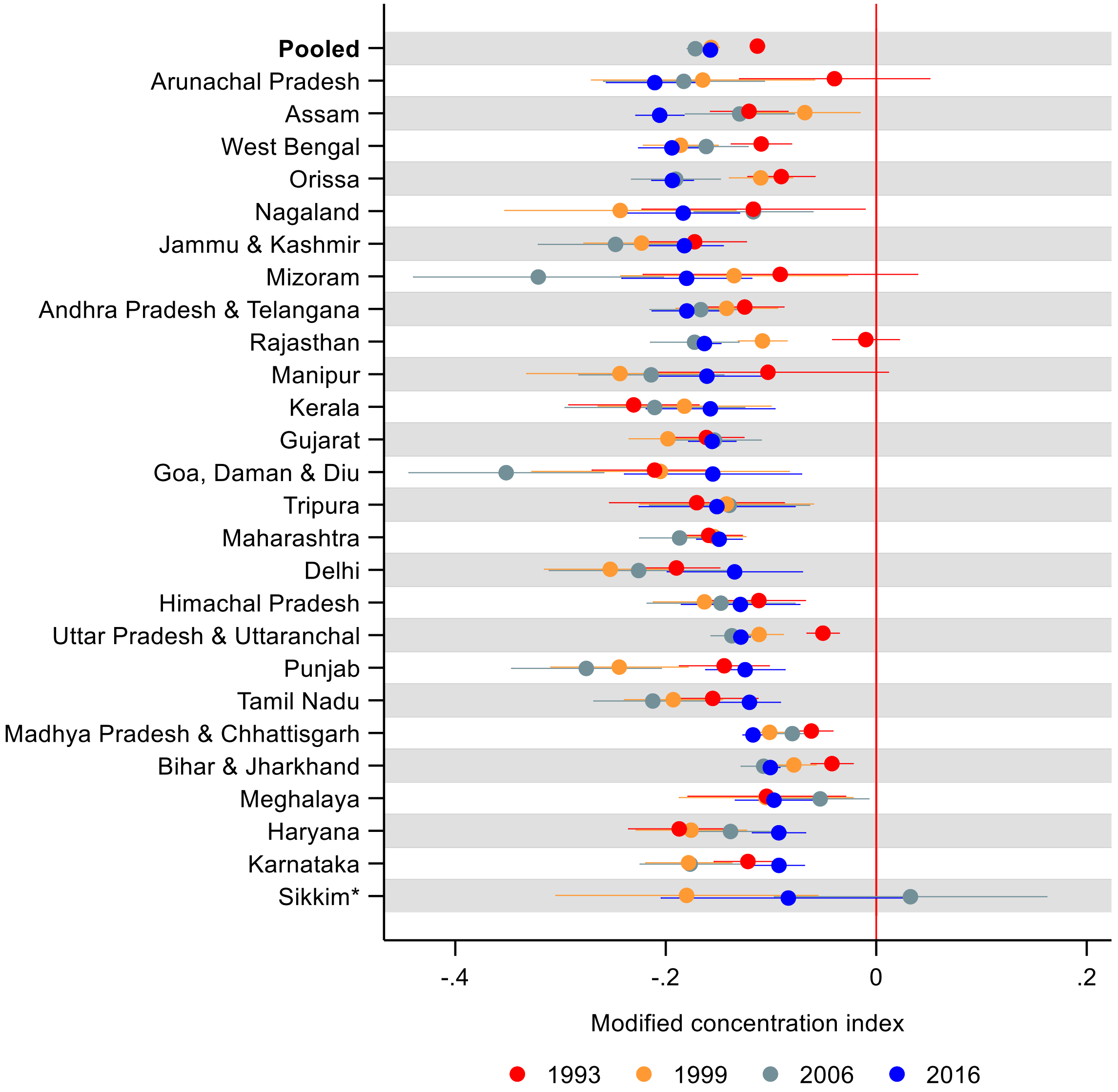

Note: The Modified concentration index is bounded with 0–1 limits using a ranking according to state and survey-year specific household wealth index factor score. State-level estimates are ordered from greatest to lowest inequality in 2016. 95% confidence intervals are shown. Estimates are weighted using sampling weights and confidence intervals were adjusted for clustering at the PSU-level.

Figure S23. Modified concentration index for stunting by household wealth

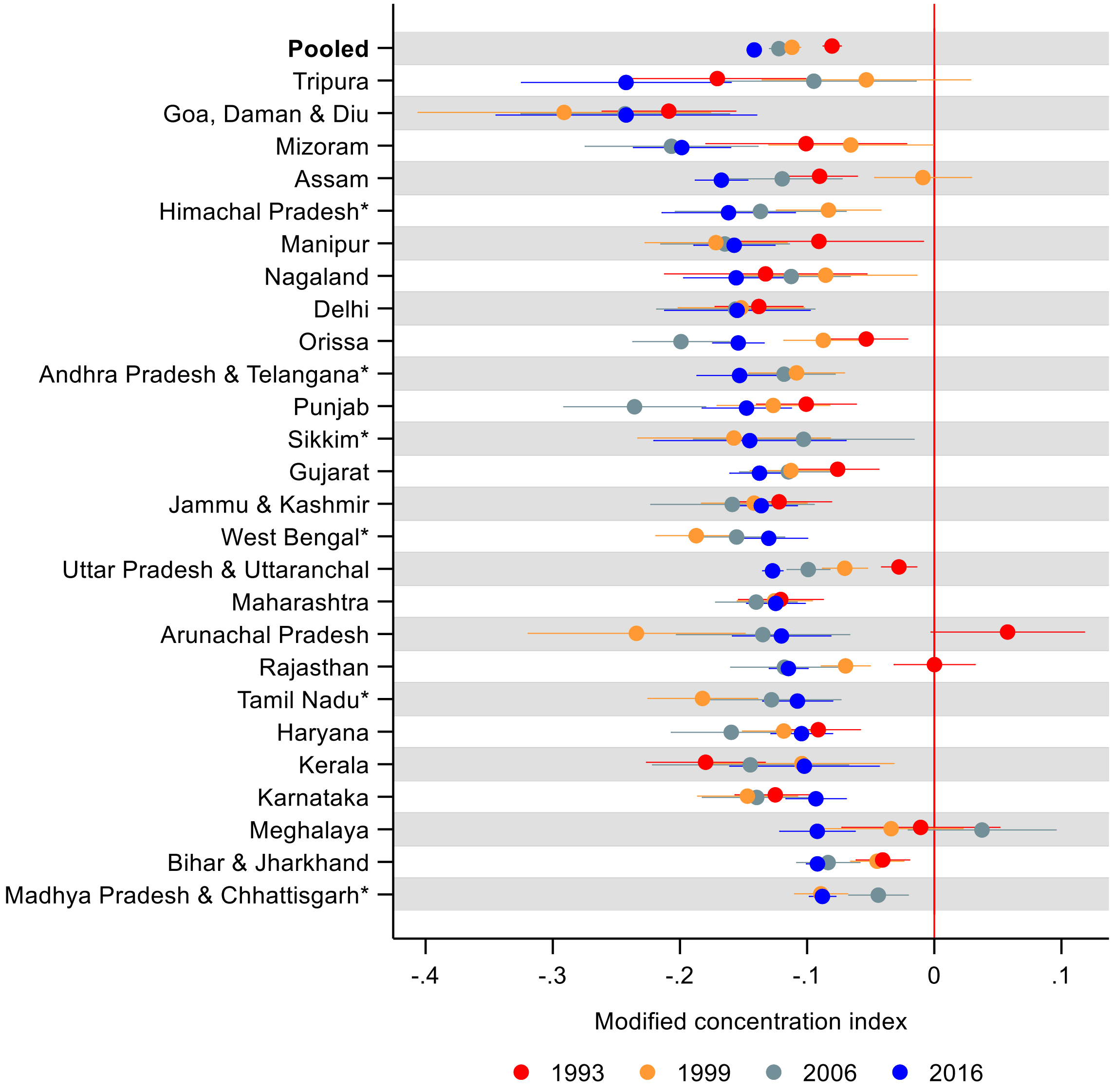

Note: The Modified concentration index is bounded with 0–1 limits using a ranking according to state and survey-year specific household wealth index factor score. State-level estimates are ordered from greatest to lowest inequality in 2016. 95% confidence intervals are shown. Estimates are weighted using sampling weights and confidence intervals were adjusted for clustering at the PSU-level.

Figure S24. Modified concentration index for wasting by household wealth

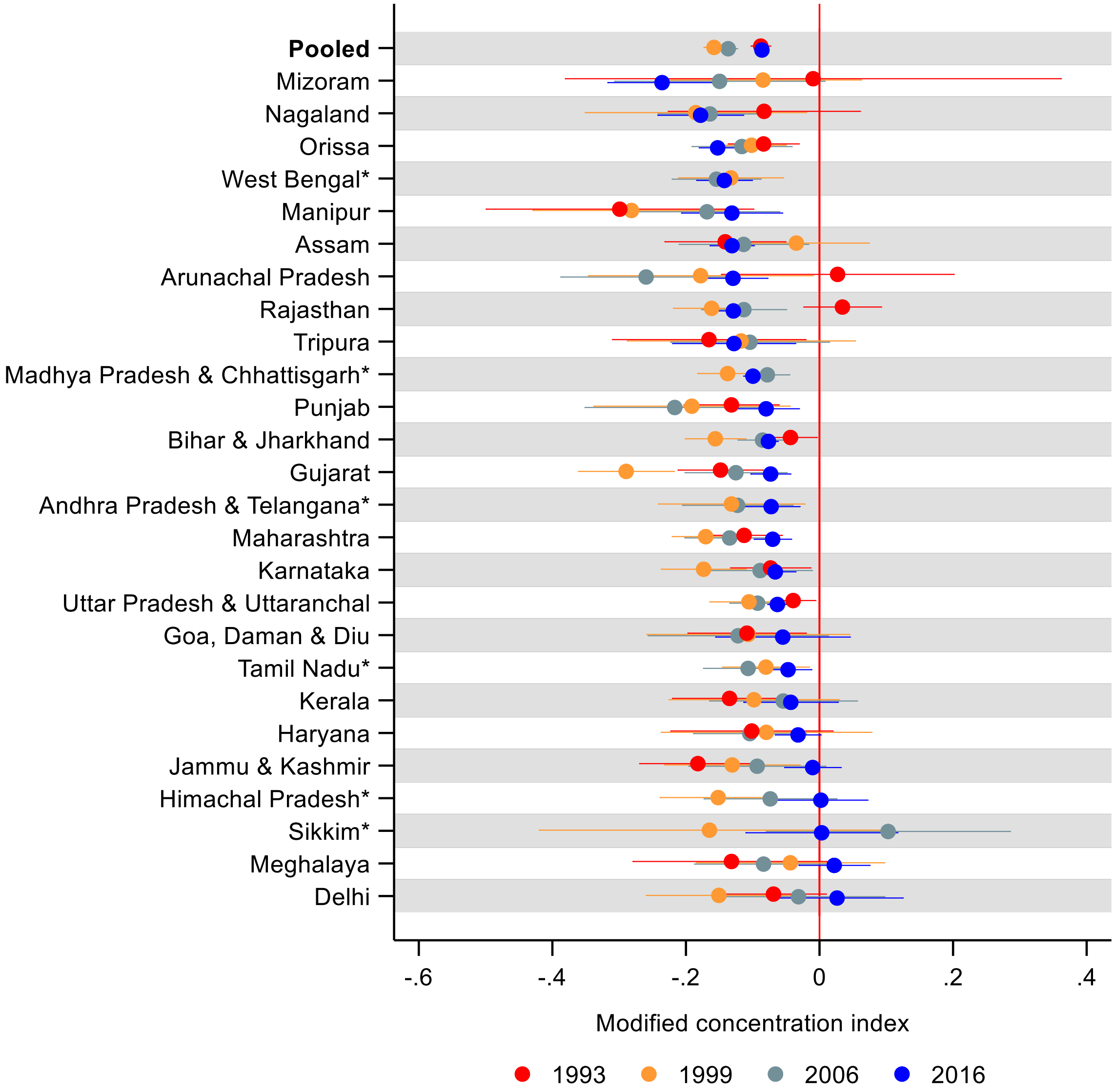

Note: The Modified concentration index is bounded with 0–1 limits using a ranking according to state and survey-year specific household wealth index factor score. State-level estimates are ordered from greatest to lowest inequality in 2016. 95% confidence intervals are shown. Estimates are weighted using sampling weights and confidence intervals were adjusted for clustering at the PSU-level.

Figure S25. Prevalence of underweight and average annual reduction (AAR) in underweight between 1993 and 2016, adjusted for season of interview

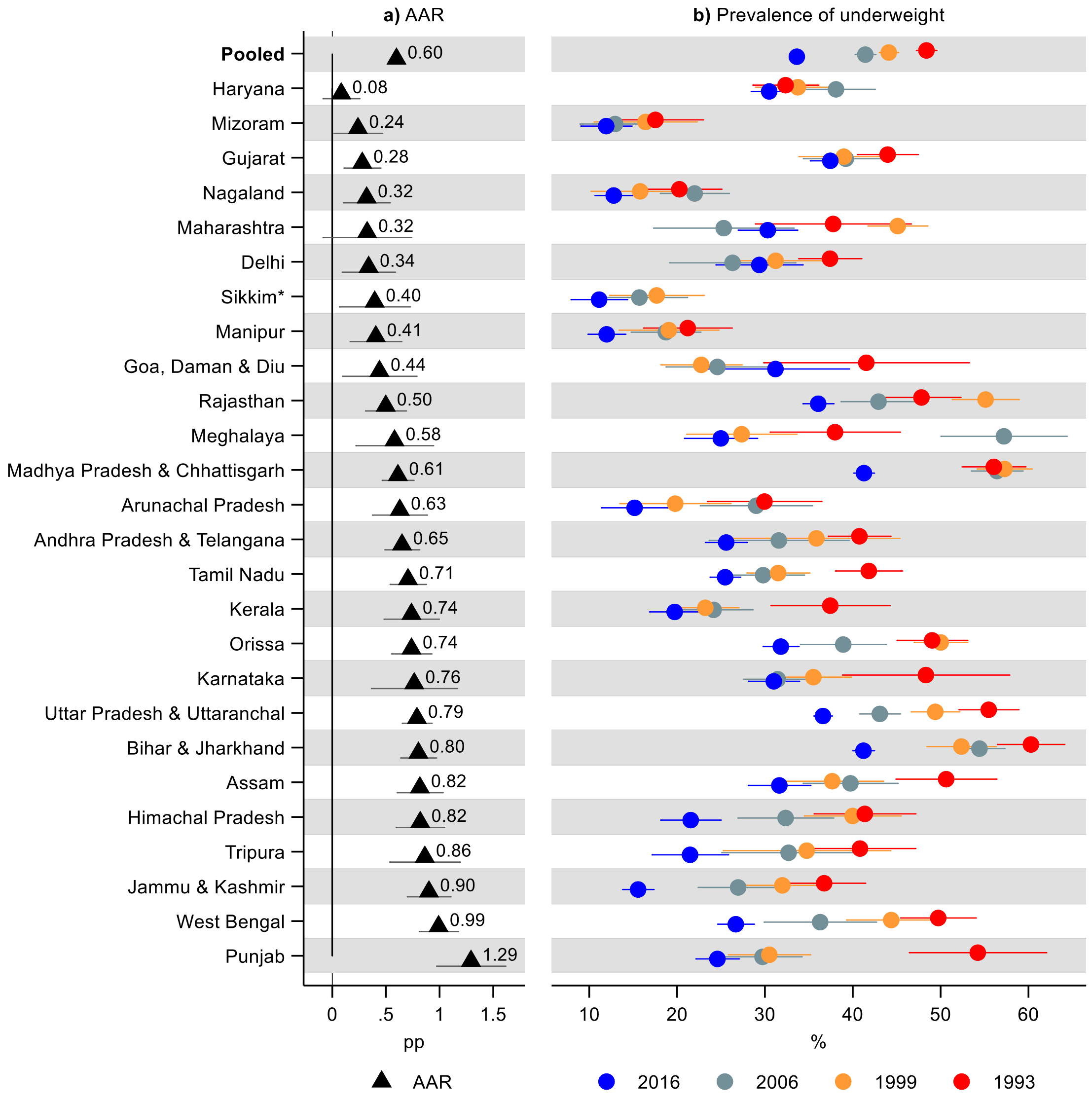

Notes: *Indicates states with no data for 1993: 1999 was used instead. Average annual reduction (AAR) shows average annual percentage point (pp) reduction in prevalence of underweight in each state. 95% confidence intervals are shown. Estimates are weighted using sampling weights and confidence intervals were adjusted for clustering at the PSU-level.

Figure S26. Prevalence of stunting and average annual reduction (AAR) in stunting between 1993 and 2016, adjusted for season of interview

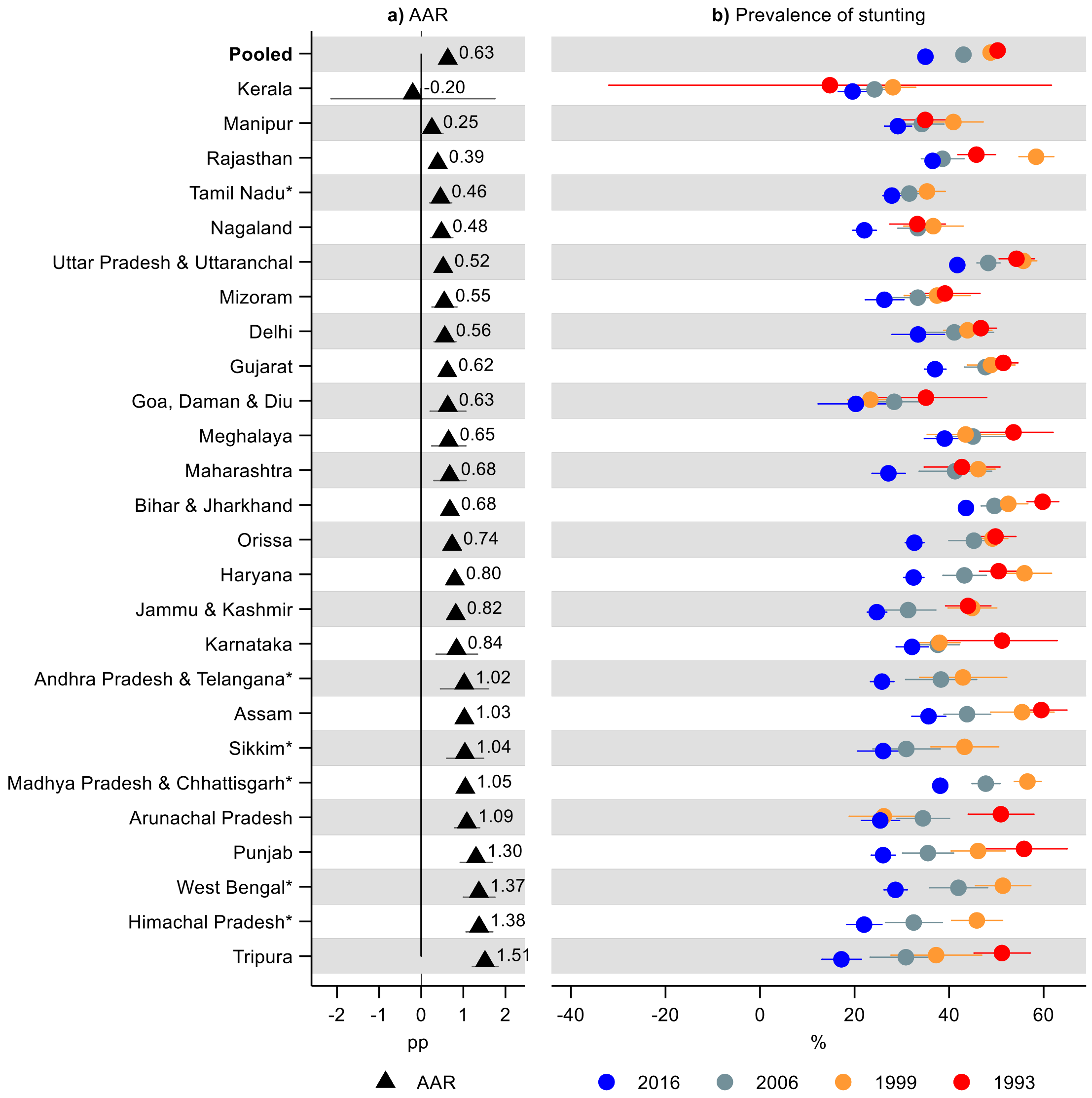

Notes: *Indicates states with no data for 1993: 1999 was used instead. Average annual reduction (AAR) shows average annual percentage point (pp) reduction in prevalence of stunting in each state. 95% confidence intervals are shown. Estimates are weighted using sampling weights and confidence intervals were adjusted for clustering at the PSU-level.

Figure S27. Prevalence of wasting and average annual reduction (AAR) in wasting between 1993 and 2016, adjusted for season of interview

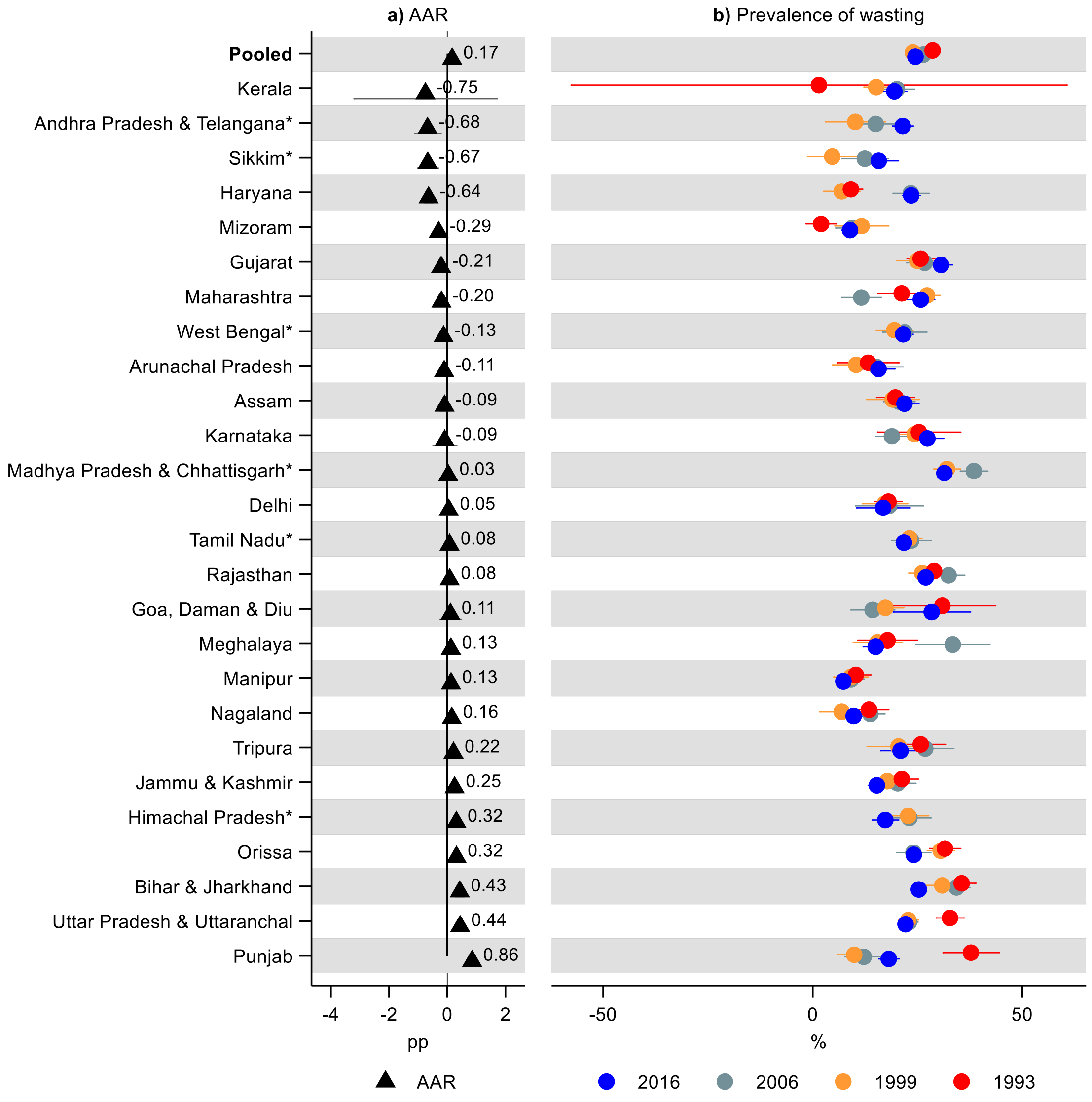

Notes: *Indicates states with no data for 1993: 1999 was used instead. Average annual reduction (AAR) shows average annual percentage point (pp) reduction in prevalence of wasting in each state. 95% confidence intervals are shown. Estimates are weighted using sampling weights and confidence intervals were adjusted for clustering at the PSU-level.

Figure S28. Prevalence of underweight and average annual reduction (AAR) in underweight between 1993 and 2016, males

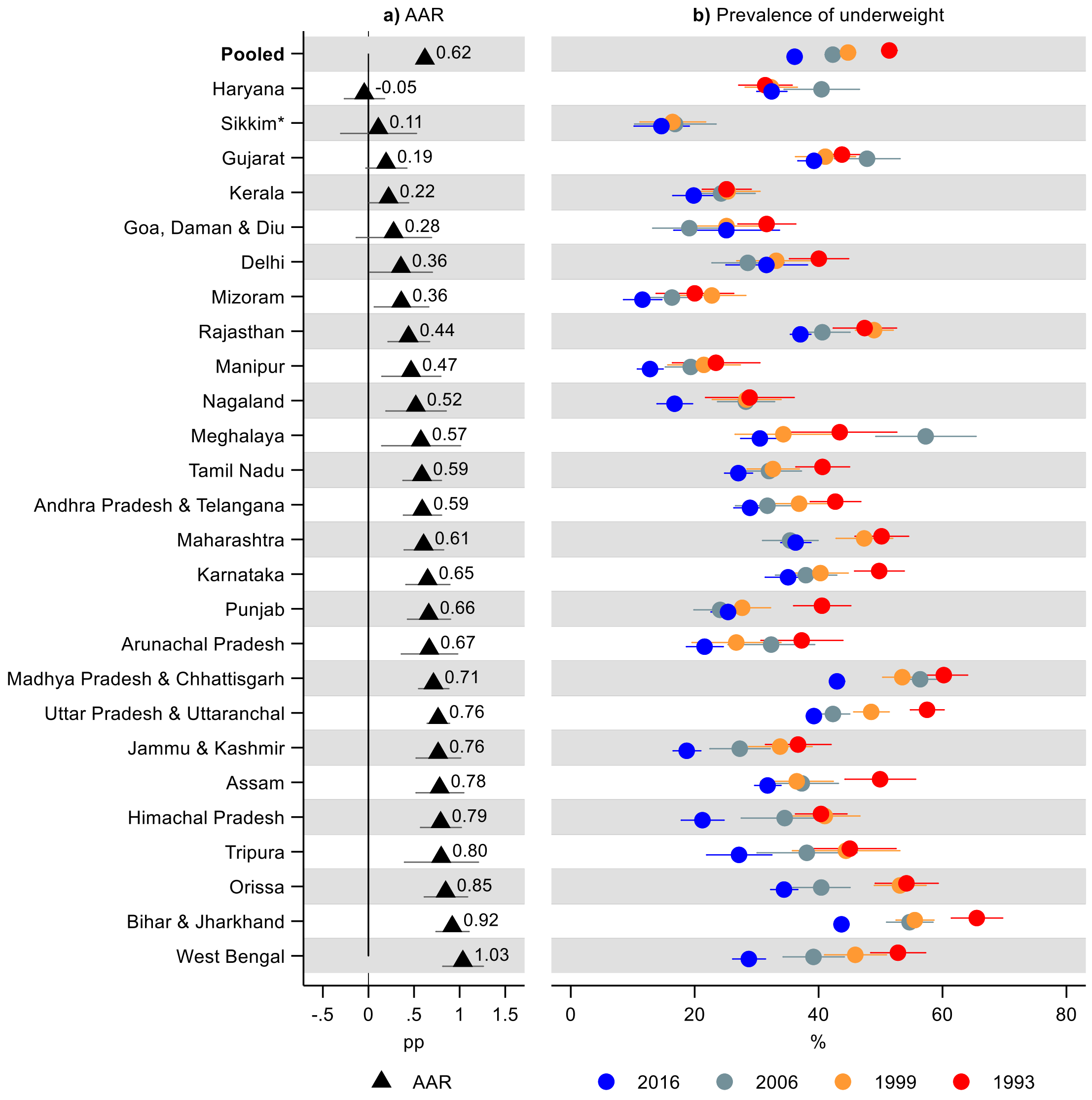

Notes: *Indicates states with no data for 1993: 1999 was used instead. Average annual reduction (AAR) shows average annual percentage point (pp) reduction in prevalence of underweight in each state. 95% confidence intervals are shown. Estimates are weighted using sampling weights and confidence intervals were adjusted for clustering at the PSU-level.

Figure S29. Prevalence of stunting and average annual reduction (AAR) in stunting between 1993 and 2016, males

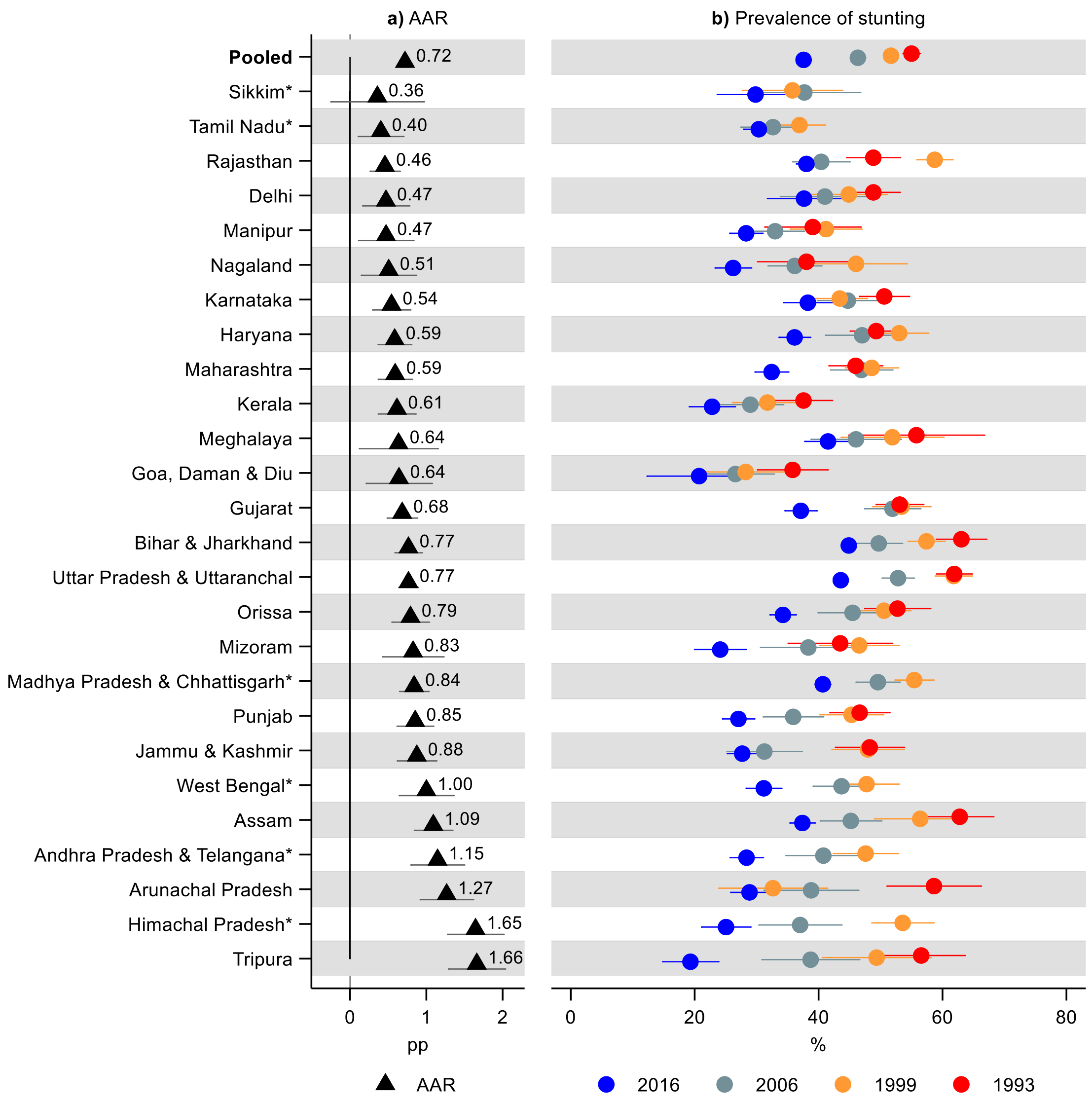

Notes: *Indicates states with no data for 1993: 1999 was used instead. Average annual reduction (AAR) shows average annual percentage point (pp) reduction in prevalence of stunting in each state. 95% confidence intervals are shown. Estimates are weighted using sampling weights and confidence intervals were adjusted for clustering at the PSU-level.

Figure S30. Prevalence of wasting and average annual reduction (AAR) in wasting between 1993 and 2016, males

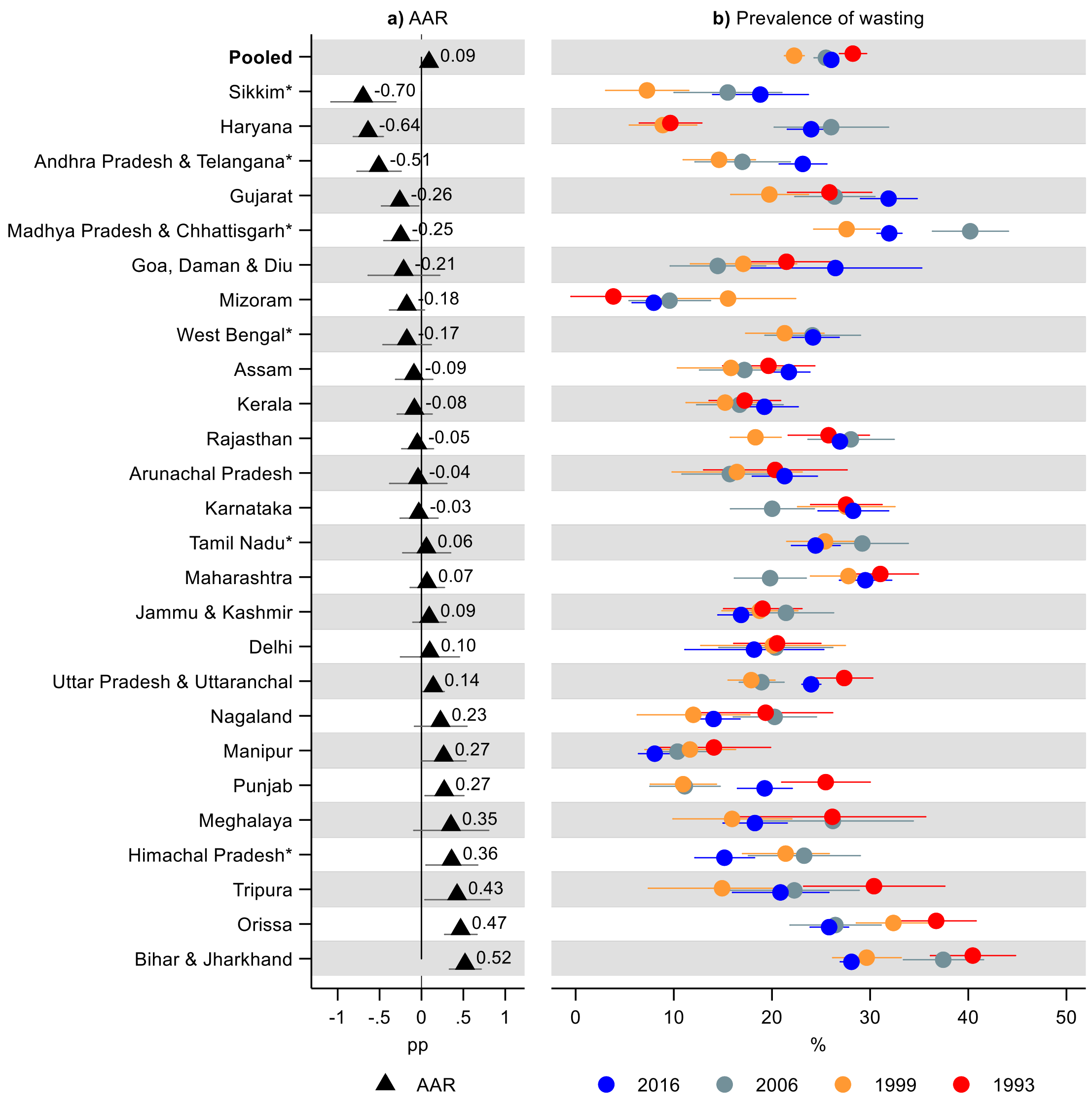

Notes: *Indicates states with no data for 1993: 1999 was used instead. Average annual reduction (AAR) shows average annual percentage point (pp) reduction in prevalence of wasting in each state. 95% confidence intervals are shown. Estimates are weighted using sampling weights and confidence intervals were adjusted for clustering at the PSU-level.

Figure S31. Prevalence of underweight and average annual reduction (AAR) in underweight between 1993 and 2016, females

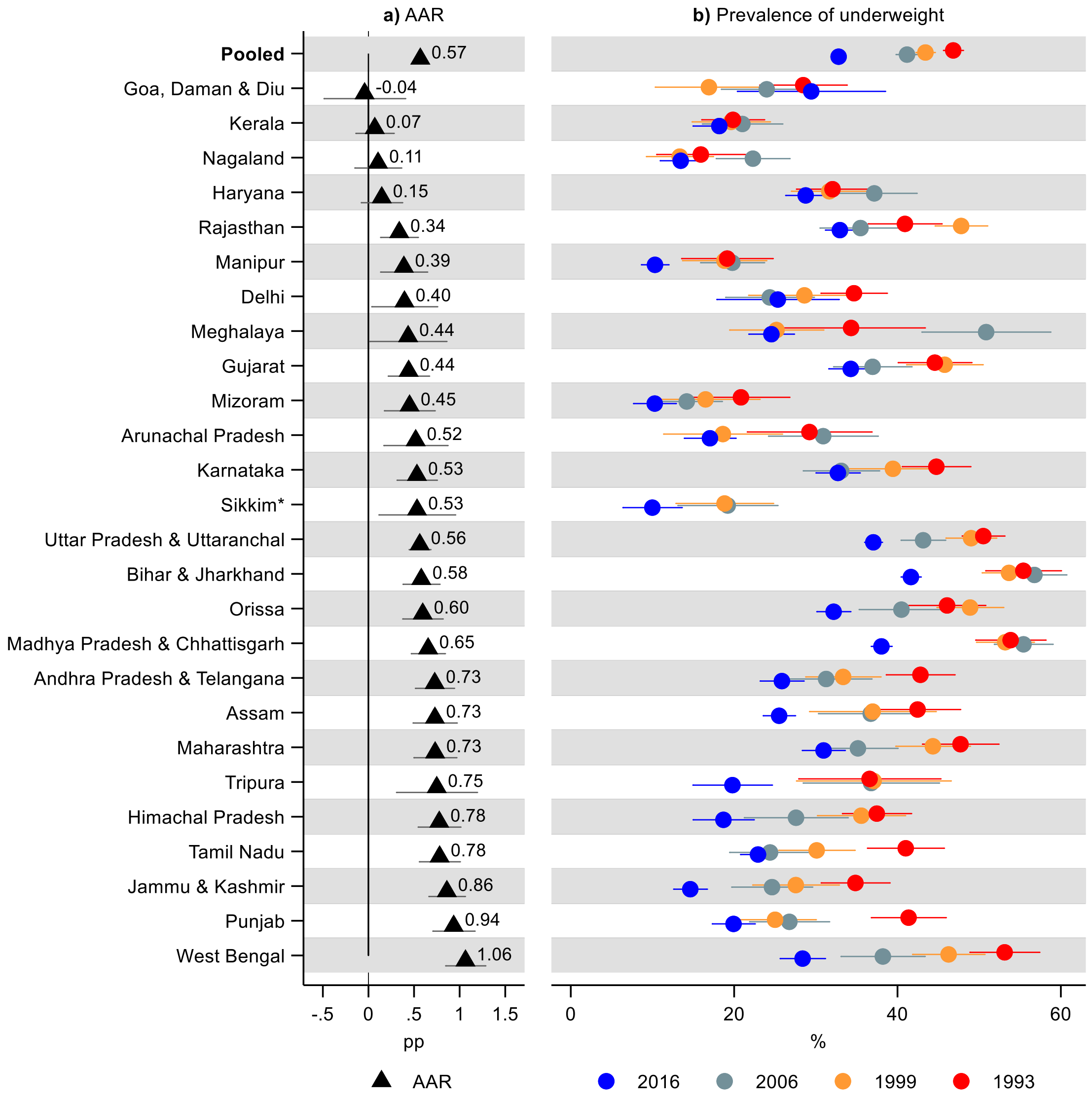

Notes: *Indicates states with no data for 1993: 1999 was used instead. Average annual reduction (AAR) shows average annual percentage point (pp) reduction in prevalence of underweight in each state. 95% confidence intervals are shown. Estimates are weighted using sampling weights and confidence intervals were adjusted for clustering at the PSU-level.

Figure S32. Prevalence of stunting and average annual reduction (AAR) in stunting between 1993 and 2016, females

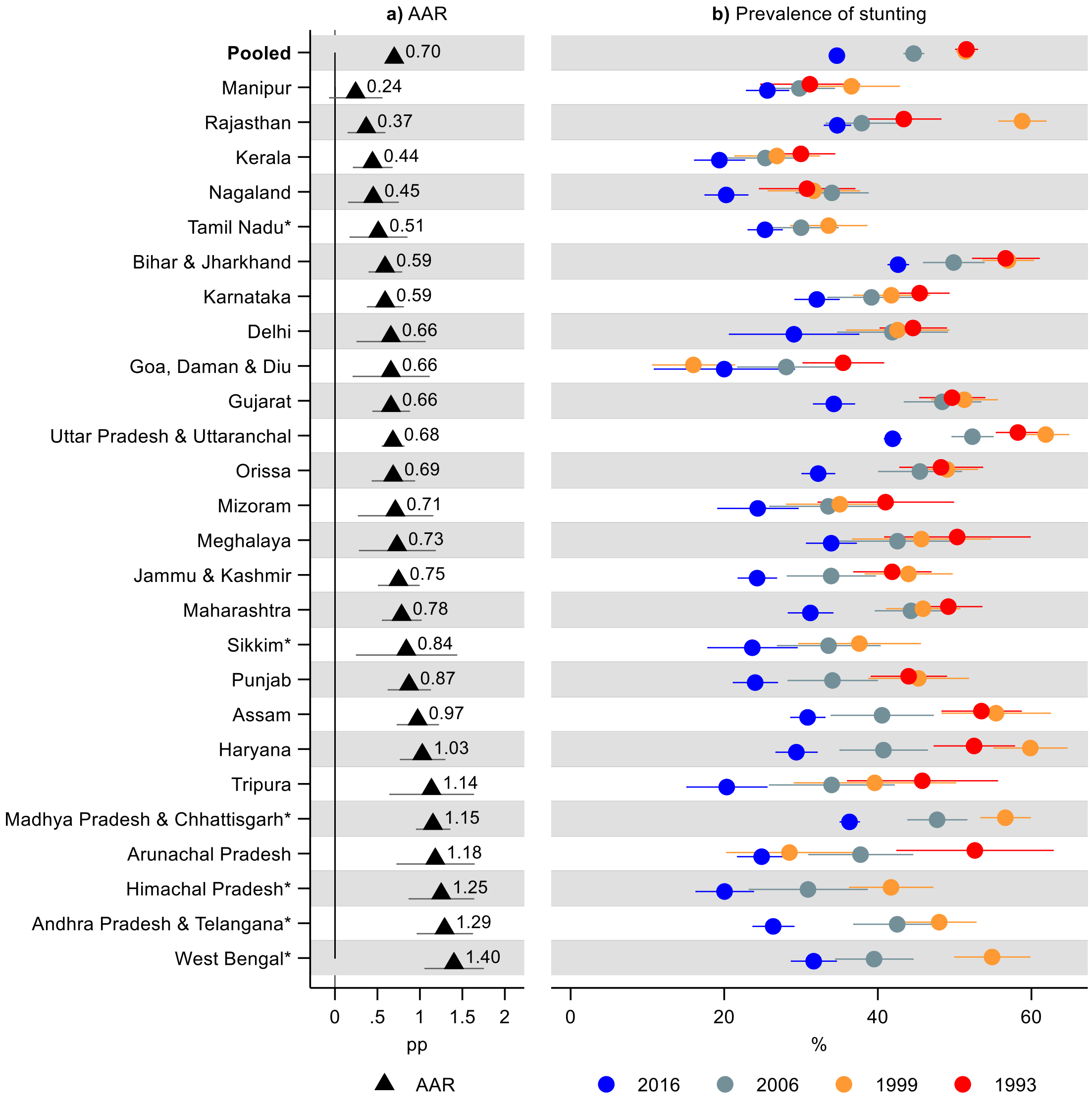

Notes: *Indicates states with no data for 1993: 1999 was used instead. Average annual reduction (AAR) shows average annual percentage point (pp) reduction in prevalence of stunting in each state. 95% confidence intervals are shown. Estimates are weighted using sampling weights and confidence intervals were adjusted for clustering at the PSU-level.

Figure S33. Prevalence of wasting and average annual reduction (AAR) in wasting between 1993 and 2016, females

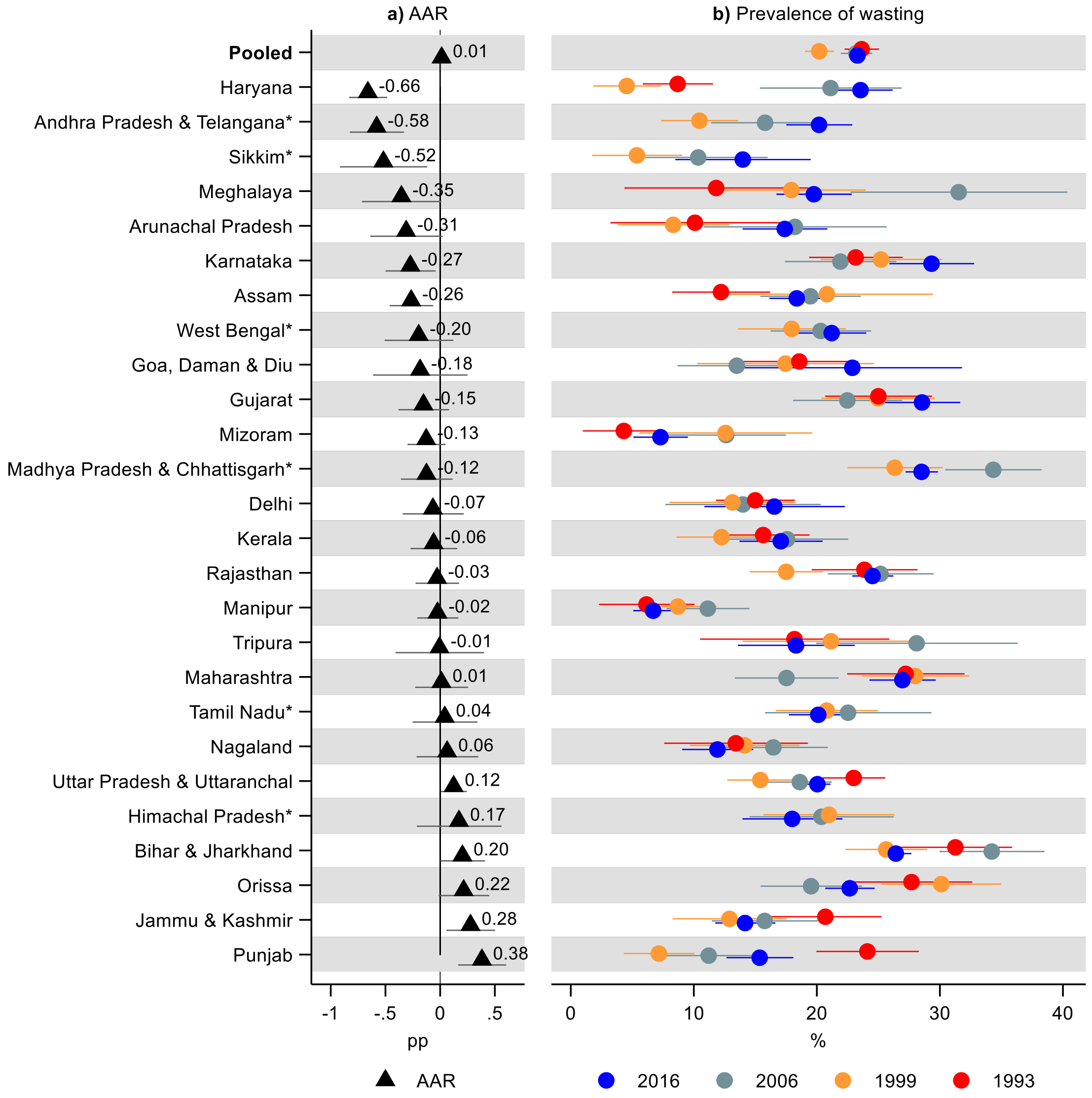

Notes: *Indicates states with no data for 1993: 1999 was used instead. Average annual reduction (AAR) shows average annual percentage point (pp) reduction in prevalence of wasting in each state. 95% confidence intervals are shown. Estimates are weighted using sampling weights and confidence intervals were adjusted for clustering at the PSU-level.

Figure S34. Changes in the poor-rich gap in prevalence of underweight, males

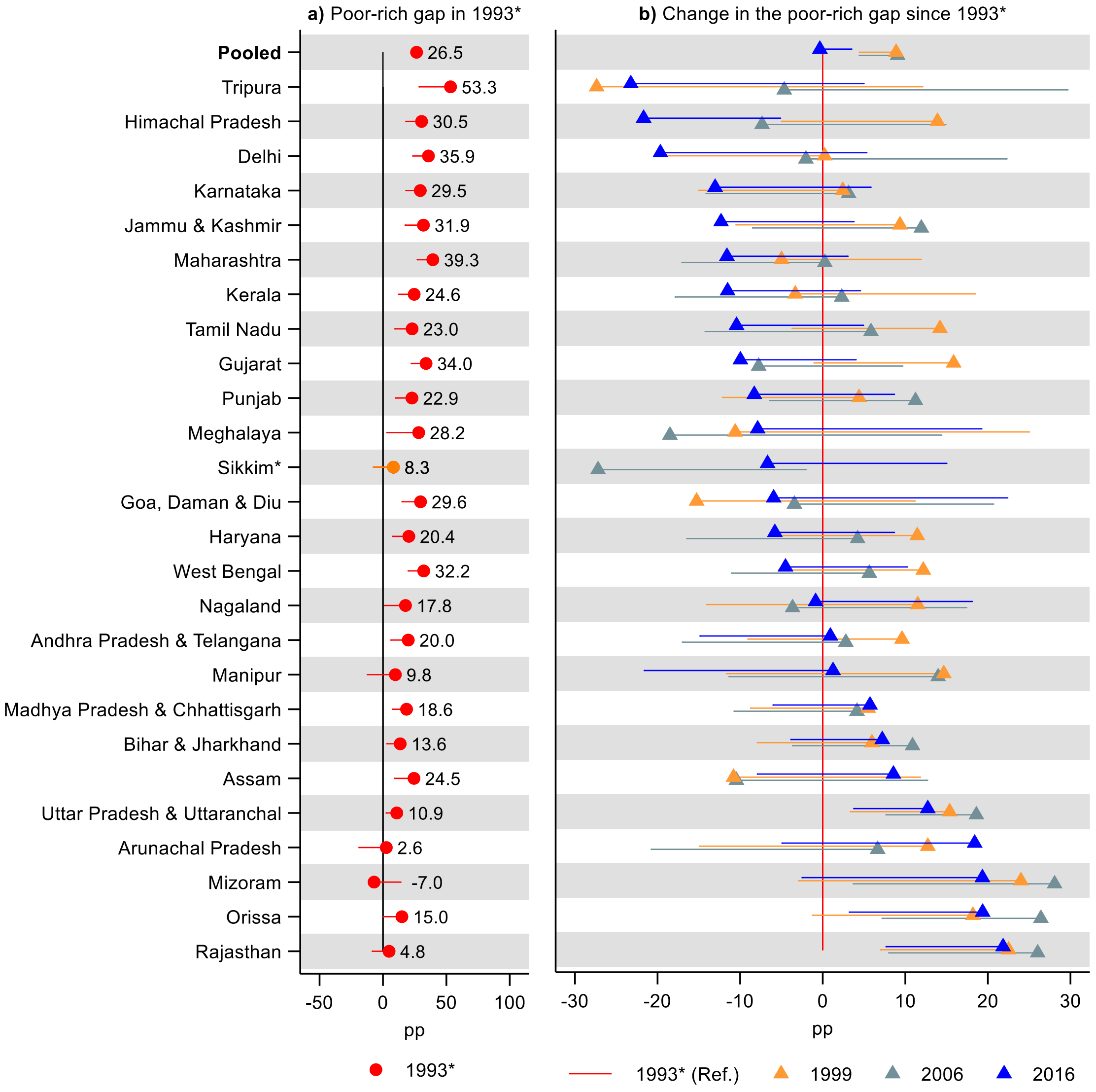

Notes: Percentage point (pp) differences are shown. In Panel a) a positive estimate indicates disadvantage for the poorest children, compared to the richest children, in 1993. A negative estimate in Panel b) indicates that the poor-rich gap (which usually shows a poor disadvantage in Panel a) has shrunk since 1993. The estimates were obtained from an interaction model (OLS) for each state: Panel a) shows the terms for the poorest wealth quintile and Panel b) shows the interaction terms (ie, between poorest quintile and year). The terms for year as well as all terms involving quintiles other than the poorest are excluded from the figure. Vertical lines (at 0) indicate no poor-rich difference in Panel a) and no change in rich-poor gap in Panel b). *Indicates states with no data for 1993: 1999 was used instead. 95% confidence bounds are shown. Only one confidence bound is shown to improve readability: an upper bound where the estimated difference was lower than 0 and a lower bound where the estimated difference was greater than zero. Estimates are weighted using sampling weights and confidence intervals were adjusted for clustering at the PSU-level.

Figure S35. Changes in the poor-rich gap in prevalence of stunting, males

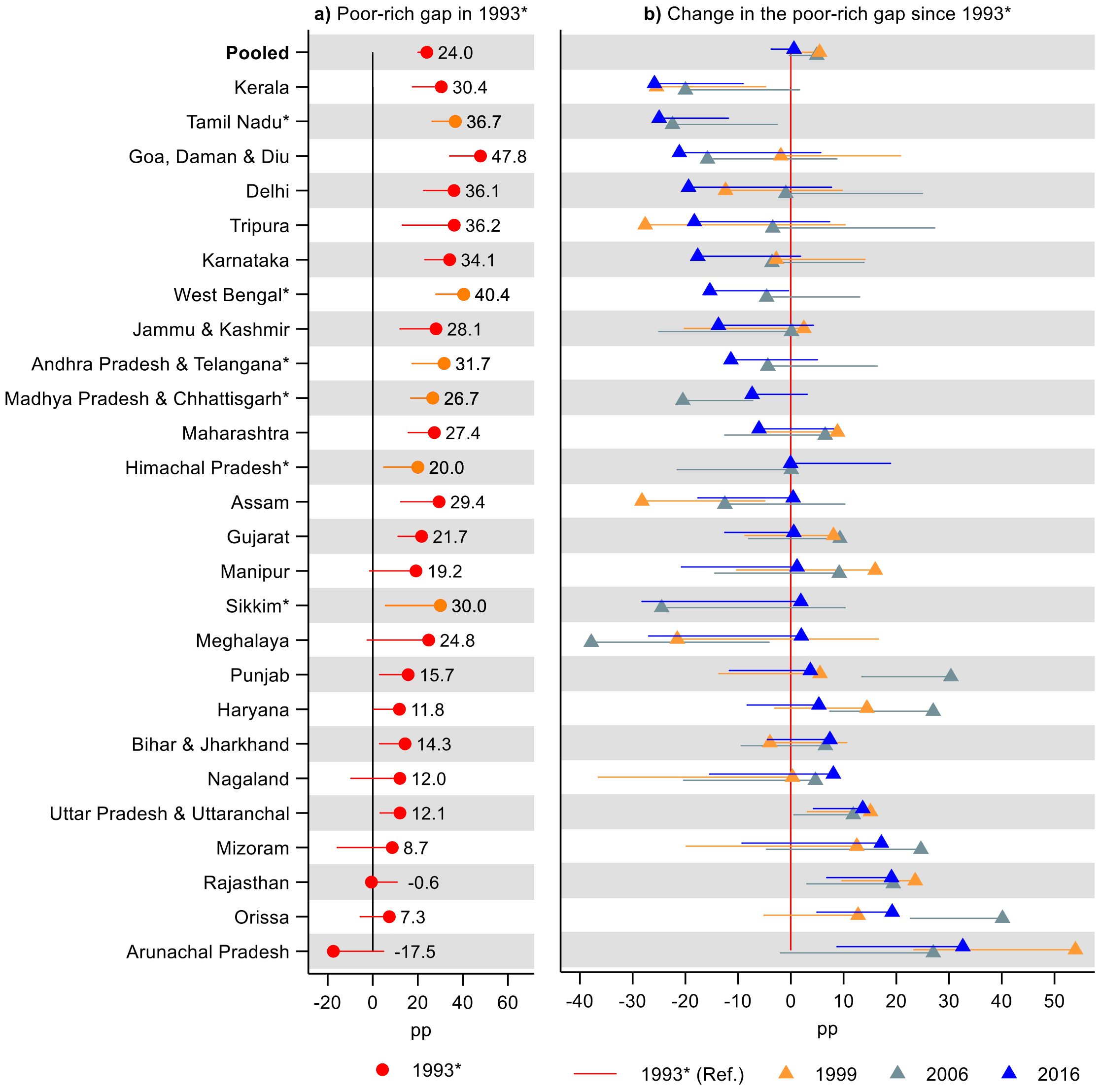

Notes: Percentage point (pp) differences are shown. In Panel a) a positive estimate indicates disadvantage for the poorest children, compared to the richest children, in 1993. A negative estimate in Panel b) indicates that the poor-rich gap (which usually shows a poor disadvantage in Panel a) has shrunk since 1993. The estimates were obtained from an interaction model (OLS) for each state: Panel a) shows the terms for the poorest wealth quintile and Panel b) shows the interaction terms (ie, between poorest quintile and year). The terms for year as well as all terms involving quintiles other than the poorest are excluded from the figure. Vertical lines (at 0) indicate no poor-rich difference in Panel a) and no change in rich-poor gap in Panel b). *Indicates states with no data for 1993: 1999 was used instead. 95% confidence bounds are shown. Only one confidence bound is shown to improve readability: an upper bound where the estimated difference was lower than 0 and a lower bound where the estimated difference was greater than zero. Estimates are weighted using sampling weights and confidence intervals were adjusted for clustering at the PSU-level.

Figure S36. Changes in the poor-rich gap in prevalence of wasting, males

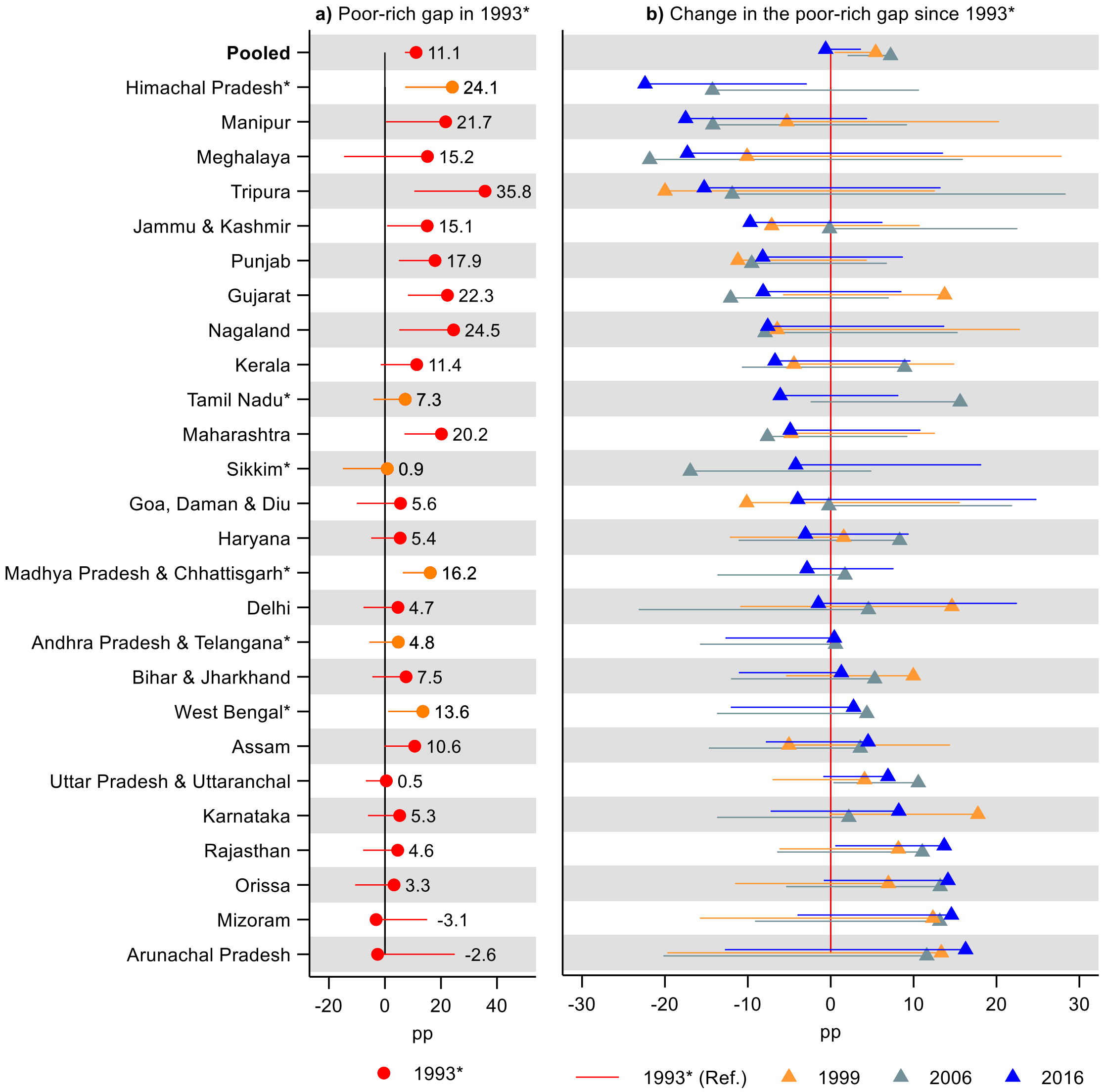

Notes: Percentage point (pp) differences are shown. In Panel a) a positive estimate indicates disadvantage for the poorest children, compared to the richest children, in 1993. A negative estimate in Panel b) indicates that the poor-rich gap (which usually shows a poor disadvantage in Panel a) has shrunk since 1993. The estimates were obtained from an interaction model (OLS) for each state: Panel a) shows the terms for the poorest wealth quintile and Panel b) shows the interaction terms (ie, between poorest quintile and year). The terms for year as well as all terms involving quintiles other than the poorest are excluded from the figure. Vertical lines (at 0) indicate no poor-rich difference in Panel a) and no change in rich-poor gap in Panel b). *Indicates states with no data for 1993: 1999 was used instead. 95% confidence bounds are shown. Only one confidence bound is shown to improve readability: an upper bound where the estimated difference was lower than 0 and a lower bound where the estimated difference was greater than zero. Estimates are weighted using sampling weights and confidence intervals were adjusted for clustering at the PSU-level.

Figure S37. Changes in the poor-rich gap in prevalence of underweight, females

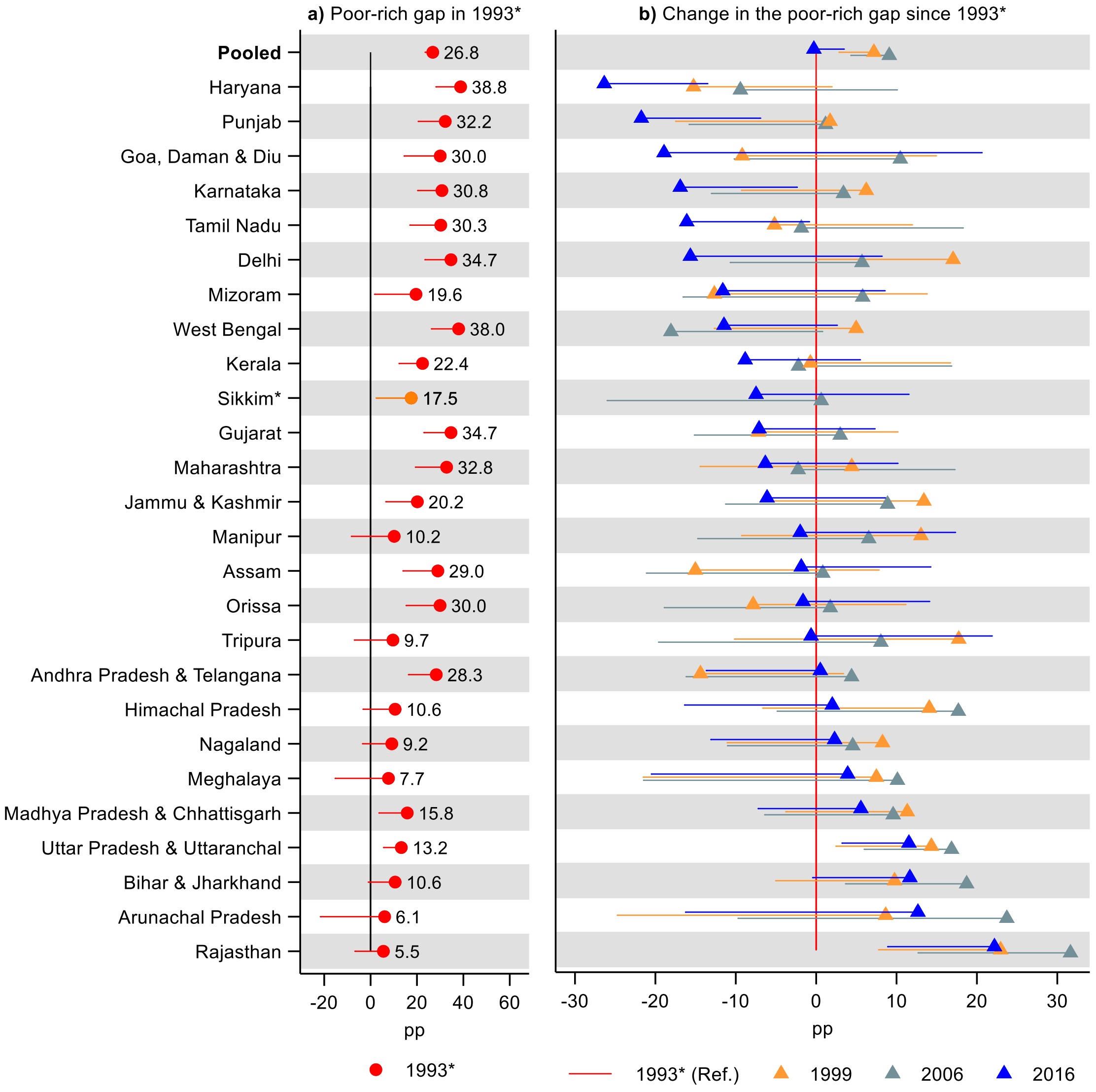

Notes: Percentage point (pp) differences are shown. In Panel a) a positive estimate indicates disadvantage for the poorest children, compared to the richest children, in 1993. A negative estimate in Panel b) indicates that the poor-rich gap (which usually shows a poor disadvantage in Panel a) has shrunk since 1993. The estimates were obtained from an interaction model (OLS) for each state: Panel a) shows the terms for the poorest wealth quintile and Panel b) shows the interaction terms (ie, between poorest quintile and year). The terms for year as well as all terms involving quintiles other than the poorest are excluded from the figure. Vertical lines (at 0) indicate no poor-rich difference in Panel a) and no change in rich-poor gap in Panel b). *Indicates states with no data for 1993: 1999 was used instead. 95% confidence bounds are shown. Only one confidence bound is shown to improve readability: an upper bound where the estimated difference was lower than 0 and a lower bound where the estimated difference was greater than zero. Estimates are weighted using sampling weights and confidence intervals were adjusted for clustering at the PSU-level.

Figure S38. Changes in the poor-rich gap in prevalence of stunting, females

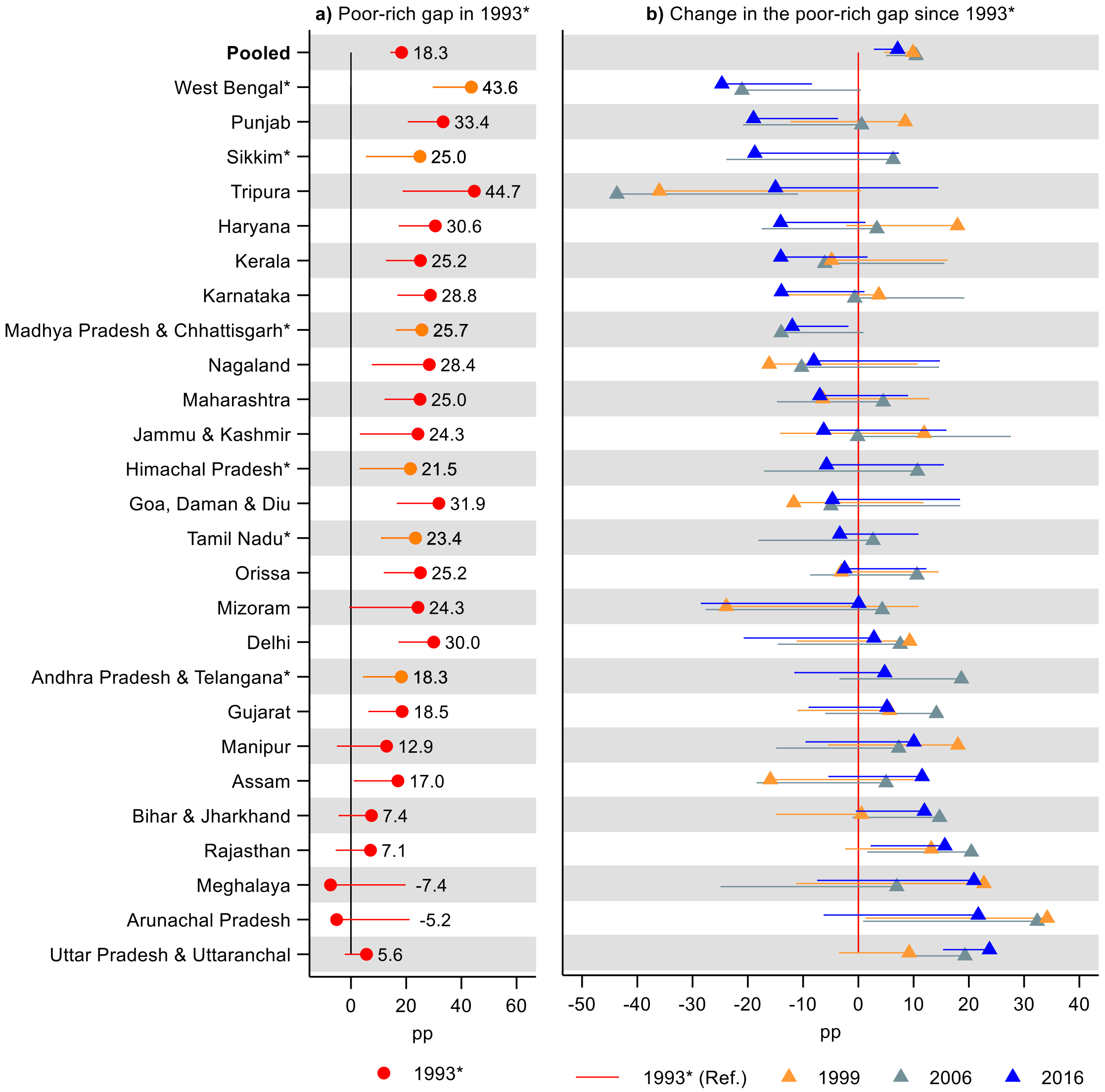

Notes: Percentage point (pp) differences are shown. In Panel a) a positive estimate indicates disadvantage for the poorest children, compared to the richest children, in 1993. A negative estimate in Panel b) indicates that the poor-rich gap (which usually shows a poor disadvantage in Panel a) has shrunk since 1993. The estimates were obtained from an interaction model (OLS) for each state: Panel a) shows the terms for the poorest wealth quintile and Panel b) shows the interaction terms (ie, between poorest quintile and year). The terms for year as well as all terms involving quintiles other than the poorest are excluded from the figure. Vertical lines (at 0) indicate no poor-rich difference in Panel a) and no change in rich-poor gap in Panel b). *Indicates states with no data for 1993: 1999 was used instead. 95% confidence bounds are shown. Only one confidence bound is shown to improve readability: an upper bound where the estimated difference was lower than 0 and a lower bound where the estimated difference was greater than zero. Estimates are weighted using sampling weights and confidence intervals were adjusted for clustering at the PSU-level.

Figure S39. Changes in the poor-rich gap in prevalence of wasting, females

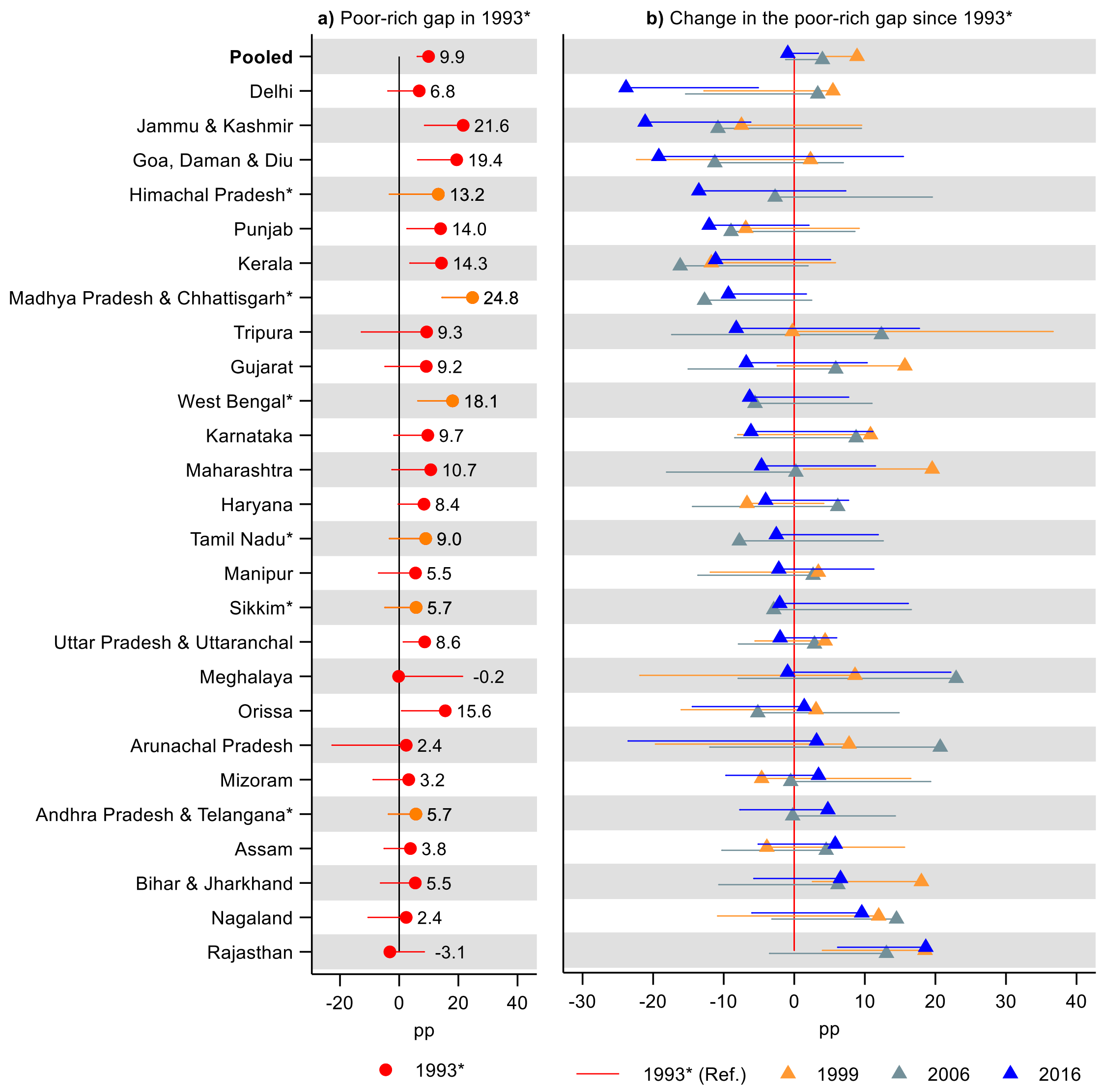

Notes: Percentage point (pp) differences are shown. In Panel a) a positive estimate indicates disadvantage for the poorest children, compared to the richest children, in 1993. A negative estimate in Panel b) indicates that the poor-rich gap (which usually shows a poor disadvantage in Panel a) has shrunk since 1993. The estimates were obtained from an interaction model (OLS) for each state: Panel a) shows the terms for the poorest wealth quintile and Panel b) shows the interaction terms (ie, between poorest quintile and year). The terms for year as well as all terms involving quintiles other than the poorest are excluded from the figure. Vertical lines (at 0) indicate no poor-rich difference in Panel a) and no change in rich-poor gap in Panel b). *Indicates states with no data for 1993: 1999 was used instead. 95% confidence bounds are shown. Only one confidence bound is shown to improve readability: an upper bound where the estimated difference was lower than 0 and a lower bound where the estimated difference was greater than zero. Estimates are weighted using sampling weights and confidence intervals were adjusted for clustering at the PSU-level.

Figure S40. Prevalence of severe underweight and average annual reduction (AAR) in severe underweight between 1993 and 2016

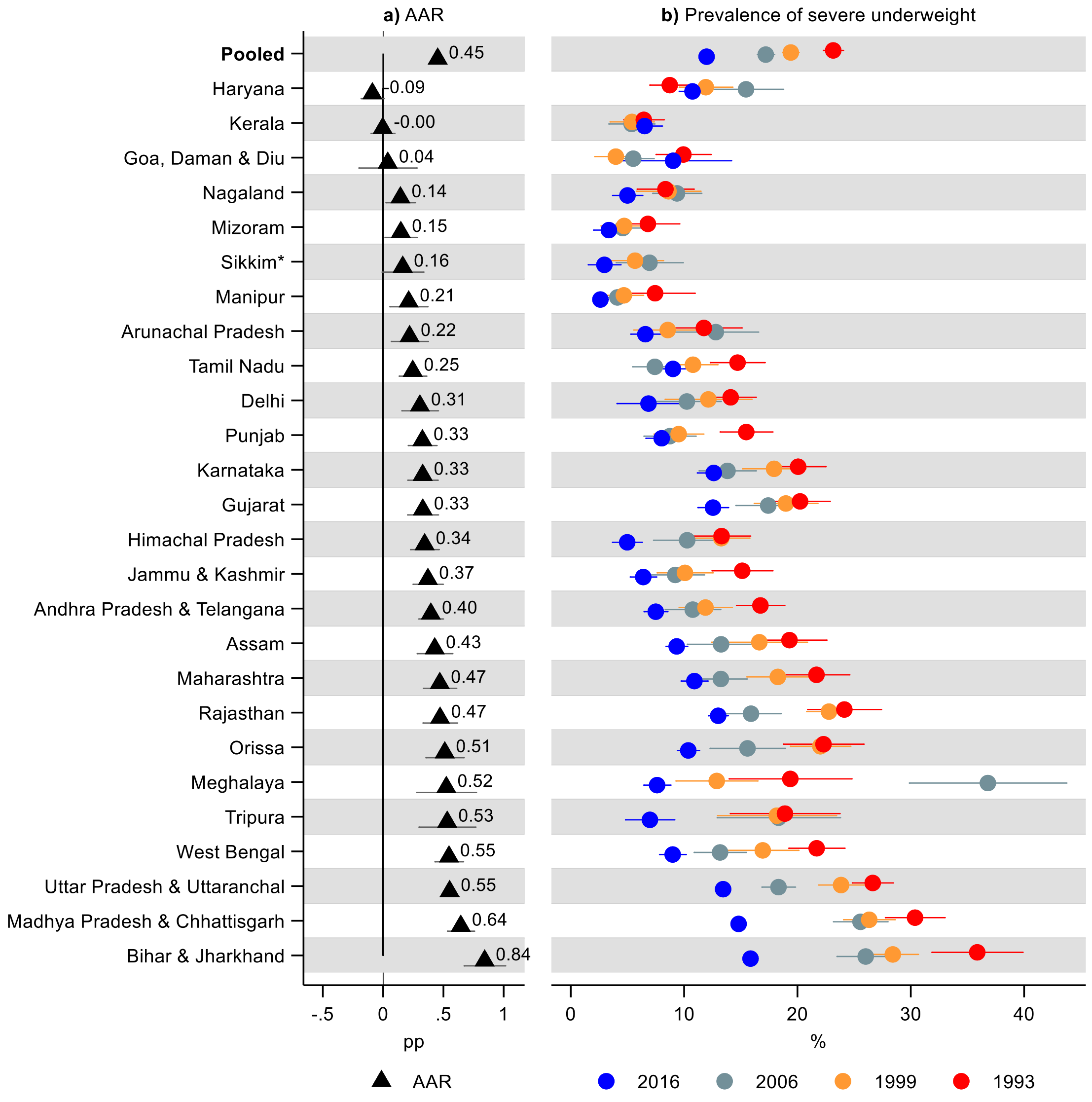

Notes: *Indicates states with no data for 1993: 1999 was used instead. Average annual reduction (AAR) shows average annual percentage point (pp) reduction in prevalence of severe underweight in each state. 95% confidence intervals are shown. Estimates are weighted using sampling weights and confidence intervals were adjusted for clustering at the PSU-level.

Figure S41. Prevalence of severe stunting and average annual reduction (AAR) in severe stunting between 1993 and 2016

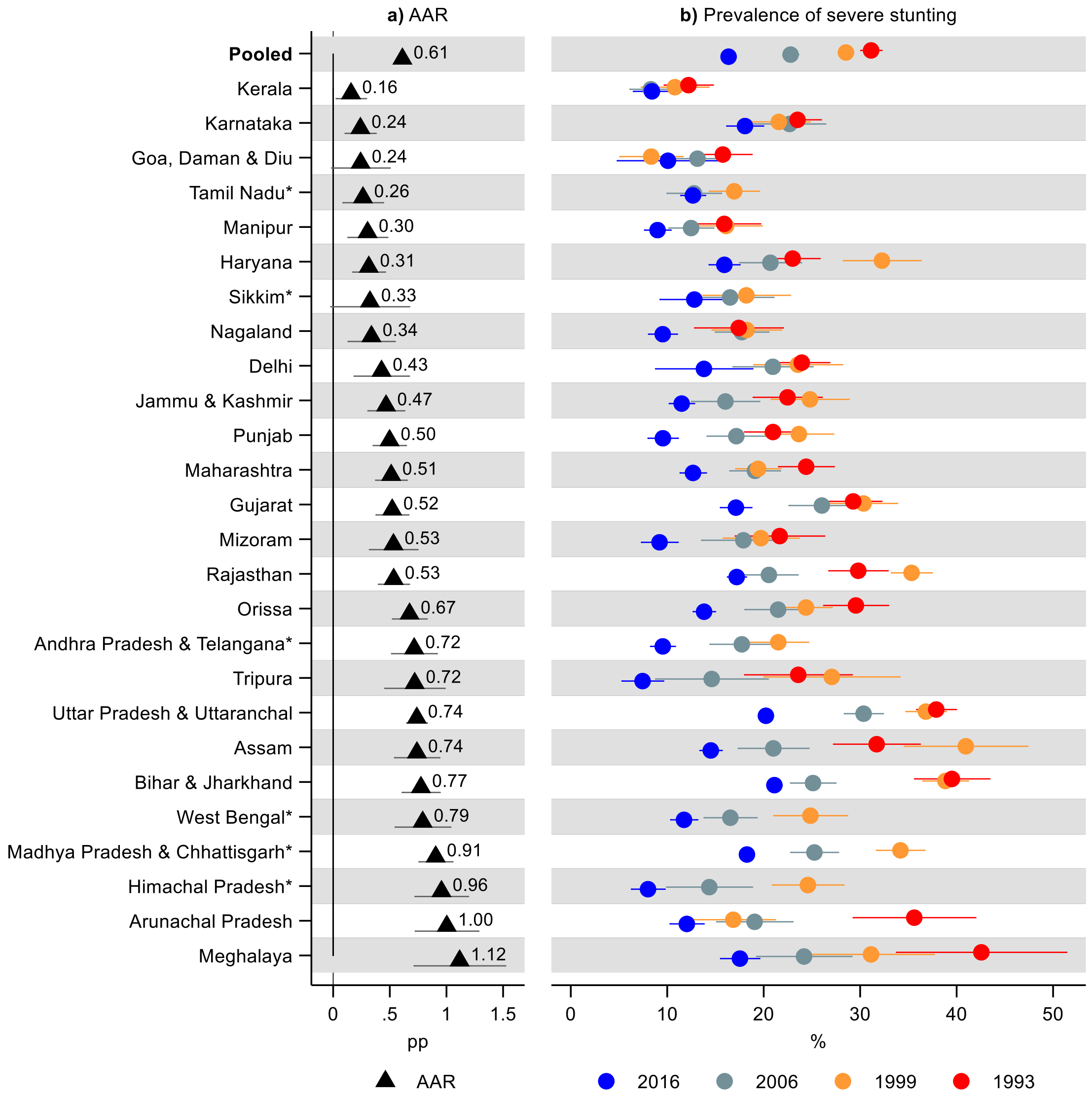

Notes: *Indicates states with no data for 1993: 1999 was used instead. Average annual reduction (AAR) shows average annual percentage point (pp) reduction in prevalence of severe stunting in each state. 95% confidence intervals are shown. Estimates are weighted using sampling weights and confidence intervals were adjusted for clustering at the PSU-level.

Figure S42. Prevalence of severe wasting and average annual reduction (AAR) in severe wasting between 1993 and 2016

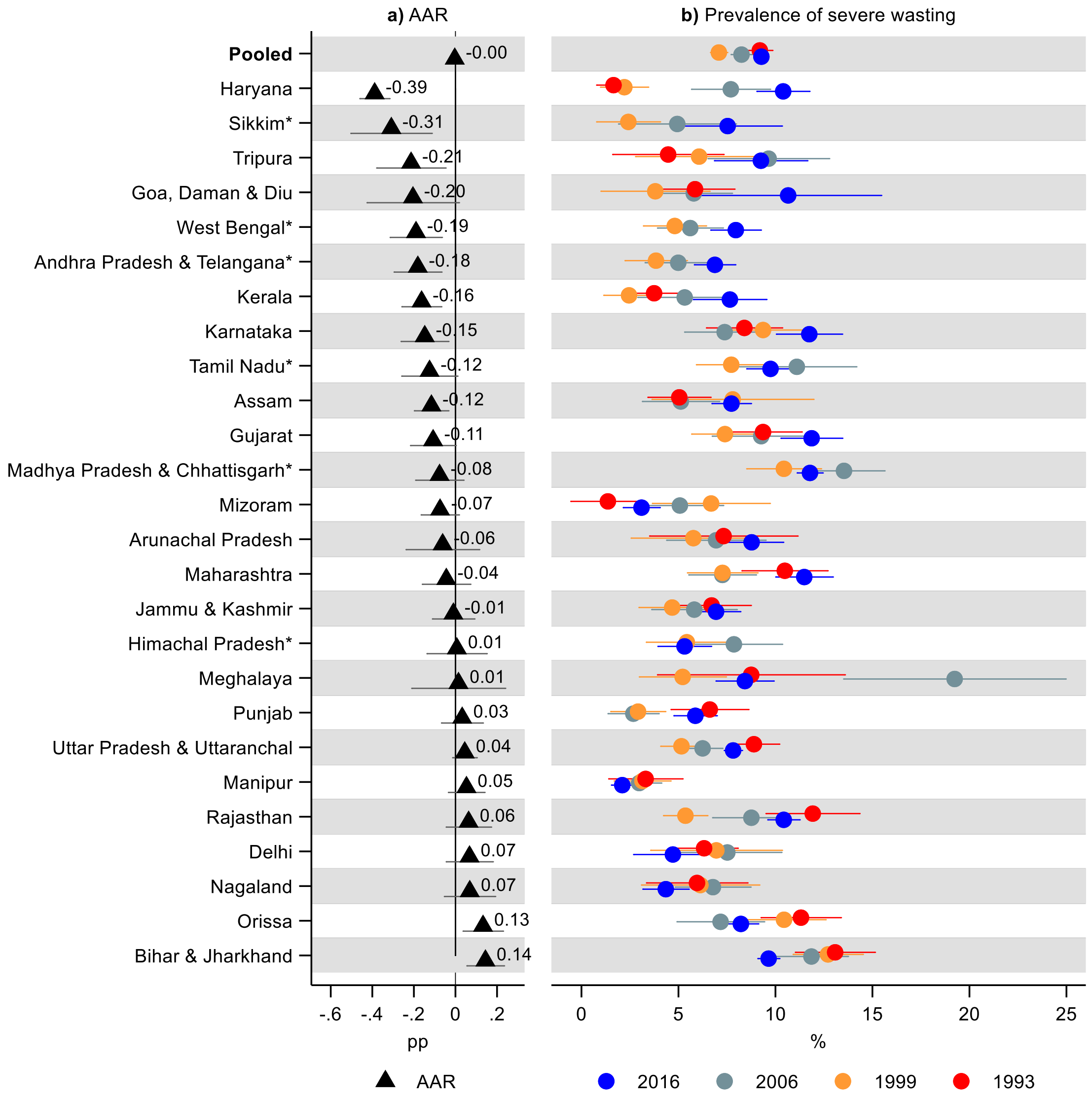

Notes: *Indicates states with no data for 1993: 1999 was used instead. Average annual reduction (AAR) shows average annual percentage point (pp) reduction in prevalence of severe wasting in each state. 95% confidence intervals are shown. Estimates are weighted using sampling weights and confidence intervals were adjusted for clustering at the PSU-level.

Figure S43. Changes in the poor-rich gap in prevalence of severe underweight

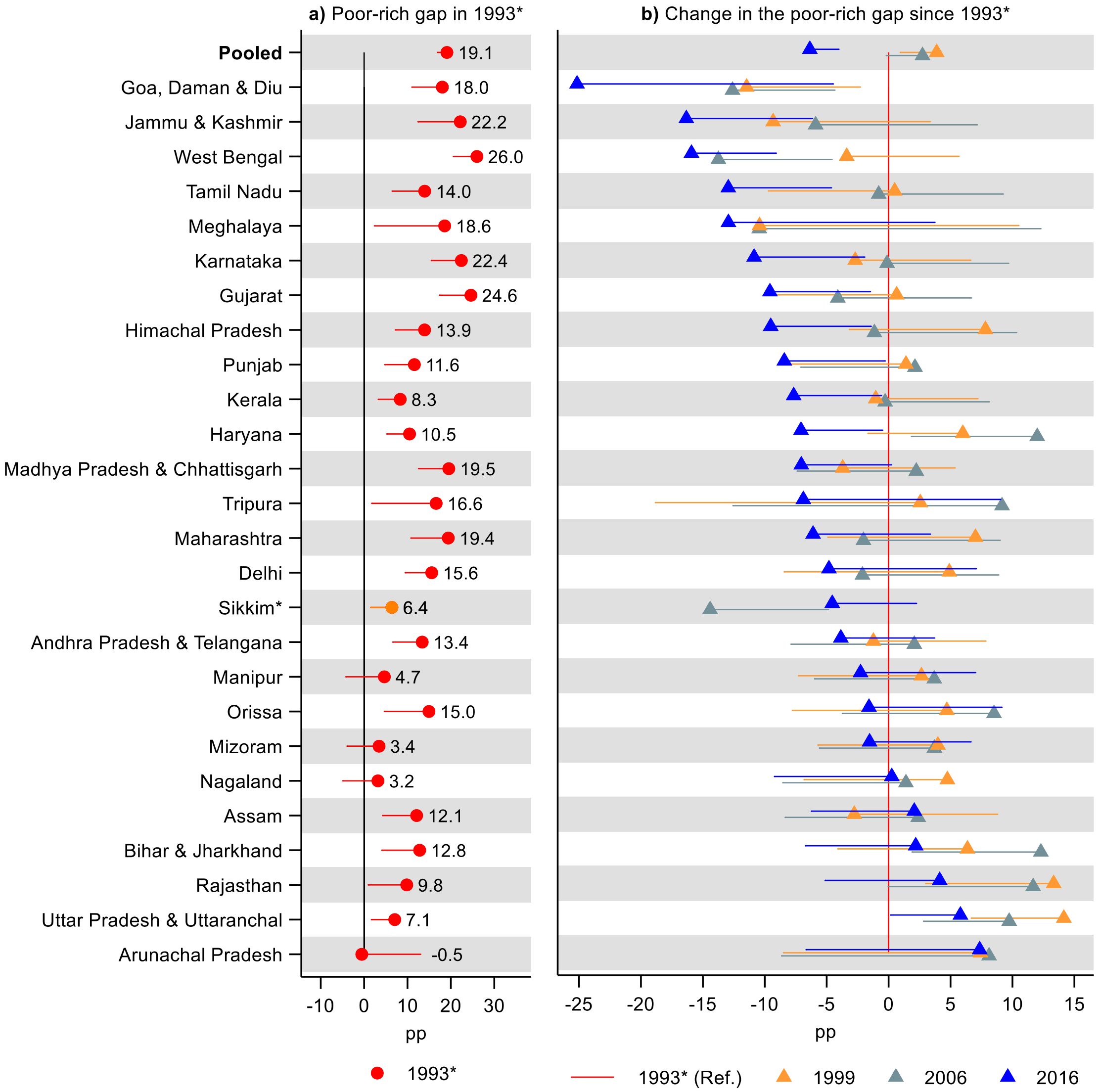

Notes: Percentage point (pp) differences are shown. In Panel a) a positive estimate indicates disadvantage for the poorest children, compared to the richest children, in 1993. A negative estimate in Panel b) indicates that the poor-rich gap (which usually shows a poor disadvantage in Panel a) has shrunk since 1993. The estimates were obtained from an interaction model (OLS) for each state: Panel a) shows the terms for the poorest wealth quintile and Panel b) shows the interaction terms (ie, between poorest quintile and year). The terms for year as well as all terms involving quintiles other than the poorest are excluded from the figure. Vertical lines (at 0) indicate no poor-rich difference in Panel a) and no change in rich-poor gap in Panel b). *Indicates states with no data for 1993: 1999 was used instead. 95% confidence bounds are shown. Only one confidence bound is shown to improve readability: an upper bound where the estimated difference was lower than 0 and a lower bound where the estimated difference was greater than zero. Estimates are weighted using sampling weights and confidence intervals were adjusted for clustering at the PSU-level.

Figure S44. Changes in the poor-rich gap in prevalence of severe stunting

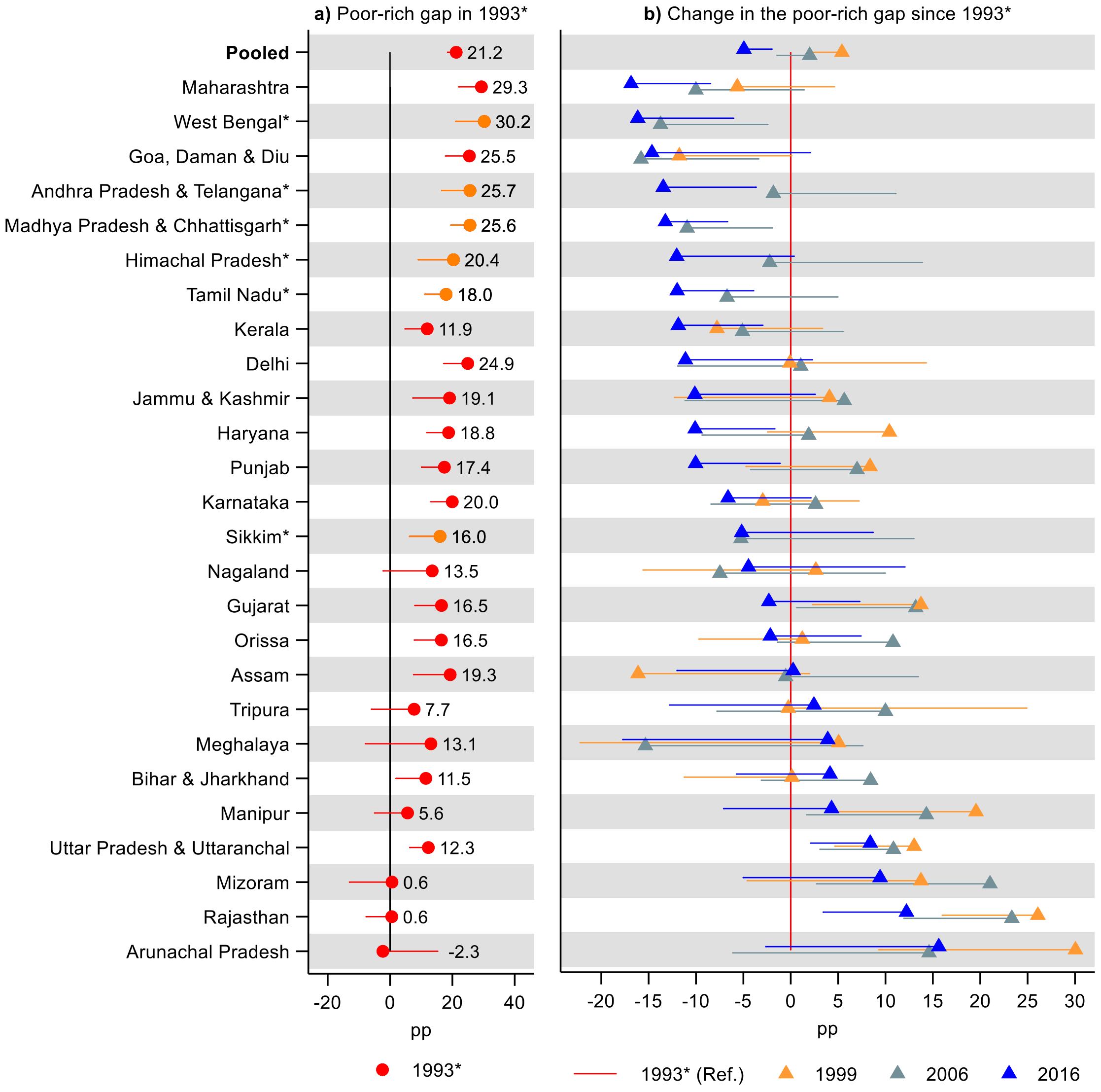

Notes: Percentage point (pp) differences are shown. In Panel a) a positive estimate indicates disadvantage for the poorest children, compared to the richest children, in 1993. A negative estimate in Panel b) indicates that the poor-rich gap (which usually shows a poor disadvantage in Panel a) has shrunk since 1993. The estimates were obtained from an interaction model (OLS) for each state: Panel a) shows the terms for the poorest wealth quintile and Panel b) shows the interaction terms (ie, between poorest quintile and year). The terms for year as well as all terms involving quintiles other than the poorest are excluded from the figure. Vertical lines (at 0) indicate no poor-rich difference in Panel a) and no change in rich-poor gap in Panel b). *Indicates states with no data for 1993: 1999 was used instead. 95% confidence bounds are shown. Only one confidence bound is shown to improve readability: an upper bound where the estimated difference was lower than 0 and a lower bound where the estimated difference was greater than zero. Estimates are weighted using sampling weights and confidence intervals were adjusted for clustering at the PSU-level.

Figure S45. Changes in the poor-rich gap in prevalence of severe wasting

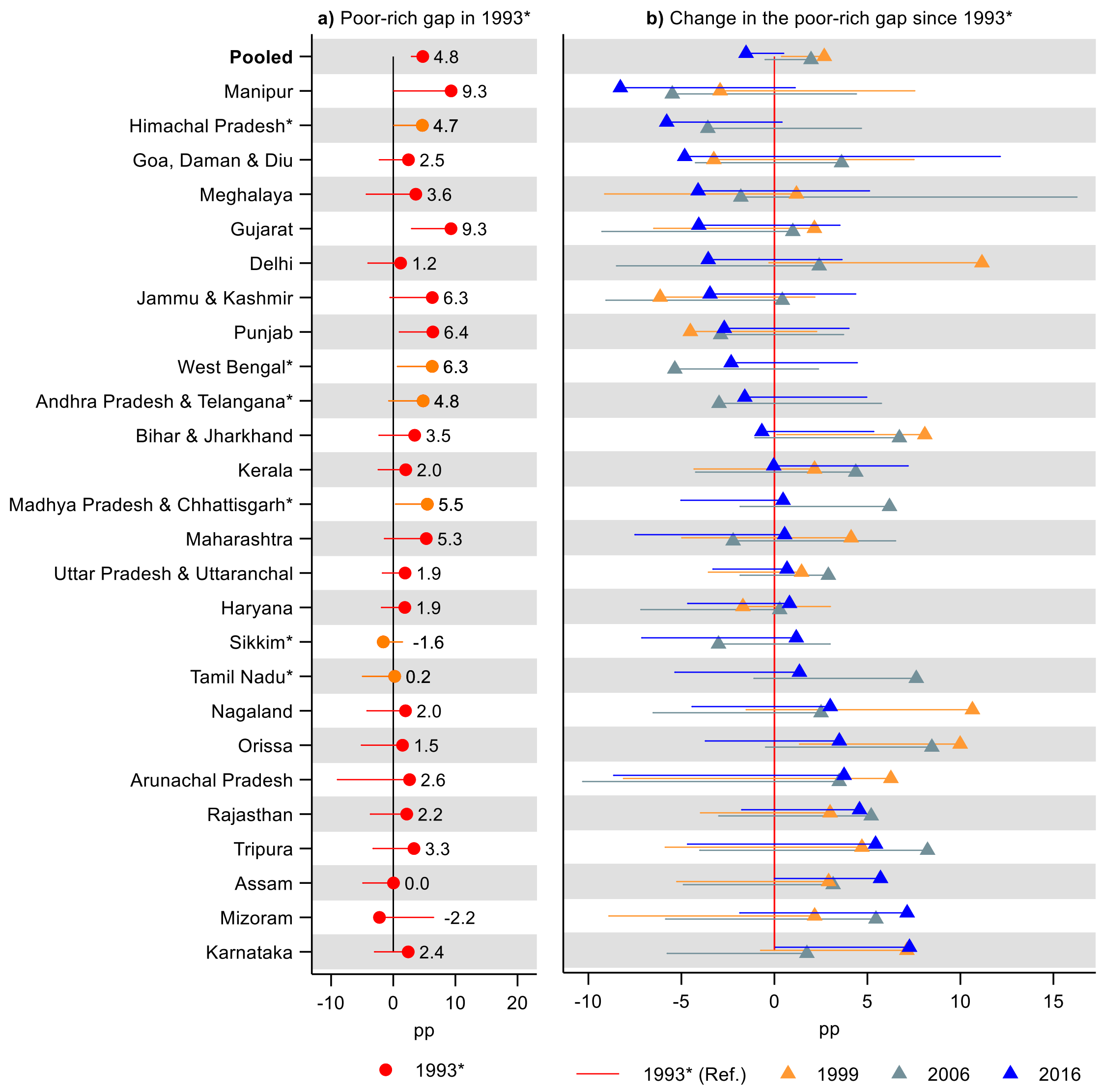

Notes: Percentage point (pp) differences are shown. In Panel a) a positive estimate indicates disadvantage for the poorest children, compared to the richest children, in 1993. A negative estimate in Panel b) indicates that the poor-rich gap (which usually shows a poor disadvantage in Panel a) has shrunk since 1993. The estimates were obtained from an interaction model (OLS) for each state: Panel a) shows the terms for the poorest wealth quintile and Panel b) shows the interaction terms (ie, between poorest quintile and year). The terms for year as well as all terms involving quintiles other than the poorest are excluded from the figure. Vertical lines (at 0) indicate no poor-rich difference in Panel a) and no change in rich-poor gap in Panel b). *Indicates states with no data for 1993: 1999 was used instead. 95% confidence bounds are shown. Only one confidence bound is shown to improve readability: an upper bound where the estimated difference was lower than 0 and a lower bound where the estimated difference was greater than zero. Estimates are weighted using sampling weights and confidence intervals were adjusted for clustering at the PSU-level.

Figure S46. Mean weight-for-age and average annual reduction (AAR) in weight-for-age between 1993 and 2016

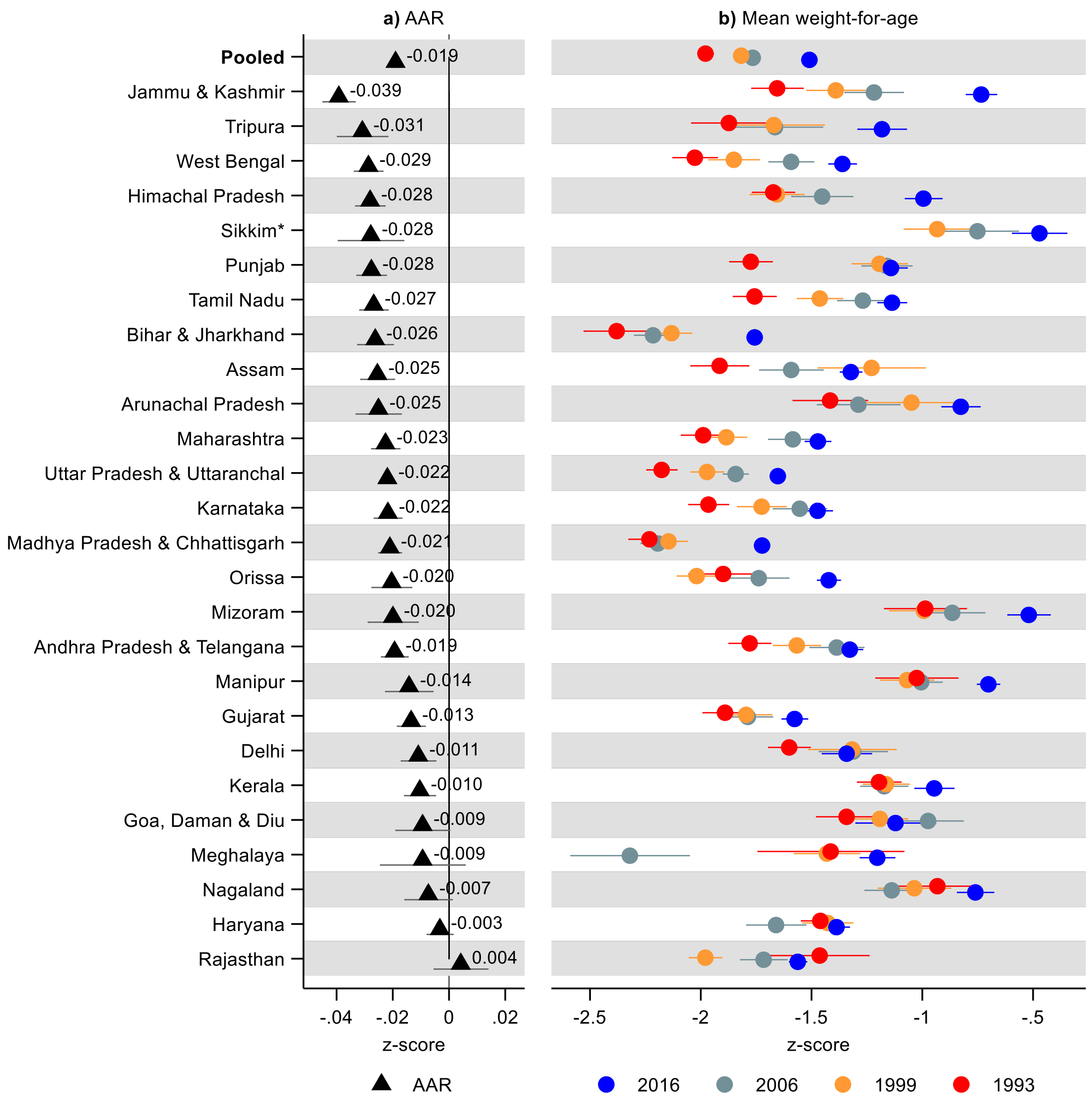

Notes: *Indicates states with no data for 1993: 1999 was used instead. Average annual reduction (AAR) shows average annual z-score reduction in mean weight-for-age in each state. 95% confidence intervals are shown. Estimates are weighted using sampling weights and confidence intervals were adjusted for clustering at the PSU-level.

Figure S47. Mean height-for-age and average annual reduction (AAR) in height-for-age between 1993 and 2016

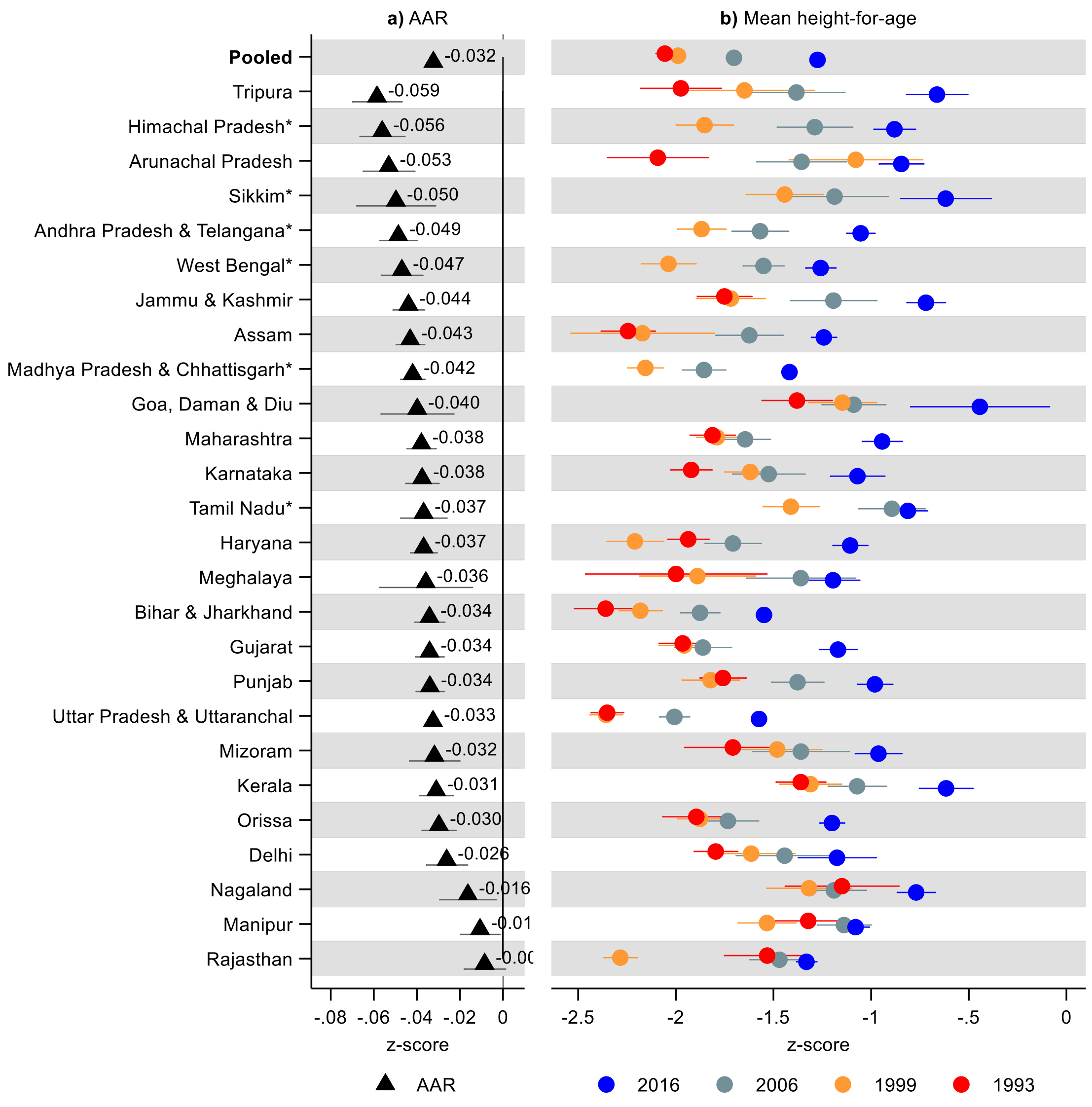

Notes: *Indicates states with no data for 1993: 1999 was used instead. Average annual reduction (AAR) shows average annual z-score reduction in mean height-for-age in each state. 95% confidence intervals are shown. Estimates are weighted using sampling weights and confidence intervals were adjusted for clustering at the PSU-level.

Figure S48. Mean weight-for-height and average annual reduction (AAR) in weight-for-height between 1993 and 2016

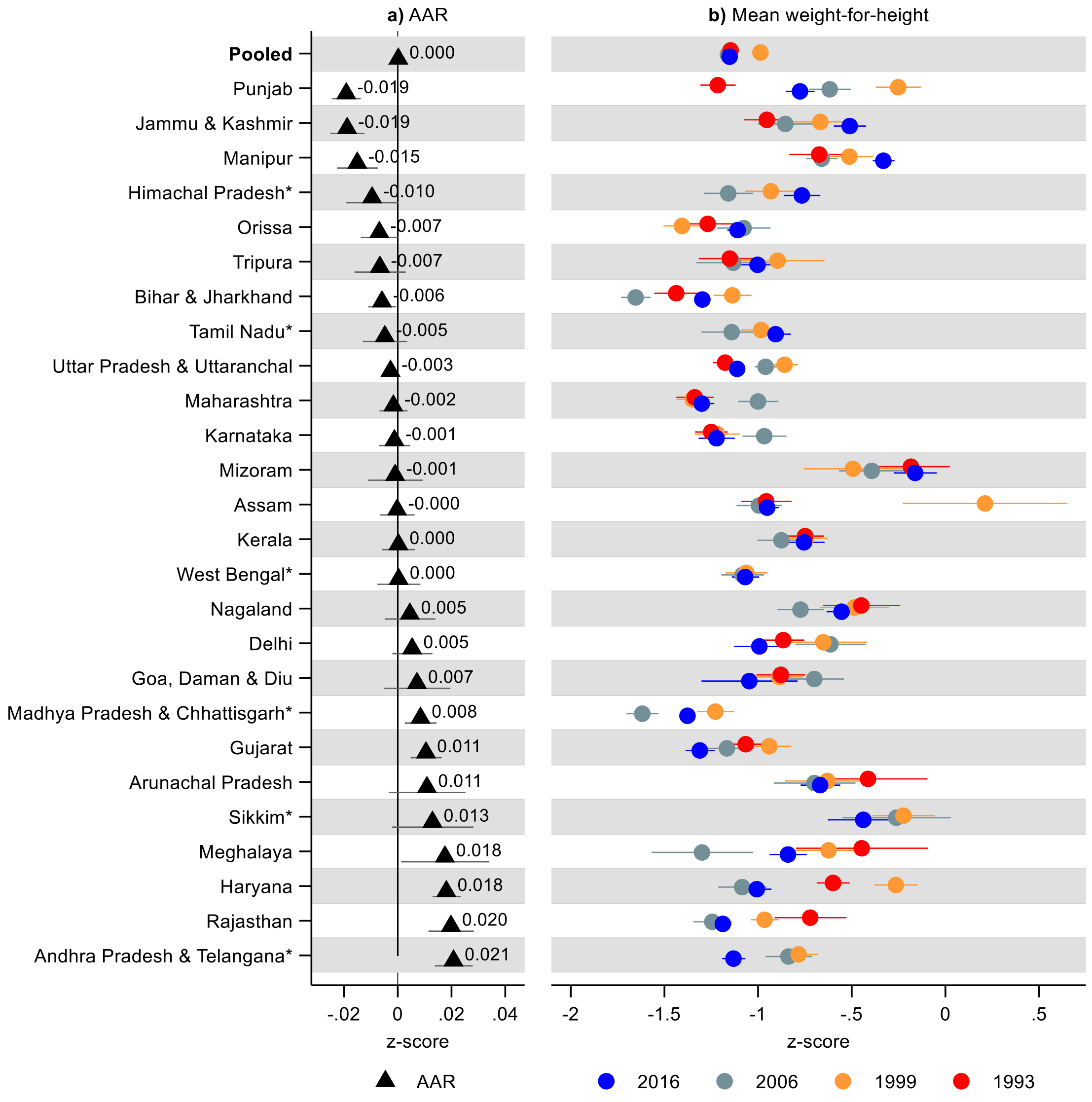

Notes: *Indicates states with no data for 1993: 1999 was used instead. Average annual reduction (AAR) shows average annual z-score reduction in mean weight-for-height in each state. 95% confidence intervals are shown. Estimates are weighted using sampling weights and confidence intervals were adjusted for clustering at the PSU-level.

Figure S49. Changes in the poor-rich gap in mean weight-for-age

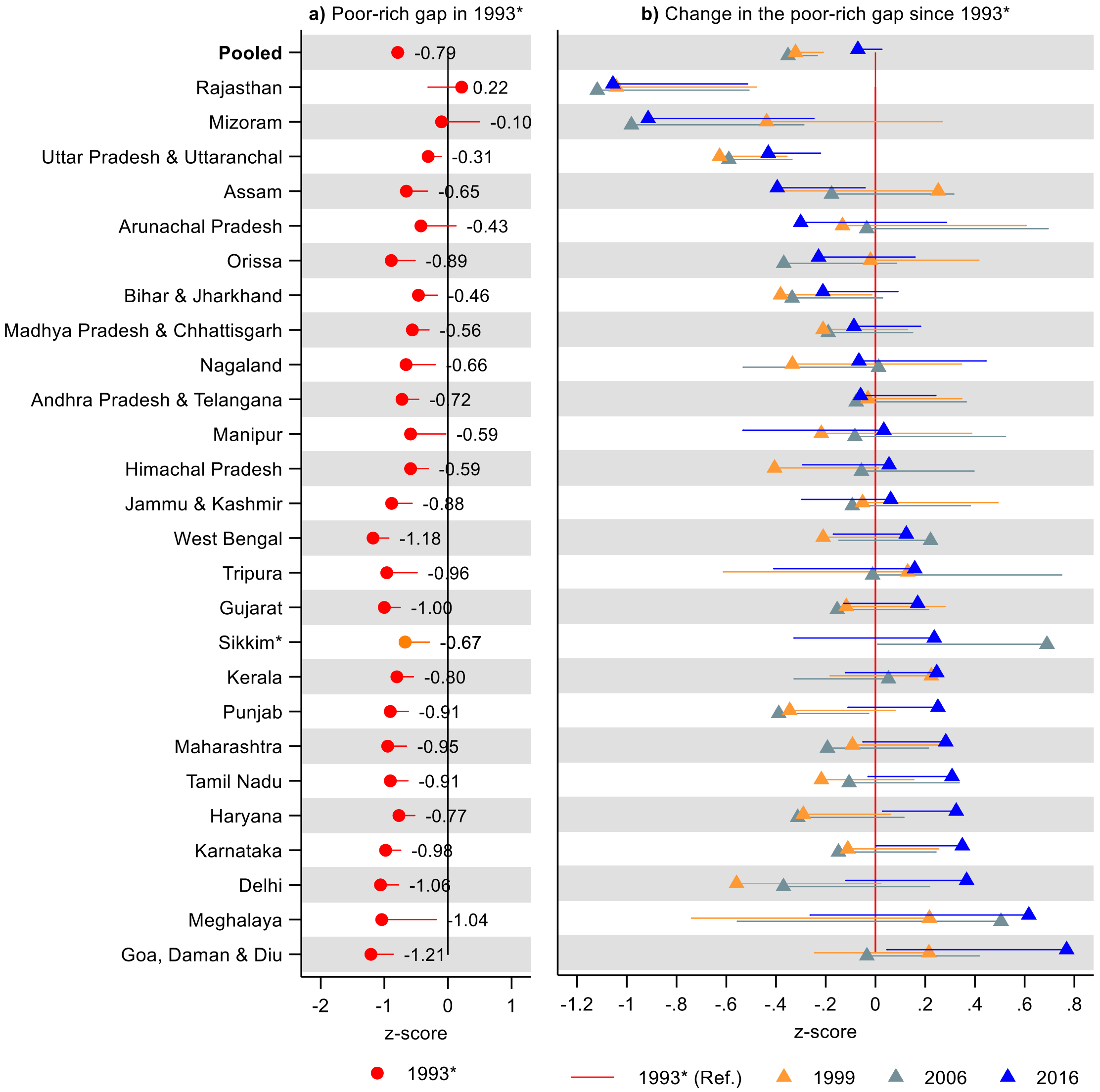

Notes: Differences in z-scores are shown. In Panel a) a negative estimate indicates disadvantage for the poorest children, compared to the richest children, in 1993. A positive estimate in Panel b) indicates that the poor-rich gap (which usually shows a poor disadvantage in Panel a) has shrunk since 1993. The estimates were obtained from an interaction model (OLS) for each state: Panel a) shows the terms for the poorest wealth quintile and Panel b) shows the interaction terms (ie, between poorest quintile and year). The terms for year as well as all terms involving quintiles other than the poorest are excluded from the figure. Vertical lines (at 0) indicate no poor-rich difference in Panel a) and no change in rich-poor gap in Panel b). *Indicates states with no data for 1993: 1999 was used instead. 95% confidence bounds are shown. Only one confidence bound is shown to improve readability: an upper bound where the estimated difference was lower than 0 and a lower bound where the estimated difference was greater than zero. Estimates are weighted using sampling weights and confidence intervals were adjusted for clustering at the PSU-level.

Figure S50. Changes in the poor-rich gap in mean height-for-age

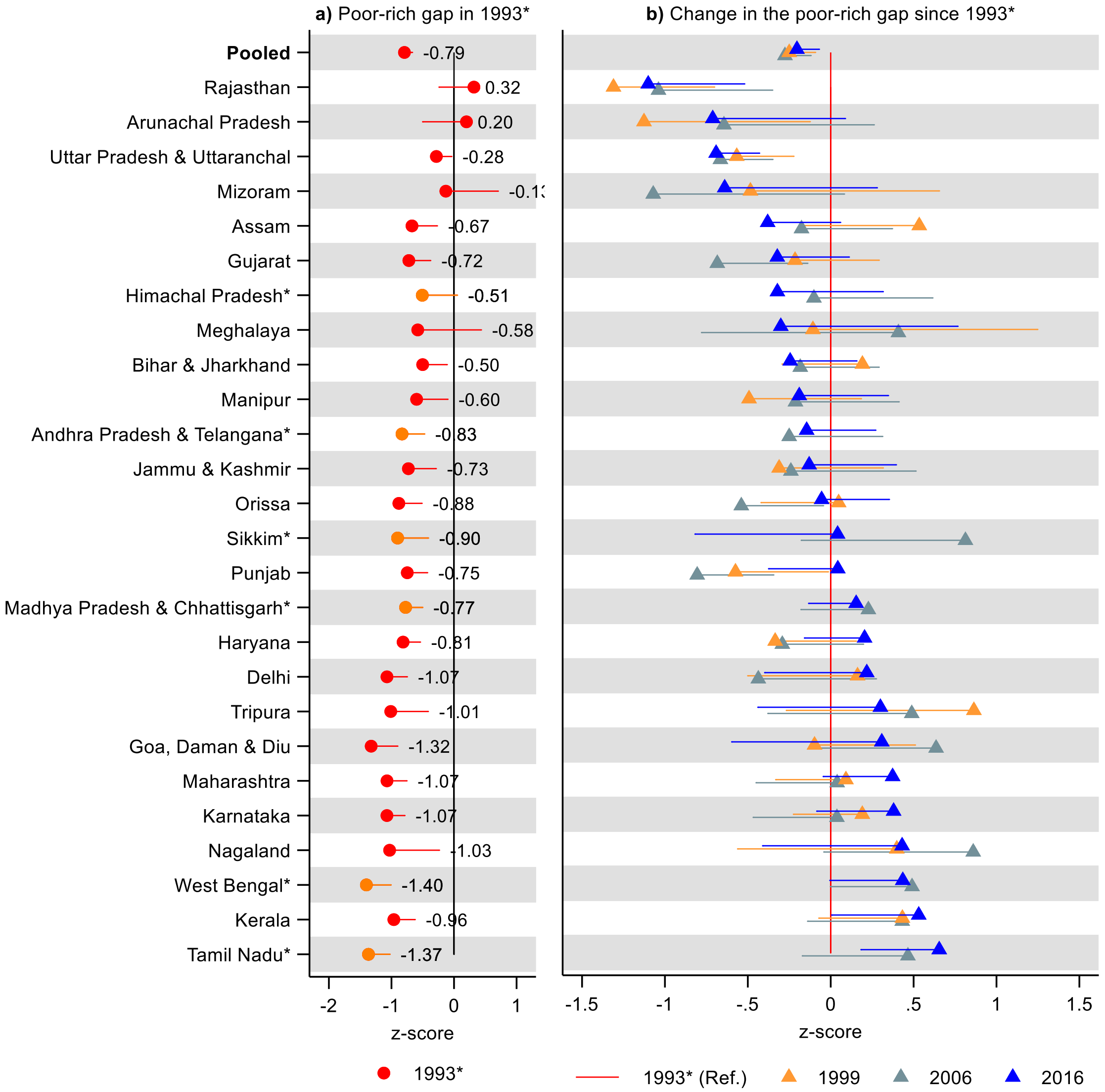

Notes: Differences in z-scores are shown. In Panel a) a negative estimate indicates disadvantage for the poorest children, compared to the richest children, in 1993. A negative positive in Panel b) indicates that the poor-rich gap (which usually shows a poor disadvantage in Panel a) has shrunk since 1993. The estimates were obtained from an interaction model (OLS) for each state: Panel a) shows the terms for the poorest wealth quintile and Panel b) shows the interaction terms (ie, between poorest quintile and year). The terms for year as well as all terms involving quintiles other than the poorest are excluded from the figure. Vertical lines (at 0) indicate no poor-rich difference in Panel a) and no change in rich-poor gap in Panel b). *Indicates states with no data for 1993: 1999 was used instead. 95% confidence bounds are shown. Only one confidence bound is shown to improve readability: an upper bound where the estimated difference was lower than 0 and a lower bound where the estimated difference was greater than zero. Estimates are weighted using sampling weights and confidence intervals were adjusted for clustering at the PSU-level.

Figure S51. Changes in the poor-rich gap in mean weight-for-height

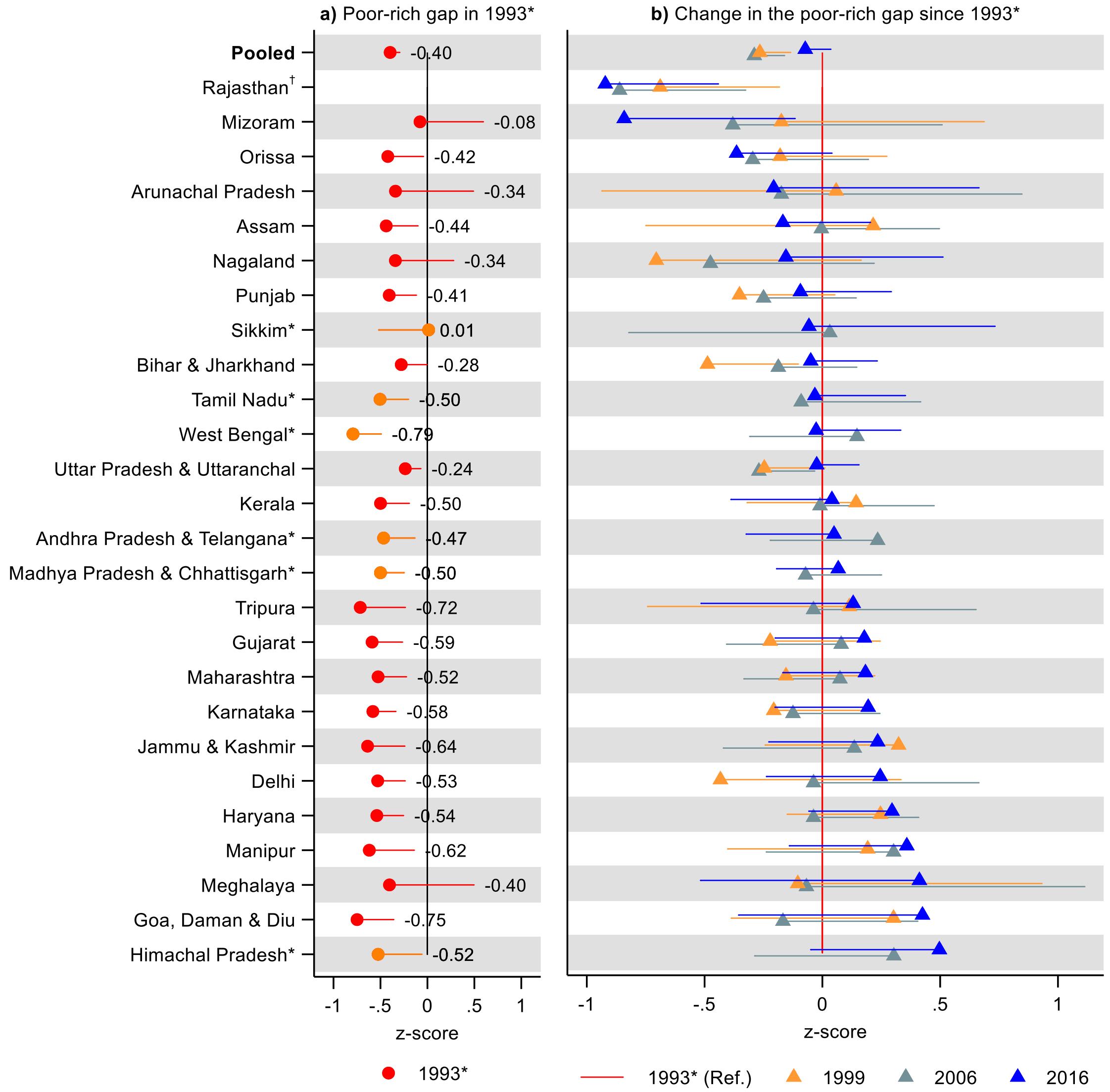

Notes: Differences in z-scores are shown. In Panel a) a negative estimate indicates disadvantage for the poorest children, compared to the richest children, in 1993. A positive estimate in Panel b) indicates that the poor-rich gap (which usually shows a poor disadvantage in Panel a) has shrunk since 1993. The estimates were obtained from an interaction model (OLS) for each state: Panel a) shows the terms for the poorest wealth quintile and Panel b) shows the interaction terms (ie, between poorest quintile and year). The terms for year as well as all terms involving quintiles other than the poorest are excluded from the figure. Vertical lines (at 0) indicate no poor-rich difference in Panel a) and no change in rich-poor gap in Panel b). *Indicates states with no data for 1993: 1999 was used instead. †Extreme point estimates (more than 3 times the interquartile range from the median) were omitted to improve readability in Panel a). 95% confidence bounds are shown. Only one confidence bound is shown to improve readability: an upper bound where the estimated difference was lower than 0 and a lower bound where the estimated difference was greater than zero. Estimates are weighted using sampling weights and confidence intervals were adjusted for clustering at the PSU-level.

Table S1. Full sample (of children 0–36 months) and analytical samples for stunting, wasting and underweight, by state and year

State	Year	Full	Missing Underweight	Stunting	Wasting
Pooled	1993	35,055	29,257[83%]	21,612[62%]	16,863[48%]
Pooled	1999	27,621	26,888[97%]	25,915[94%]	20,011[72%]
Pooled	2006	29,485	26,728[91%]	25,844[88%]	21,255[72%]
Pooled	2016	148,117	143,293[97%]	140,373[95%]	114,999[78%]
Jammu & Kashmir	1993	1,102	913[83%]	891[81%]	665[60%]
Jammu & Kashmir	1999	895	885[99%]	826[92%]	642[72%]
Jammu & Kashmir	2006	697	632[91%]	622[89%]	528[76%]
Jammu & Kashmir	2016	4,794	4,633[97%]	4,506[94%]	3,905[81%]
Himachal Pradesh	1993	1,044	1,009[97%]	0[0%]	0[0%]
Himachal Pradesh	1999	828	821[99%]	807[97%]	618[75%]
Himachal Pradesh	2006	581	556[96%]	534[92%]	462[80%]
Himachal Pradesh	2016	1,688	1,599[95%]	1,563[93%]	1,326[79%]
Punjab	1993	1,130	975[86%]	978[87%]	756[67%]
Punjab	1999	795	789[99%]	759[95%]	583[73%]
Punjab	2006	732	695[95%]	693[95%]	556[76%]
Punjab	2016	3,044	3,001[99%]	2,950[97%]	2,476[81%]
Haryana	1993	1,295	1,127[87%]	1,115[86%]	856[66%]
Haryana	1999	953	949[100%]	894[94%]	680[71%]
Haryana	2006	721	695[96%]	686[95%]	569[79%]
Haryana	2016	4,606	4,475[97%]	4,362[95%]	3,500[76%]
Delhi	1993	1,359	1,191[88%]	1,165[86%]	949[70%]
Delhi	1999	687	658[96%]	631[92%]	514[75%]
Delhi	2006	714	464[65%]	446[62%]	368[52%]
Delhi	2016	915	753[82%]	739[81%]	626[68%]
Rajasthan	1993	2,082	1,599[77%]	1,473[71%]	1,149[55%]
Rajasthan	1999	2,573	2,466[96%]	2,384[93%]	1,766[69%]
Rajasthan	2006	1,123	1,107[99%]	1,076[96%]	862[77%]
Rajasthan	2016	9,742	9,516[98%]	9,411[97%]	7,537[77%]
Uttar Pradesh & Uttaranchal	1993	5,534	4,638[84%]	4,475[81%]	3,384[61%]
Uttar Pradesh & Uttaranchal	1999	2,636	2,526[96%]	2,485[94%]	1,831[69%]
Uttar Pradesh & Uttaranchal	2006	4,683	4,108[88%]	3,973[85%]	3,164[68%]
Uttar Pradesh & Uttaranchal	2016	27,032	26,448[98%]	26,088[97%]	21,101[78%]
Bihar & Jharkhand	1993	2,478	2,078[84%]	1,939[78%]	1,457[59%]
Bihar & Jharkhand	1999	2,468	2,362[96%]	2,188[89%]	1,581[64%]
Bihar & Jharkhand	2006	2,220	2,090[94%]	2,056[93%]	1,652[74%]
Bihar & Jharkhand	2016	21,725	21,192[98%]	20,830[96%]	16,892[78%]
Sikkim	1999	386	381[99%]	372[96%]	289[75%]
Sikkim	2006	379	349[92%]	306[81%]	260[69%]
Sikkim	2016	619	613[99%]	595[96%]	499[81%]
Arunachal Pradesh	1993	471	315[67%]	278[59%]	232[49%]
Arunachal Pradesh	1999	383	378[99%]	372[97%]	314[82%]
Arunachal Pradesh	2006	472	455[96%]	433[92%]	368[78%]
Arunachal Pradesh	2016	2,717	2,421[89%]	2,288[84%]	1,955[72%]
Nagaland	1993	516	395[77%]	379[73%]	319[62%]
Nagaland	1999	408	396[97%]	394[97%]	307[75%]
Nagaland	2006	1,250	1,171[94%]	1,086[87%]	919[74%]
Nagaland	2016	2,692	2,485[92%]	2,405[89%]	2,059[76%]
Manipur	1993	424	363[86%]	352[83%]	272[64%]
Manipur	1999	617	615[100%]	609[99%]	503[82%]
Manipur	2006	1,166	1,075[92%]	1,058[91%]	897[77%]
Manipur	2016	3,395	3,368[99%]	3,329[98%]	2,803[83%]
Mizoram	1993	346	294[85%]	277[80%]	220[64%]
Mizoram	1999	447	440[98%]	435[97%]	359[80%]
Mizoram	2006	500	478[96%]	461[92%]	413[83%]
Mizoram	2016	2,780	2,701[97%]	2,647[95%]	2,313[83%]

State	Year	Full	Missing Underweight	Stunting	Wasting
Tripura	1993	380	254[67%]	229[60%]	179[47%]
Tripura	1999	273	265[97%]	240[88%]	201[74%]
Tripura	2006	377	359[95%]	343[91%]	273[72%]
Tripura	2016	773	745[96%]	732[95%]	614[79%]
Meghalaya	1993	495	351[71%]	296[60%]	240[48%]
Meghalaya	1999	449	439[98%]	426[95%]	320[71%]
Meghalaya	2006	641	509[79%]	478[75%]	291[45%]
Meghalaya	2016	2,544	2,456[97%]	2,382[94%]	1,944[76%]
Assam	1993	1,314	1,067[81%]	1,029[78%]	820[62%]
Assam	1999	835	804[96%]	700[84%]	496[59%]
Assam	2006	857	793[93%]	754[88%]	606[71%]
Assam	2016	5,988	5,766[96%]	5,534[92%]	4,619[77%]
West Bengal	1993	1,592	1,338[84%]	0[0%]	0[0%]
West Bengal	1999	1,103	1,090[99%]	1,076[98%]	838[76%]
West Bengal	2006	1,321	1,234[93%]	1,204[91%]	995[75%]
West Bengal	2016	3,113	3,005[97%]	2,967[95%]	2,484[80%]
Orissa	1993	1,546	1,212[78%]	1,180[76%]	937[61%]
Orissa	1999	1,343	1,319[98%]	1,299[97%]	1,051[78%]
Orissa	2006	1,014	983[97%]	958[94%]	758[75%]
Orissa	2016	6,421	6,190[96%]	6,070[95%]	5,086[79%]
Madhya Pradesh & Chhattisgarh	1993	2,705	2,306[85%]	0[0%]	0[0%]
Madhya Pradesh & Chhattisgarh	1999	2,466	2,381[97%]	2,251[91%]	1,676[68%]
Madhya Pradesh & Chhattisgarh	2006	2,546	2,482[97%]	2,462[97%]	1,949[77%]
Madhya Pradesh & Chhattisgarh	2016	19,373	18,949[98%]	18,629[96%]	14,676[76%]
Gujarat	1993	1,417	1,211[85%]	1,151[81%]	908[64%]
Gujarat	1999	1,141	1,114[98%]	1,078[94%]	814[71%]
Gujarat	2006	875	836[96%]	821[94%]	667[76%]
Gujarat	2016	4,383	4,149[95%]	4,049[92%]	3,295[75%]
Maharashtra	1993	1,527	1,236[81%]	1,221[80%]	954[62%]
Maharashtra	1999	1,636	1,579[97%]	1,572[96%]	1,271[78%]
Maharashtra	2006	1,806	1,519[84%]	1,411[78%]	1,211[67%]
Maharashtra	2016	5,480	5,214[95%]	5,077[93%]	4,184[76%]
Andhra Pradesh & Telangana	1993	1,341	1,207[90%]	0[0%]	0[0%]
Andhra Pradesh & Telangana	1999	1,002	987[99%]	964[96%]	742[74%]
Andhra Pradesh & Telangana	2006	1,302	1,140[88%]	1,096[84%]	947[73%]
Andhra Pradesh & Telangana	2016	3,247	2,957[91%]	2,912[90%]	2,502[77%]
Karnataka	1993	1,643	1,316[80%]	1,306[79%]	1,036[63%]
Karnataka	1999	1,113	1,082[97%]	1,070[96%]	858[77%]
Karnataka	2006	1,263	962[76%]	918[73%]	801[63%]
Karnataka	2016	4,487	4,286[96%]	4,131[92%]	3,415[76%]
Goa, Daman & Diu	1993	875	795[91%]	780[89%]	649[74%]
Goa, Daman & Diu	1999	304	302[99%]	303[100%]	267[88%]
Goa, Daman & Diu	2006	604	491[81%]	476[79%]	432[72%]
Goa, Daman & Diu	2016	500	475[95%]	457[91%]	385[77%]
Kerala	1993	1,205	1,102[91%]	1,098[91%]	881[73%]
Kerala	1999	636	635[100%]	584[92%]	492[77%]
Kerala	2006	613	578[94%]	566[92%]	509[83%]
Kerala	2016	1,464	1,436[98%]	1,384[95%]	1,164[80%]
Tamil Nadu	1993	1,234	965[78%]	0[0%]	0[0%]
Tamil Nadu	1999	1,244	1,225[98%]	1,196[96%]	998[80%]
Tamil Nadu	2006	1,028	967[94%]	927[90%]	798[78%]
Tamil Nadu	2016	4,595	4,460[97%]	4,336[94%]	3,639[79%]

Table S2. Comparing household wealth between included children and children with missing underweight, stunting, or wasting, by state and year

		Wealth z-score						Age (days)					
State	Year	Included		Missing		Difference		Included		Missing		Difference	
Pooled	1993	-0.05	[-0.06, -0.03]	-0.17	[-0.18, -0.15]	-0.12	[-0.14, -0.10]	638.98	[634.12, 643.83]	437.37	[431.65, 443.09]	-201.60	[-209.10, -194.11]
Pooled	1999	-0.04	[-0.06, -0.03]	-0.24	[-0.26, -0.22]	-0.20	[-0.22, -0.17]	630.49	[626.18, 634.80]	252.92	[246.16, 259.69]	-377.57	[-385.59, -369.55]
Pooled	2006	-0.14	[-0.16, -0.13]	-0.21	[-0.24, -0.19]	-0.07	[-0.10, -0.04]	648.50	[643.80, 653.20]	294.74	[286.59, 302.90]	-353.76	[-363.17, -344.35]
Pooled	2016	-0.16	[-0.16, -0.15]	-0.18	[-0.20, -0.17]	-0.03	[-0.05, -0.01]	644.92	[642.60, 647.25]	257.73	[252.50, 262.96]	-387.19	[-392.92, -381.47]
Jammu & Kashmir	1993	-0.05	[-0.06, -0.03]	-0.17	[-0.18, -0.15]	-0.12	[-0.14, -0.10]	638.98	[634.12, 643.83]	437.37	[431.65, 443.09]	-201.60	[-209.10, -194.11]
Jammu & Kashmir	1999	-0.04	[-0.06, -0.03]	-0.24	[-0.26, -0.22]	-0.20	[-0.22, -0.17]	630.49	[626.18, 634.80]	252.92	[246.16, 259.69]	-377.57	[-385.59, -369.55]
Jammu & Kashmir	2006	-0.14	[-0.16, -0.13]	-0.21	[-0.24, -0.19]	-0.07	[-0.10, -0.04]	648.50	[643.80, 653.20]	294.74	[286.59, 302.90]	-353.76	[-363.17, -344.35]
Jammu & Kashmir	2016	-0.16	[-0.16, -0.15]	-0.18	[-0.20, -0.17]	-0.03	[-0.05, -0.01]	644.92	[642.60, 647.25]	257.73	[252.50, 262.96]	-387.19	[-392.92, -381.47]
Himachal Pradesh	1993	-0.05	[-0.06, -0.03]	-0.17	[-0.18, -0.15]	-0.12	[-0.14, -0.10]	638.98	[634.12, 643.83]	437.37	[431.65, 443.09]	-201.60	[-209.10, -194.11]
Himachal Pradesh	1999	-0.04	[-0.06, -0.03]	-0.24	[-0.26, -0.22]	-0.20	[-0.22, -0.17]	630.49	[626.18, 634.80]	252.92	[246.16, 259.69]	-377.57	[-385.59, -369.55]
Himachal Pradesh	2006	-0.14	[-0.16, -0.13]	-0.21	[-0.24, -0.19]	-0.07	[-0.10, -0.04]	648.50	[643.80, 653.20]	294.74	[286.59, 302.90]	-353.76	[-363.17, -344.35]
Himachal Pradesh	2016	-0.16	[-0.16, -0.15]	-0.18	[-0.20, -0.17]	-0.03	[-0.05, -0.01]	644.92	[642.60, 647.25]	257.73	[252.50, 262.96]	-387.19	[-392.92, -381.47]
Punjab	1993	-0.05	[-0.06, -0.03]	-0.17	[-0.18, -0.15]	-0.12	[-0.14, -0.10]	638.98	[634.12, 643.83]	437.37	[431.65, 443.09]	-201.60	[-209.10, -194.11]
Punjab	1999	-0.04	[-0.06, -0.03]	-0.24	[-0.26, -0.22]	-0.20	[-0.22, -0.17]	630.49	[626.18, 634.80]	252.92	[246.16, 259.69]	-377.57	[-385.59, -369.55]
Punjab	2006	-0.14	[-0.16, -0.13]	-0.21	[-0.24, -0.19]	-0.07	[-0.10, -0.04]	648.50	[643.80, 653.20]	294.74	[286.59, 302.90]	-353.76	[-363.17, -344.35]
Punjab	2016	-0.16	[-0.16, -0.15]	-0.18	[-0.20, -0.17]	-0.03	[-0.05, -0.01]	644.92	[642.60, 647.25]	257.73	[252.50, 262.96]	-387.19	[-392.92, -381.47]
Haryana	1993	-0.05	[-0.06, -0.03]	-0.17	[-0.18, -0.15]	-0.12	[-0.14, -0.10]	638.98	[634.12, 643.83]	437.37	[431.65, 443.09]	-201.60	[-209.10, -194.11]
Haryana	1999	-0.04	[-0.06, -0.03]	-0.24	[-0.26, -0.22]	-0.20	[-0.22, -0.17]	630.49	[626.18, 634.80]	252.92	[246.16, 259.69]	-377.57	[-385.59, -369.55]
Haryana	2006	-0.14	[-0.16, -0.13]	-0.21	[-0.24, -0.19]	-0.07	[-0.10, -0.04]	648.50	[643.80, 653.20]	294.74	[286.59, 302.90]	-353.76	[-363.17, -344.35]
Haryana	2016	-0.16	[-0.16, -0.15]	-0.18	[-0.20, -0.17]	-0.03	[-0.05, -0.01]	644.92	[642.60, 647.25]	257.73	[252.50, 262.96]	-387.19	[-392.92, -381.47]
Delhi	1993	-0.05	[-0.06, -0.03]	-0.17	[-0.18, -0.15]	-0.12	[-0.14, -0.10]	638.98	[634.12, 643.83]	437.37	[431.65, 443.09]	-201.60	[-209.10, -194.11]
Delhi	1999	-0.04	[-0.06, -0.03]	-0.24	[-0.26, -0.22]	-0.20	[-0.22, -0.17]	630.49	[626.18, 634.80]	252.92	[246.16, 259.69]	-377.57	[-385.59, -369.55]
Delhi	2006	-0.14	[-0.16, -0.13]	-0.21	[-0.24, -0.19]	-0.07	[-0.10, -0.04]	648.50	[643.80, 653.20]	294.74	[286.59, 302.90]	-353.76	[-363.17, -344.35]
Delhi	2016	-0.16	[-0.16, -0.15]	-0.18	[-0.20, -0.17]	-0.03	[-0.05, -0.01]	644.92	[642.60, 647.25]	257.73	[252.50, 262.96]	-387.19	[-392.92, -381.47]
Rajasthan	1993	-0.05	[-0.06, -0.03]	-0.17	[-0.18, -0.15]	-0.12	[-0.14, -0.10]	638.98	[634.12, 643.83]	437.37	[431.65, 443.09]	-201.60	[-209.10, -194.11]
Rajasthan	1999	-0.04	[-0.06, -0.03]	-0.24	[-0.26, -0.22]	-0.20	[-0.22, -0.17]	630.49	[626.18, 634.80]	252.92	[246.16, 259.69]	-377.57	[-385.59, -369.55]
Rajasthan	2006	-0.14	[-0.16, -0.13]	-0.21	[-0.24, -0.19]	-0.07	[-0.10, -0.04]	648.50	[643.80, 653.20]	294.74	[286.59, 302.90]	-353.76	[-363.17, -344.35]
Rajasthan	2016	-0.16	[-0.16, -0.15]	-0.18	[-0.20, -0.17]	-0.03	[-0.05, -0.01]	644.92	[642.60, 647.25]	257.73	[252.50, 262.96]	-387.19	[-392.92, -381.47]
Uttar Pradesh & Uttaranchal	1993	-0.05	[-0.06, -0.03]	-0.17	[-0.18, -0.15]	-0.12	[-0.14, -0.10]	638.98	[634.12, 643.83]	437.37	[431.65, 443.09]	-201.60	[-209.10, -194.11]
Uttar Pradesh & Uttaranchal	1999	-0.04	[-0.06, -0.03]	-0.24	[-0.26, -0.22]	-0.20	[-0.22, -0.17]	630.49	[626.18, 634.80]	252.92	[246.16, 259.69]	-377.57	[-385.59, -369.55]
Uttar Pradesh & Uttaranchal	2006	-0.14	[-0.16, -0.13]	-0.21	[-0.24, -0.19]	-0.07	[-0.10, -0.04]	648.50	[643.80, 653.20]	294.74	[286.59, 302.90]	-353.76	[-363.17, -344.35]
Uttar Pradesh & Uttaranchal	2016	-0.16	[-0.16, -0.15]	-0.18	[-0.20, -0.17]	-0.03	[-0.05, -0.01]	644.92	[642.60, 647.25]	257.73	[252.50, 262.96]	-387.19	[-392.92, -381.47]
Bihar & Jharkhand	1993	-0.05	[-0.06, -0.03]	-0.17	[-0.18, -0.15]	-0.12	[-0.14, -0.10]	638.98	[634.12, 643.83]	437.37	[431.65, 443.09]	-201.60	[-209.10, -194.11]
Bihar & Jharkhand	1999	-0.04	[-0.06, -0.03]	-0.24	[-0.26, -0.22]	-0.20	[-0.22, -0.17]	630.49	[626.18, 634.80]	252.92	[246.16, 259.69]	-377.57	[-385.59, -369.55]
Bihar & Jharkhand	2006	-0.14	[-0.16, -0.13]	-0.21	[-0.24, -0.19]	-0.07	[-0.10, -0.04]	648.50	[643.80, 653.20]	294.74	[286.59, 302.90]	-353.76	[-363.17, -344.35]
Bihar & Jharkhand	2016	-0.16	[-0.16, -0.15]	-0.18	[-0.20, -0.17]	-0.03	[-0.05, -0.01]	644.92	[642.60, 647.25]	257.73	[252.50, 262.96]	-387.19	[-392.92, -381.47]
Sikkim	1999	-0.04	[-0.06, -0.03]	-0.24	[-0.26, -0.22]	-0.20	[-0.22, -0.17]	630.49	[626.18, 634.80]	252.92	[246.16, 259.69]	-377.57	[-385.59, -369.55]
Sikkim	2006	-0.14	[-0.16, -0.13]	-0.21	[-0.24, -0.19]	-0.07	[-0.10, -0.04]	648.50	[643.80, 653.20]	294.74	[286.59, 302.90]	-353.76	[-363.17, -344.35]
Sikkim	2016	-0.16	[-0.16, -0.15]	-0.18	[-0.20, -0.17]	-0.03	[-0.05, -0.01]	644.92	[642.60, 647.25]	257.73	[252.50, 262.96]	-387.19	[-392.92, -381.47]
Arunachal Pradesh	1993	-0.05	[-0.06, -0.03]	-0.17	[-0.18, -0.15]	-0.12	[-0.14, -0.10]	638.98	[634.12, 643.83]	437.37	[431.65, 443.09]	-201.60	[-209.10, -194.11]
Arunachal Pradesh	1999	-0.04	[-0.06, -0.03]	-0.24	[-0.26, -0.22]	-0.20	[-0.22, -0.17]	630.49	[626.18, 634.80]	252.92	[246.16, 259.69]	-377.57	[-385.59, -369.55]
Arunachal Pradesh	2006	-0.14	[-0.16, -0.13]	-0.21	[-0.24, -0.19]	-0.07	[-0.10, -0.04]	648.50	[643.80, 653.20]	294.74	[286.59, 302.90]	-353.76	[-363.17, -344.35]
Arunachal Pradesh	2016	-0.16	[-0.16, -0.15]	-0.18	[-0.20, -0.17]	-0.03	[-0.05, -0.01]	644.92	[642.60, 647.25]	257.73	[252.50, 262.96]	-387.19	[-392.92, -381.47]

		Wealth z-score						Age (days)					
State	Year	Included		Missing		Difference		Included		Missing		Difference	
Nagaland	1993	-0.05	[-0.06, -0.03]	-0.17	[-0.18, -0.15]	-0.12	[-0.14, -0.10]	638.98	[634.12, 643.83]	437.37	[431.65, 443.09]	-201.60	[-209.10, -194.11]
Nagaland	1999	-0.04	[-0.06, -0.03]	-0.24	[-0.26, -0.22]	-0.20	[-0.22, -0.17]	630.49	[626.18, 634.80]	252.92	[246.16, 259.69]	-377.57	[-385.59, -369.55]
Nagaland	2006	-0.14	[-0.16, -0.13]	-0.21	[-0.24, -0.19]	-0.07	[-0.10, -0.04]	648.50	[643.80, 653.20]	294.74	[286.59, 302.90]	-353.76	[-363.17, -344.35]
Nagaland	2016	-0.16	[-0.16, -0.15]	-0.18	[-0.20, -0.17]	-0.03	[-0.05, -0.01]	644.92	[642.60, 647.25]	257.73	[252.50, 262.96]	-387.19	[-392.92, -381.47]
Manipur	1993	-0.05	[-0.06, -0.03]	-0.17	[-0.18, -0.15]	-0.12	[-0.14, -0.10]	638.98	[634.12, 643.83]	437.37	[431.65, 443.09]	-201.60	[-209.10, -194.11]
Manipur	1999	-0.04	[-0.06, -0.03]	-0.24	[-0.26, -0.22]	-0.20	[-0.22, -0.17]	630.49	[626.18, 634.80]	252.92	[246.16, 259.69]	-377.57	[-385.59, -369.55]
Manipur	2006	-0.14	[-0.16, -0.13]	-0.21	[-0.24, -0.19]	-0.07	[-0.10, -0.04]	648.50	[643.80, 653.20]	294.74	[286.59, 302.90]	-353.76	[-363.17, -344.35]
Manipur	2016	-0.16	[-0.16, -0.15]	-0.18	[-0.20, -0.17]	-0.03	[-0.05, -0.01]	644.92	[642.60, 647.25]	257.73	[252.50, 262.96]	-387.19	[-392.92, -381.47]
Mizoram	1993	-0.05	[-0.06, -0.03]	-0.17	[-0.18, -0.15]	-0.12	[-0.14, -0.10]	638.98	[634.12, 643.83]	437.37	[431.65, 443.09]	-201.60	[-209.10, -194.11]
Mizoram	1999	-0.04	[-0.06, -0.03]	-0.24	[-0.26, -0.22]	-0.20	[-0.22, -0.17]	630.49	[626.18, 634.80]	252.92	[246.16, 259.69]	-377.57	[-385.59, -369.55]
Mizoram	2006	-0.14	[-0.16, -0.13]	-0.21	[-0.24, -0.19]	-0.07	[-0.10, -0.04]	648.50	[643.80, 653.20]	294.74	[286.59, 302.90]	-353.76	[-363.17, -344.35]
Mizoram	2016	-0.16	[-0.16, -0.15]	-0.18	[-0.20, -0.17]	-0.03	[-0.05, -0.01]	644.92	[642.60, 647.25]	257.73	[252.50, 262.96]	-387.19	[-392.92, -381.47]
Tripura	1993	-0.05	[-0.06, -0.03]	-0.17	[-0.18, -0.15]	-0.12	[-0.14, -0.10]	638.98	[634.12, 643.83]	437.37	[431.65, 443.09]	-201.60	[-209.10, -194.11]
Tripura	1999	-0.04	[-0.06, -0.03]	-0.24	[-0.26, -0.22]	-0.20	[-0.22, -0.17]	630.49	[626.18, 634.80]	252.92	[246.16, 259.69]	-377.57	[-385.59, -369.55]
Tripura	2006	-0.14	[-0.16, -0.13]	-0.21	[-0.24, -0.19]	-0.07	[-0.10, -0.04]	648.50	[643.80, 653.20]	294.74	[286.59, 302.90]	-353.76	[-363.17, -344.35]
Tripura	2016	-0.16	[-0.16, -0.15]	-0.18	[-0.20, -0.17]	-0.03	[-0.05, -0.01]	644.92	[642.60, 647.25]	257.73	[252.50, 262.96]	-387.19	[-392.92, -381.47]
Meghalaya	1993	-0.05	[-0.06, -0.03]	-0.17	[-0.18, -0.15]	-0.12	[-0.14, -0.10]	638.98	[634.12, 643.83]	437.37	[431.65, 443.09]	-201.60	[-209.10, -194.11]
Meghalaya	1999	-0.04	[-0.06, -0.03]	-0.24	[-0.26, -0.22]	-0.20	[-0.22, -0.17]	630.49	[626.18, 634.80]	252.92	[246.16, 259.69]	-377.57	[-385.59, -369.55]
Meghalaya	2006	-0.14	[-0.16, -0.13]	-0.21	[-0.24, -0.19]	-0.07	[-0.10, -0.04]	648.50	[643.80, 653.20]	294.74	[286.59, 302.90]	-353.76	[-363.17, -344.35]
Meghalaya	2016	-0.16	[-0.16, -0.15]	-0.18	[-0.20, -0.17]	-0.03	[-0.05, -0.01]	644.92	[642.60, 647.25]	257.73	[252.50, 262.96]	-387.19	[-392.92, -381.47]
Assam	1993	-0.05	[-0.06, -0.03]	-0.17	[-0.18, -0.15]	-0.12	[-0.14, -0.10]	638.98	[634.12, 643.83]	437.37	[431.65, 443.09]	-201.60	[-209.10, -194.11]
Assam	1999	-0.04	[-0.06, -0.03]	-0.24	[-0.26, -0.22]	-0.20	[-0.22, -0.17]	630.49	[626.18, 634.80]	252.92	[246.16, 259.69]	-377.57	[-385.59, -369.55]
Assam	2006	-0.14	[-0.16, -0.13]	-0.21	[-0.24, -0.19]	-0.07	[-0.10, -0.04]	648.50	[643.80, 653.20]	294.74	[286.59, 302.90]	-353.76	[-363.17, -344.35]
Assam	2016	-0.16	[-0.16, -0.15]	-0.18	[-0.20, -0.17]	-0.03	[-0.05, -0.01]	644.92	[642.60, 647.25]	257.73	[252.50, 262.96]	-387.19	[-392.92, -381.47]
West Bengal	1993	-0.05	[-0.06, -0.03]	-0.17	[-0.18, -0.15]	-0.12	[-0.14, -0.10]	638.98	[634.12, 643.83]	437.37	[431.65, 443.09]	-201.60	[-209.10, -194.11]
West Bengal	1999	-0.04	[-0.06, -0.03]	-0.24	[-0.26, -0.22]	-0.20	[-0.22, -0.17]	630.49	[626.18, 634.80]	252.92	[246.16, 259.69]	-377.57	[-385.59, -369.55]
West Bengal	2006	-0.14	[-0.16, -0.13]	-0.21	[-0.24, -0.19]	-0.07	[-0.10, -0.04]	648.50	[643.80, 653.20]	294.74	[286.59, 302.90]	-353.76	[-363.17, -344.35]
West Bengal	2016	-0.16	[-0.16, -0.15]	-0.18	[-0.20, -0.17]	-0.03	[-0.05, -0.01]	644.92	[642.60, 647.25]	257.73	[252.50, 262.96]	-387.19	[-392.92, -381.47]
Orissa	1993	-0.05	[-0.06, -0.03]	-0.17	[-0.18, -0.15]	-0.12	[-0.14, -0.10]	638.98	[634.12, 643.83]	437.37	[431.65, 443.09]	-201.60	[-209.10, -194.11]
Orissa	1999	-0.04	[-0.06, -0.03]	-0.24	[-0.26, -0.22]	-0.20	[-0.22, -0.17]	630.49	[626.18, 634.80]	252.92	[246.16, 259.69]	-377.57	[-385.59, -369.55]
Orissa	2006	-0.14	[-0.16, -0.13]	-0.21	[-0.24, -0.19]	-0.07	[-0.10, -0.04]	648.50	[643.80, 653.20]	294.74	[286.59, 302.90]	-353.76	[-363.17, -344.35]
Orissa	2016	-0.16	[-0.16, -0.15]	-0.18	[-0.20, -0.17]	-0.03	[-0.05, -0.01]	644.92	[642.60, 647.25]	257.73	[252.50, 262.96]	-387.19	[-392.92, -381.47]
Madhya Pradesh & Chhattisgarh	1993	-0.05	[-0.06, -0.03]	-0.17	[-0.18, -0.15]	-0.12	[-0.14, -0.10]	638.98	[634.12, 643.83]	437.37	[431.65, 443.09]	-201.60	[-209.10, -194.11]
Madhya Pradesh & Chhattisgarh	1999	-0.04	[-0.06, -0.03]	-0.24	[-0.26, -0.22]	-0.20	[-0.22, -0.17]	630.49	[626.18, 634.80]	252.92	[246.16, 259.69]	-377.57	[-385.59, -369.55]
Madhya Pradesh & Chhattisgarh	2006	-0.14	[-0.16, -0.13]	-0.21	[-0.24, -0.19]	-0.07	[-0.10, -0.04]	648.50	[643.80, 653.20]	294.74	[286.59, 302.90]	-353.76	[-363.17, -344.35]
Madhya Pradesh & Chhattisgarh	2016	-0.16	[-0.16, -0.15]	-0.18	[-0.20, -0.17]	-0.03	[-0.05, -0.01]	644.92	[642.60, 647.25]	257.73	[252.50, 262.96]	-387.19	[-392.92, -381.47]
Gujarat	1993	-0.05	[-0.06, -0.03]	-0.17	[-0.18, -0.15]	-0.12	[-0.14, -0.10]	638.98	[634.12, 643.83]	437.37	[431.65, 443.09]	-201.60	[-209.10, -194.11]
Gujarat	1999	-0.04	[-0.06, -0.03]	-0.24	[-0.26, -0.22]	-0.20	[-0.22, -0.17]	630.49	[626.18, 634.80]	252.92	[246.16, 259.69]	-377.57	[-385.59, -369.55]
Gujarat	2006	-0.14	[-0.16, -0.13]	-0.21	[-0.24, -0.19]	-0.07	[-0.10, -0.04]	648.50	[643.80, 653.20]	294.74	[286.59, 302.90]	-353.76	[-363.17, -344.35]
Gujarat	2016	-0.16	[-0.16, -0.15]	-0.18	[-0.20, -0.17]	-0.03	[-0.05, -0.01]	644.92	[642.60, 647.25]	257.73	[252.50, 262.96]	-387.19	[-392.92, -381.47]
Maharashtra	1993	-0.05	[-0.06, -0.03]	-0.17	[-0.18, -0.15]	-0.12	[-0.14, -0.10]	638.98	[634.12, 643.83]	437.37	[431.65, 443.09]	-201.60	[-209.10, -194.11]
Maharashtra	1999	-0.04	[-0.06, -0.03]	-0.24	[-0.26, -0.22]	-0.20	[-0.22, -0.17]	630.49	[626.18, 634.80]	252.92	[246.16, 259.69]	-377.57	[-385.59, -369.55]
Maharashtra	2006	-0.14	[-0.16, -0.13]	-0.21	[-0.24, -0.19]	-0.07	[-0.10, -0.04]	648.50	[643.80, 653.20]	294.74	[286.59, 302.90]	-353.76	[-363.17, -344.35]
Maharashtra	2016	-0.16	[-0.16, -0.15]	-0.18	[-0.20, -0.17]	-0.03	[-0.05, -0.01]	644.92	[642.60, 647.25]	257.73	[252.50, 262.96]	-387.19	[-392.92, -381.47]
Andhra Pradesh & Telangana	1993	-0.05	[-0.06, -0.03]	-0.17	[-0.18, -0.15]	-0.12	[-0.14, -0.10]	638.98	[634.12, 643.83]	437.37	[431.65, 443.09]	-201.60	[-209.10, -194.11]

		Wealth z-score						Age (days)					
State	Year	Included		Missing		Difference		Included		Missing		Difference	
Andhra Pradesh & Telangana	1999	-0.04	[-0.06, -0.03]	-0.24	[-0.26, -0.22]	-0.20	[-0.22, -0.17]	630.49	[626.18, 634.80]	252.92	[246.16, 259.69]	-377.57	[-385.59, -369.55]
Andhra Pradesh & Telangana	2006	-0.14	[-0.16, -0.13]	-0.21	[-0.24, -0.19]	-0.07	[-0.10, -0.04]	648.50	[643.80, 653.20]	294.74	[286.59, 302.90]	-353.76	[-363.17, -344.35]
Andhra Pradesh & Telangana	2016	-0.16	[-0.16, -0.15]	-0.18	[-0.20, -0.17]	-0.03	[-0.05, -0.01]	644.92	[642.60, 647.25]	257.73	[252.50, 262.96]	-387.19	[-392.92, -381.47]
Karnataka	1993	-0.05	[-0.06, -0.03]	-0.17	[-0.18, -0.15]	-0.12	[-0.14, -0.10]	638.98	[634.12, 643.83]	437.37	[431.65, 443.09]	-201.60	[-209.10, -194.11]
Karnataka	1999	-0.04	[-0.06, -0.03]	-0.24	[-0.26, -0.22]	-0.20	[-0.22, -0.17]	630.49	[626.18, 634.80]	252.92	[246.16, 259.69]	-377.57	[-385.59, -369.55]
Karnataka	2006	-0.14	[-0.16, -0.13]	-0.21	[-0.24, -0.19]	-0.07	[-0.10, -0.04]	648.50	[643.80, 653.20]	294.74	[286.59, 302.90]	-353.76	[-363.17, -344.35]
Karnataka	2016	-0.16	[-0.16, -0.15]	-0.18	[-0.20, -0.17]	-0.03	[-0.05, -0.01]	644.92	[642.60, 647.25]	257.73	[252.50, 262.96]	-387.19	[-392.92, -381.47]
Goa, Daman & Diu	1993	-0.05	[-0.06, -0.03]	-0.17	[-0.18, -0.15]	-0.12	[-0.14, -0.10]	638.98	[634.12, 643.83]	437.37	[431.65, 443.09]	-201.60	[-209.10, -194.11]
Goa, Daman & Diu	1999	-0.04	[-0.06, -0.03]	-0.24	[-0.26, -0.22]	-0.20	[-0.22, -0.17]	630.49	[626.18, 634.80]	252.92	[246.16, 259.69]	-377.57	[-385.59, -369.55]
Goa, Daman & Diu	2006	-0.14	[-0.16, -0.13]	-0.21	[-0.24, -0.19]	-0.07	[-0.10, -0.04]	648.50	[643.80, 653.20]	294.74	[286.59, 302.90]	-353.76	[-363.17, -344.35]
Goa, Daman & Diu	2016	-0.16	[-0.16, -0.15]	-0.18	[-0.20, -0.17]	-0.03	[-0.05, -0.01]	644.92	[642.60, 647.25]	257.73	[252.50, 262.96]	-387.19	[-392.92, -381.47]
Kerala	1993	-0.05	[-0.06, -0.03]	-0.17	[-0.18, -0.15]	-0.12	[-0.14, -0.10]	638.98	[634.12, 643.83]	437.37	[431.65, 443.09]	-201.60	[-209.10, -194.11]
Kerala	1999	-0.04	[-0.06, -0.03]	-0.24	[-0.26, -0.22]	-0.20	[-0.22, -0.17]	630.49	[626.18, 634.80]	252.92	[246.16, 259.69]	-377.57	[-385.59, -369.55]
Kerala	2006	-0.14	[-0.16, -0.13]	-0.21	[-0.24, -0.19]	-0.07	[-0.10, -0.04]	648.50	[643.80, 653.20]	294.74	[286.59, 302.90]	-353.76	[-363.17, -344.35]
Kerala	2016	-0.16	[-0.16, -0.15]	-0.18	[-0.20, -0.17]	-0.03	[-0.05, -0.01]	644.92	[642.60, 647.25]	257.73	[252.50, 262.96]	-387.19	[-392.92, -381.47]
Tamil Nadu	1993	-0.05	[-0.06, -0.03]	-0.17	[-0.18, -0.15]	-0.12	[-0.14, -0.10]	638.98	[634.12, 643.83]	437.37	[431.65, 443.09]	-201.60	[-209.10, -194.11]
Tamil Nadu	1999	-0.04	[-0.06, -0.03]	-0.24	[-0.26, -0.22]	-0.20	[-0.22, -0.17]	630.49	[626.18, 634.80]	252.92	[246.16, 259.69]	-377.57	[-385.59, -369.55]
Tamil Nadu	2006	-0.14	[-0.16, -0.13]	-0.21	[-0.24, -0.19]	-0.07	[-0.10, -0.04]	648.50	[643.80, 653.20]	294.74	[286.59, 302.90]	-353.76	[-363.17, -344.35]
Tamil Nadu	2016	-0.16	[-0.16, -0.15]	-0.18	[-0.20, -0.17]	-0.03	[-0.05, -0.01]	644.92	[642.60, 647.25]	257.73	[252.50, 262.96]	-387.19	[-392.92, -381.47]

Notes: 95% confidence intervals are shown in brackets. Estimates were weighted using sampling weights and confidence intervals were adjusted for clustering at the PSU-level.

Table S3. Prevalence of underweight and average annual reduction (AAR)

	Year				
State	1993	1999	2006	2016	AAR
Pooled	49.1 [48.1, 50.1]	44.1 [43.2, 45.0]	41.8 [40.7, 42.8]	34.5 [34.1, 34.9]	0.59 [0.55, 0.64]
Andhra Pradesh & Telangana	42.8 [39.6, 45.9]	35.1 [31.6, 38.6]	31.5 [27.6, 35.4]	27.5 [25.5, 29.4]	0.65 [0.49, 0.81]
Arunachal Pradesh	33.3 [28.2, 38.5]	22.8 [17.0, 28.6]	31.6 [26.1, 37.1]	19.4 [17.1, 21.8]	0.59 [0.35, 0.83]
Assam	46.1 [41.4, 50.7]	36.7 [31.1, 42.3]	37.0 [32.0, 42.0]	28.8 [27.2, 30.3]	0.75 [0.53, 0.96]
Bihar & Jharkhand	60.6 [56.8, 64.3]	54.6 [52.2, 57.0]	55.7 [52.9, 58.5]	42.7 [41.8, 43.6]	0.75 [0.59, 0.91]
Delhi	37.4 [34.0, 40.8]	31.2 [25.6, 36.8]	26.5 [22.2, 30.9]	28.8 [24.5, 33.1]	0.36 [0.13, 0.60]
Goa, Daman & Diu	30.1 [26.0, 34.1]	21.6 [17.1, 26.0]	21.5 [16.9, 26.1]	27.1 [21.0, 33.3]	0.12 [-0.19, 0.44]
Gujarat	44.2 [40.8, 47.5]	43.4 [39.7, 47.1]	42.7 [38.8, 46.6]	37.0 [35.0, 39.0]	0.31 [0.14, 0.48]
Haryana	31.7 [28.5, 34.9]	32.0 [28.7, 35.3]	38.9 [34.5, 43.4]	30.7 [29.0, 32.5]	0.04 [-0.12, 0.20]
Himachal Pradesh	39.0 [36.1, 42.0]	38.5 [34.3, 42.6]	31.2 [26.4, 36.0]	20.0 [17.4, 22.7]	0.79 [0.62, 0.95]
Jammu & Kashmir	35.8 [31.8, 39.7]	31.0 [26.9, 35.0]	26.0 [22.2, 29.8]	16.7 [15.1, 18.4]	0.81 [0.63, 0.99]
Karnataka	47.3 [44.2, 50.5]	39.9 [36.2, 43.6]	35.7 [32.0, 39.4]	33.9 [31.7, 36.1]	0.59 [0.42, 0.76]
Kerala	22.5 [19.5, 25.5]	22.5 [18.9, 26.2]	22.7 [18.9, 26.5]	19.0 [16.5, 21.5]	0.15 [-0.01, 0.31]
Madhya Pradesh & Chhattisgarh	57.1 [54.0, 60.3]	53.3 [50.6, 56.1]	55.9 [53.2, 58.6]	40.6 [39.7, 41.5]	0.68 [0.55, 0.82]
Maharashtra	48.9 [45.6, 52.3]	45.9 [42.6, 49.3]	35.3 [31.7, 38.8]	33.6 [31.7, 35.6]	0.67 [0.50, 0.84]
Manipur	21.2 [16.2, 26.2]	20.2 [15.8, 24.6]	19.6 [16.5, 22.6]	11.6 [10.2, 12.9]	0.42 [0.20, 0.65]
Meghalaya	39.0 [31.6, 46.5]	29.8 [25.1, 34.5]	54.1 [47.8, 60.3]	27.5 [25.2, 29.8]	0.51 [0.17, 0.86]
Mizoram	20.4 [15.8, 25.0]	19.9 [15.4, 24.4]	15.3 [12.0, 18.6]	11.0 [8.9, 13.0]	0.40 [0.19, 0.62]
Nagaland	22.3 [17.8, 26.7]	20.4 [16.5, 24.4]	25.3 [21.7, 29.0]	15.1 [13.1, 17.2]	0.31 [0.10, 0.52]
Orissa	50.4 [46.4, 54.4]	51.1 [48.2, 54.1]	40.5 [36.3, 44.7]	33.4 [31.7, 35.0]	0.73 [0.55, 0.92]
Punjab	40.9 [37.5, 44.3]	26.4 [23.0, 29.8]	25.2 [21.7, 28.7]	22.9 [20.9, 24.9]	0.79 [0.62, 0.96]
Rajasthan	44.4 [40.3, 48.5]	48.4 [46.0, 50.8]	38.2 [34.5, 42.0]	35.1 [33.8, 36.4]	0.39 [0.21, 0.58]
Sikkim	NA	17.6 [13.8, 21.4]	18.1 [13.4, 22.7]	12.5 [9.6, 15.4]	0.31 [0.02, 0.59]
Tamil Nadu	40.8 [37.5, 44.2]	31.4 [27.8, 35.0]	28.5 [24.3, 32.6]	25.0 [23.4, 26.6]	0.68 [0.52, 0.84]
Tripura	40.9 [34.9, 47.0]	40.7 [33.3, 48.2]	37.5 [30.7, 44.3]	23.5 [19.9, 27.1]	0.78 [0.46, 1.09]
Uttar Pradesh & Uttaranchal	54.0 [51.9, 56.1]	48.7 [46.5, 51.0]	42.7 [40.7, 44.7]	38.2 [37.4, 39.0]	0.66 [0.57, 0.75]
West Bengal	53.0 [49.7, 56.3]	46.1 [42.3, 49.9]	38.7 [34.8, 42.5]	28.6 [26.6, 30.6]	1.05 [0.88, 1.22]

Notes: 95% confidence intervals are shown in brackets. Estimates were weighted using sampling weights and confidence intervals were adjusted for clustering at the PSU-level.

Table S4. Prevalence of stunting and average annual reduction (AAR)

	Year				
State	1993	1999	2006	2016	AAR
Pooled	53.3 [52.2, 54.4]	51.6 [50.7, 52.4]	45.5 [44.6, 46.5]	36.2 [35.8, 36.6]	0.71 [0.66, 0.76]
Andhra Pradesh & Telangana	NA	47.8 [44.2, 51.4]	41.6 [37.5, 45.6]	27.4 [25.4, 29.4]	1.22 [0.97, 1.46]
Arunachal Pradesh	55.8 [49.6, 61.9]	30.7 [24.2, 37.2]	38.3 [33.2, 43.3]	27.0 [24.6, 29.3]	1.23 [0.95, 1.50]
Assam	58.0 [54.0, 62.0]	55.9 [49.6, 62.3]	42.9 [38.1, 47.6]	34.2 [32.7, 35.8]	1.02 [0.84, 1.21]
Bihar & Jharkhand	59.8 [56.5, 63.2]	57.2 [54.9, 59.5]	49.8 [47.0, 52.6]	43.8 [42.8, 44.8]	0.68 [0.53, 0.82]
Delhi	46.8 [43.7, 49.8]	43.9 [38.8, 49.0]	41.4 [35.9, 47.0]	33.9 [29.1, 38.6]	0.54 [0.31, 0.78]
Goa, Daman & Diu	35.6 [31.0, 40.2]	22.9 [18.3, 27.6]	27.3 [22.9, 31.7]	20.4 [14.3, 26.4]	0.65 [0.33, 0.97]
Gujarat	51.3 [48.4, 54.3]	52.3 [48.9, 55.8]	50.3 [46.8, 53.7]	35.8 [33.8, 37.8]	0.67 [0.51, 0.82]
Haryana	50.8 [47.3, 54.3]	56.1 [52.4, 59.8]	44.1 [39.5, 48.7]	33.0 [31.1, 35.0]	0.79 [0.61, 0.97]
Himachal Pradesh	NA	48.1 [44.1, 52.1]	34.2 [29.1, 39.3]	22.6 [19.7, 25.5]	1.47 [1.19, 1.75]
Jammu & Kashmir	45.2 [41.2, 49.1]	46.1 [41.8, 50.5]	32.5 [27.6, 37.5]	26.0 [24.2, 27.8]	0.81 [0.63, 1.00]
Karnataka	48.1 [45.2, 51.0]	42.6 [39.0, 46.2]	42.1 [37.8, 46.4]	35.3 [32.7, 37.8]	0.57 [0.40, 0.74]
Kerala	33.8 [30.1, 37.4]	29.4 [24.9, 33.9]	27.2 [23.3, 31.1]	21.0 [18.3, 23.7]	0.53 [0.34, 0.72]
Madhya Pradesh & Chhattisgarh	NA	56.0 [53.7, 58.4]	48.7 [45.9, 51.4]	38.6 [37.6, 39.5]	0.99 [0.85, 1.14]
Maharashtra	47.6 [44.5, 50.7]	47.3 [43.9, 50.7]	45.7 [42.1, 49.4]	31.8 [29.8, 33.8]	0.69 [0.53, 0.85]
Manipur	34.9 [29.9, 40.0]	38.9 [34.2, 43.7]	31.4 [28.0, 34.8]	27.0 [25.0, 29.0]	0.35 [0.11, 0.59]
Meghalaya	53.0 [44.9, 61.2]	48.8 [41.7, 55.8]	44.3 [37.9, 50.7]	37.6 [34.9, 40.4]	0.69 [0.30, 1.07]
Mizoram	42.2 [36.0, 48.5]	41.2 [36.4, 46.1]	35.9 [29.6, 42.2]	24.2 [20.8, 27.7]	0.77 [0.46, 1.07]
Nagaland	34.3 [28.7, 39.9]	38.6 [33.9, 43.4]	35.1 [31.6, 38.6]	23.3 [21.1, 25.4]	0.47 [0.21, 0.73]
Orissa	50.6 [46.3, 54.9]	49.8 [46.7, 52.9]	45.5 [40.7, 50.2]	33.3 [31.7, 34.9]	0.74 [0.55, 0.94]
Punjab	45.4 [41.7, 49.1]	45.3 [41.0, 49.6]	35.1 [31.1, 39.2]	25.7 [23.6, 27.8]	0.86 [0.68, 1.04]
Rajasthan	46.2 [42.6, 49.9]	58.8 [56.5, 61.0]	39.2 [35.4, 43.1]	36.4 [35.2, 37.7]	0.42 [0.25, 0.58]
Sikkim	NA	36.7 [30.9, 42.4]	35.5 [29.5, 41.5]	27.0 [22.4, 31.6]	0.58 [0.14, 1.02]
Tamil Nadu	NA	35.3 [31.5, 39.1]	31.4 [27.5, 35.3]	27.8 [26.1, 29.6]	0.46 [0.20, 0.72]
Tripura	51.5 [46.0, 57.1]	44.3 [36.8, 51.8]	36.5 [30.2, 42.7]	19.8 [16.4, 23.2]	1.41 [1.12, 1.70]
Uttar Pradesh & Uttaranchal	60.0 [57.8, 62.3]	61.8 [59.7, 63.9]	52.6 [50.6, 54.6]	42.8 [42.0, 43.6]	0.72 [0.62, 0.82]
West Bengal	NA	51.1 [47.2, 55.1]	41.6 [38.0, 45.2]	31.4 [29.2, 33.5]	1.19 [0.92, 1.46]

Notes: 95% confidence intervals are shown in brackets. Estimates were weighted using sampling weights and confidence intervals were adjusted for clustering at the PSU-level.

Table S5. Prevalence of wasting and average annual reduction (AAR)

	Year				
State	1993	1999	2006	2016	AAR
Pooled	26.0 [25.0, 27.1]	21.3 [20.5, 22.1]	24.4 [23.5, 25.4]	24.7 [24.4, 25.1]	0.05 [0.01, 0.10]
Andhra Pradesh & Telangana	NA	12.5 [10.1, 14.9]	16.5 [13.1, 19.8]	21.8 [19.9, 23.6]	-0.55 [-0.73, -0.37]
Arunachal Pradesh	15.5 [9.8, 21.2]	12.5 [7.7, 17.3]	17.0 [11.9, 22.1]	19.5 [17.0, 21.9]	-0.17 [-0.43, 0.10]
Assam	15.9 [12.6, 19.2]	18.0 [12.0, 24.0]	18.3 [15.1, 21.5]	20.2 [18.6, 21.7]	-0.18 [-0.34, -0.02]
Bihar & Jharkhand	35.9 [32.6, 39.3]	27.8 [25.1, 30.4]	35.9 [33.0, 38.9]	27.3 [26.4, 28.2]	0.36 [0.22, 0.51]
Delhi	17.9 [15.0, 20.8]	17.2 [11.8, 22.6]	17.4 [13.2, 21.6]	17.5 [12.1, 22.8]	0.02 [-0.24, 0.28]
Goa, Daman & Diu	20.0 [16.5, 23.6]	17.2 [13.3, 21.2]	14.0 [10.6, 17.4]	24.8 [18.1, 31.4]	-0.20 [-0.52, 0.12]
Gujarat	25.4 [22.2, 28.7]	22.2 [19.1, 25.4]	24.6 [21.6, 27.5]	30.4 [28.1, 32.6]	-0.21 [-0.38, -0.04]
Haryana	9.2 [7.2, 11.2]	7.1 [4.9, 9.2]	23.9 [19.7, 28.0]	23.8 [21.9, 25.7]	-0.65 [-0.77, -0.52]
Himachal Pradesh	NA	21.2 [17.7, 24.7]	21.9 [17.7, 26.2]	16.5 [14.1, 19.0]	0.27 [0.02, 0.52]
Jammu & Kashmir	19.8 [16.6, 23.1]	16.2 [13.1, 19.4]	18.8 [15.5, 22.0]	15.6 [13.7, 17.4]	0.18 [0.02, 0.34]
Karnataka	25.5 [22.8, 28.2]	26.4 [22.8, 30.1]	20.9 [17.5, 24.3]	28.8 [26.0, 31.6]	-0.15 [-0.32, 0.03]
Kerala	16.5 [13.7, 19.2]	13.9 [11.1, 16.6]	17.1 [13.8, 20.5]	18.1 [15.6, 20.6]	-0.07 [-0.22, 0.08]
Madhya Pradesh & Chhattisgarh	NA	27.0 [24.2, 29.7]	37.4 [34.3, 40.5]	30.3 [29.4, 31.2]	-0.19 [-0.35, -0.03]
Maharashtra	29.1 [26.2, 32.1]	27.9 [24.9, 30.9]	18.8 [16.1, 21.5]	28.2 [26.4, 30.1]	0.04 [-0.11, 0.19]
Manipur	10.3 [6.7, 13.9]	10.2 [7.3, 13.2]	10.8 [8.5, 13.0]	7.4 [6.2, 8.6]	0.13 [-0.04, 0.29]
Meghalaya	19.6 [12.8, 26.3]	16.9 [12.4, 21.3]	29.0 [22.0, 36.1]	19.0 [16.7, 21.3]	0.03 [-0.29, 0.34]
Mizoram	4.1 [1.0, 7.2]	14.2 [8.5, 19.9]	11.1 [7.5, 14.7]	7.6 [6.1, 9.2]	-0.15 [-0.30, -0.00]
Nagaland	16.3 [11.9, 20.7]	13.1 [9.1, 17.1]	18.5 [15.2, 21.7]	13.0 [11.0, 15.1]	0.14 [-0.07, 0.35]
Orissa	32.5 [28.9, 36.1]	31.3 [28.2, 34.5]	23.0 [19.5, 26.6]	24.3 [22.9, 25.8]	0.35 [0.19, 0.52]
Punjab	24.9 [22.0, 27.8]	9.2 [6.9, 11.5]	11.2 [8.0, 14.3]	17.5 [15.5, 19.5]	0.32 [0.17, 0.47]
Rajasthan	24.9 [21.5, 28.3]	17.9 [15.9, 20.0]	26.7 [23.7, 29.8]	25.8 [24.6, 27.0]	-0.04 [-0.19, 0.12]
Sikkim	NA	6.4 [3.4, 9.4]	12.9 [8.2, 17.7]	16.6 [12.9, 20.4]	-0.62 [-0.91, -0.33]
Tamil Nadu	NA	23.2 [20.3, 26.2]	26.2 [22.0, 30.4]	22.3 [20.6, 24.0]	0.06 [-0.15, 0.27]
Tripura	25.1 [19.5, 30.7]	18.0 [13.5, 22.5]	24.9 [19.7, 30.1]	19.6 [16.2, 23.1]	0.25 [-0.05, 0.54]
Uttar Pradesh & Uttaranchal	25.3 [23.1, 27.4]	16.7 [14.9, 18.6]	18.8 [17.0, 20.6]	22.2 [21.5, 22.9]	0.13 [0.04, 0.22]
West Bengal	NA	19.7 [16.7, 22.8]	22.3 [19.0, 25.5]	22.7 [20.7, 24.7]	-0.18 [-0.40, 0.04]

Notes: 95% confidence intervals are shown in brackets. Estimates were weighted using sampling weights and confidence intervals were adjusted for clustering at the PSU-level.

Table S6. Poor-rich gap in 1993 and change in poor-rich gap between 1993 and 2016

	Prevalence of underweight		Prevalence of stunting		Prevalence of wasting	
	1993 survey	2016 × Poorest	1993 survey	2016 × Poorest	1993 survey	2016 × Poorest
Pooled	26.7	-0.4	21.2	3.7	10.6	-0.8
-	[24.1, 29.3]	[-3.3, 2.4]	[18.2, 24.2]	[0.5, 6.9]	[7.7, 13.5]	[-4.0, 2.3]
Andhra Pradesh & Telangana	24.4	0.2	24.9	-3.3	5.1	2.5
-	[15.0, 33.9]	[-10.6, 11.1]	[14.4, 35.3]	[-15.5, 8.9]	[-2.3, 12.5]	[-7.2, 12.1]
Arunachal Pradesh	4.7	14.7	-12.4	27.9	-0.3	10.0
-	[-13.2, 22.7]	[-4.3, 33.7]	[-30.6, 5.8]	[8.5, 47.4]	[-20.7, 20.2]	[-11.6, 31.7]
Assam	27.0	3.3	23.5	5.7	8.0	4.6
-	[15.3, 38.7]	[-9.1, 15.7]	[11.2, 35.9]	[-7.3, 18.8]	[0.2, 15.8]	[-4.5, 13.6]
Bihar & Jharkhand	12.2	9.2	10.9	9.7	6.6	3.7
-	[3.9, 20.5]	[0.6, 17.9]	[2.3, 19.5]	[0.6, 18.7]	[-1.8, 15.0]	[-5.1, 12.4]
Delhi	35.3	-18.0	32.8	-9.6	6.2	-10.6
-	[25.9, 44.7]	[-36.5, 0.5]	[23.4, 42.1]	[-28.9, 9.8]	[-2.1, 14.6]	[-26.8, 5.7]
Goa, Daman & Diu	30.4	-12.3	39.5	-11.5	13.0	-12.0
-	[19.5, 41.4]	[-36.2, 11.6]	[28.1, 50.9]	[-29.1, 6.2]	[2.3, 23.8]	[-35.1, 11.0]
Gujarat	34.5	-8.9	19.9	2.7	15.7	-6.8
-	[26.4, 42.6]	[-18.8, 0.9]	[12.4, 27.4]	[-6.6, 12.1]	[5.6, 25.7]	[-18.9, 5.3]
Haryana	29.5	-16.0	21.0	-4.2	6.7	-3.4
-	[19.9, 39.0]	[-26.7, -5.2]	[11.6, 30.3]	[-15.1, 6.6]	[-0.6, 14.0]	[-12.6, 5.8]
Himachal Pradesh	20.8	-10.1	20.1	-2.1	19.2	-18.5
-	[11.1, 30.6]	[-22.6, 2.5]	[6.7, 33.4]	[-17.5, 13.2]	[6.9, 31.6]	[-33.2, -3.9]
Jammu & Kashmir	26.2	-9.4	25.8	-9.7	18.2	-15.3
-	[15.5, 37.0]	[-21.1, 2.2]	[13.5, 38.0]	[-23.2, 3.7]	[7.9, 28.6]	[-26.8, -3.8]
Karnataka	30.0	-14.9	31.4	-16.0	8.3	0.8
-	[21.6, 38.4]	[-26.7, -3.1]	[23.1, 39.6]	[-28.6, -3.4]	[0.4, 16.2]	[-11.8, 13.4]
Kerala	24.1	-10.7	28.5	-20.4	13.1	-9.3
-	[16.3, 31.8]	[-21.5, -0.0]	[18.8, 38.1]	[-32.6, -8.3]	[5.1, 21.1]	[-20.3, 1.7]
Madhya Pradesh & Chhattisgarh	17.7	5.2	26.2	-9.6	20.2	-5.9
-	[8.8, 26.6]	[-4.1, 14.5]	[19.5, 32.9]	[-16.7, -2.4]	[12.8, 27.7]	[-13.8, 2.0]
Maharashtra	36.1	-9.1	26.2	-6.6	15.7	-5.0
-	[26.6, 45.6]	[-20.2, 2.0]	[17.6, 34.9]	[-17.3, 4.2]	[6.3, 25.1]	[-16.5, 6.4]
Manipur	9.5	0.1	15.0	6.7	14.4	-10.7
-	[-6.2, 25.2]	[-16.0, 16.3]	[2.0, 28.1]	[-7.4, 20.8]	[1.7, 27.1]	[-23.9, 2.5]
Meghalaya	17.3	-1.1	6.7	14.0	7.1	-8.7
-	[-0.9, 35.5]	[-20.7, 18.4]	[-14.0, 27.4]	[-7.7, 35.8]	[-11.9, 26.0]	[-28.4, 11.1]
Mizoram	5.2	5.0	15.7	9.3	0.2	9.1
-	[-10.5, 20.9]	[-11.9, 22.0]	[-1.5, 32.9]	[-10.1, 28.6]	[-13.0, 13.3]	[-4.6, 22.7]
Nagaland	11.7	2.4	20.2	0.1	11.2	3.2
-	[0.4, 23.1]	[-10.7, 15.5]	[3.9, 36.5]	[-17.4, 17.6]	[-2.0, 24.4]	[-11.7, 18.1]
Orissa	22.4	9.1	16.1	8.6	9.8	7.5
-	[11.2, 33.7]	[-3.0, 21.1]	[7.7, 24.6]	[-1.1, 18.3]	[-0.5, 20.2]	[-3.6, 18.6]
Punjab	27.5	-14.8	24.4	-7.3	16.3	-10.3
-	[18.4, 36.6]	[-26.4, -3.3]	[14.3, 34.4]	[-19.1, 4.5]	[7.9, 24.7]	[-21.1, 0.6]
Rajasthan	4.8	22.3	2.7	17.7	0.4	16.5
-	[-5.3, 14.8]	[11.6, 33.1]	[-6.3, 11.8]	[8.0, 27.5]	[-8.6, 9.4]	[6.8, 26.2]
Sikkim	13.2	-7.6	26.9	-6.3	3.4	-3.6
-	[2.8, 23.6]	[-22.0, 6.8]	[13.1, 40.8]	[-25.2, 12.6]	[-6.9, 13.7]	[-18.7, 11.6]
Tamil Nadu	26.4	-13.1	30.5	-14.7	7.9	-4.1
-	[16.1, 36.8]	[-24.5, -1.6]	[22.1, 39.0]	[-24.8, -4.7]	[-0.3, 16.1]	[-14.0, 5.8]
Tripura	31.4	-11.4	39.9	-16.4	23.3	-12.1
-	[14.3, 48.6]	[-31.0, 8.3]	[21.6, 58.1]	[-36.8, 3.9]	[4.5, 42.2]	[-33.4, 9.3]
Uttar Pradesh & Uttaranchal	12.1	12.0	8.8	18.4	4.4	2.4
-	[5.7, 18.4]	[5.3, 18.7]	[2.4, 15.3]	[11.6, 25.3]	[-1.1, 9.9]	[-3.6, 8.4]
West Bengal	35.2	-8.2	41.6	-19.6	15.6	-1.8
-	[26.9, 43.4]	[-18.2, 1.8]	[32.1, 51.1]	[-30.8, -8.4]	[6.7, 24.4]	[-12.2, 8.7]

Notes: Percentage point differences are shown. 95% confidence intervals are shown in brackets. Estimates were weighted using sampling weights and confidence intervals were adjusted for clustering at the PSU-level.
